# Effect of Polymer Aging on Uptake/Release Kinetics of Metal Ions and Organic Molecules by Micro- and Nanoplastics: Implications for the Bioavailability of the Associated Compounds

**DOI:** 10.1021/acs.est.3c05148

**Published:** 2023-10-19

**Authors:** Raewyn M. Town, Herman P. van Leeuwen, Jérôme F. L. Duval

**Affiliations:** † ECOSPHERE, Department of Biology, Universiteit Antwerpen, Groenenborgerlaan 171, 2020 Antwerpen, Belgium; ‡ Physical Chemistry and Soft Matter, Wageningen University & Research, Stippeneng 4, 6708 WE Wageningen, The Netherlands; § Université de Lorraine, CNRS, LIEC, F-54000 Nancy, France

**Keywords:** transient flux, intraparticulate diffusion, polymers, aquatic contamination

## Abstract

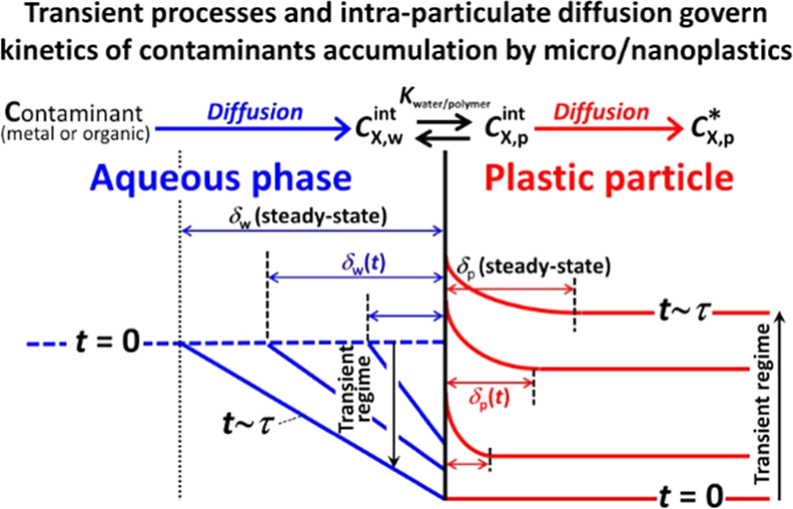

The main driver of the potential toxicity of micro- and nanoplastics toward biota is often the release of compounds initially present in the plastic, i.e., polymer additives, as well as environmentally acquired metals and/or organic contaminants. Plastic particles degrade in the environment via various mechanisms and at different rates depending on the particle size/geometry, polymer type, and the prevailing physical and chemical conditions. The rate and extent of polymer degradation have obvious consequences for the uptake/release kinetics and, thus, the bioavailability of compounds associated with plastic particles. Herein, we develop a theoretical framework to describe the uptake and release kinetics of metal ions and organic compounds by plastic particles and apply it to the analysis of experimental data for pristine and aged micro- and nanoplastics. In particular, we elucidate the contribution of transient processes to the overall kinetics of plastic reactivity toward aquatic contaminants and demonstrate the paramount importance of intraparticulate contaminant diffusion.

## Abstract 2

Aging of plastic particles can affect the extent and rate at which they interact with other environmental contaminants. This work analyzes the involved processes and their relevance to aquatic systems.

## Introduction

Environmental pollution by plastic materials is a global concern, and many questions remain about their potential adverse effects on biota. Annual surveys of the environmental accumulation rates of plastic fragments indicate that the average size of plastic particles in the environment is decreasing and the abundance of microplastics is increasing.^1^ In general, particles with dimensions in the micrometer and nanometer size ranges—microplastics and nanoplastics, respectively—are of most concern. These small particles can be ingested by a wide range of organisms, and those with radii less than ca. 50 nm can pass through biological membranes.^2^ Plastic particles carry a cargo of associated inorganic and organic contaminants, comprising polymer additives as well as environmentally acquired contaminants. The compounds associated with plastic particles are proposed to be the main drivers of toxicity, rather than the polymer backbone per se.^3^ Demonstration of the toxicity of compounds associated with plastics is generally done by exposing organisms directly to compounds that have been previously leached from plastics.^4−7^ Such approaches typically show that leachates from aged plastics exert greater toxicity than those from pristine materials.^4,5^ However, the interpretation of the results in terms of potential ecotoxicological risk ignores the fundamental difference in exposure kinetics between dissolved and particle-associated compounds.^8−11^ It is thus crucial to understand the uptake and release kinetics of polymer additives and environmentally acquired compounds to understand the role played by plastic particles in influencing the environmental fate and bioavailability of associated compounds.

Upon release into the environment, plastic particles are exposed to a range of conditions, abiotic and biotic processes, which alter their physicochemical properties and thus also modify the uptake/release kinetics of associated compounds. Polymer degradation usually involves a combination of (bio)chemical processes which solubilize some components and embrittle the matrix, followed by fragmentation due to physical forces.^12,13^ Degradation can occur via bulk erosion or surface erosion,^14^ and for a given polymer, there is a thickness above which surface erosion dominates.^15,16^ Furthermore, the mode of degradation can transition from surface to bulk erosion over the course of the degradation process. In the context of drug delivery, detailed models have been constructed to describe concomitant polymer degradation and drug release kinetics under well-defined conditions.^15^ However, it is extremely challenging to develop a mechanistic rationale for polymer degradation rates in the environment due to the wide range of polymer types and formulations involved, together with the diversity of contributing physical, chemical, and biological processes each with their own kinetic features. Nevertheless, attempts have been made to empirically correlate polymer properties to environmental degradation susceptibility,^17,18^ albeit not always successfully,^19^ and to predict the environmental lifetimes of plastic objects by assuming that surface erosion is the only process involved.^20^

Due to the environmental persistence of petrochemical-based plastics, there is a drive toward the development of more readily (bio)degradable plastics. Nevertheless, degradable plastics still include a range of potentially toxic additives to confer desired properties,^21^ and their more rapid degradation under environmental conditions^22^ implies that the associated compounds will be released over a shorter time scale. Thus, within a given time frame, (bio)degradable plastics have the potential to expose organisms to higher concentrations of toxic compounds—either externally or internally—than those associated with more refractory plastics.

In the literature, the uptake/release rates of compounds by plastics are typically analyzed by empirical fitting of (pseudo) first-order and (pseudo) second-order kinetics, sometimes with the inclusion of intraparticulate diffusion but without rigorous consideration of transient phenomena.^23,24^ Furthermore, many studies are conducted under conditions of significant bulk depletion of the target compound from the aqueous medium, yet the contribution of the depletion kinetics to the temporal evolution of sorption is ignored, thereby confounding the meaning of the derived parameters.^24−31^

In our previous work, we detailed a framework for describing the uptake/release kinetics of ions and small molecules by plastic particles depending on the size of the particles, as well as other physicochemical properties, including the diffusion coefficient of the target compound in the polymer matrix and its affinity for the polymer backbone.^32,33^ Herein, we extend the approach to take full account of transient processes. We successfully apply the developed theory to the analysis of the uptake/release kinetics of metal ions and organic compounds by a range of pristine and aged micro- and nanoplastic particles, and we explain the failure of common empirical kinetic models in interpreting the sorption of metals and organic contaminants by plastic particles over time.

## Theory

**Figure 1:**
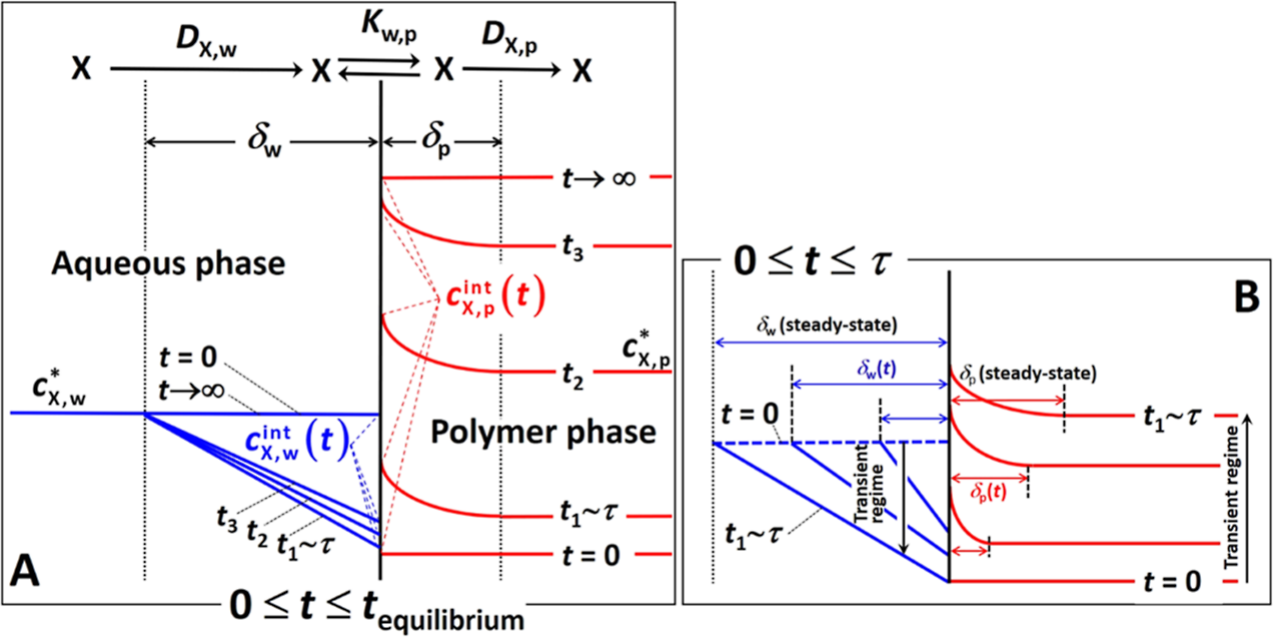
Schematic illustration of the temporal evolution of concentration gradients in the aqueous and polymer phases during (A) steady-state and (B) transient regimes. The nomenclature adopted for the relevant concentrations of X considered in the formalism, i.e., *c*_X,w_^*^, *c*_X,w_^int^, *c*_X,p_^int^, and *c*_X,p_^*^, is specified. See the text for details. Adapted from Bayen et al.^38^

Consider the case where a pristine plastic particle is immersed in an aqueous solution containing the target ionic or molecular contaminant, denoted as X (metal ion or organic compound). For simplicity of illustration, we assume that the bulk concentration of X in the aqueous medium, *c*_X,w_^*^, remains constant throughout the absorption process. At time *t* = 0, the concentration of X in the particle body, *c*_X,p_, is zero, i.e., *c*_X,p_(*t* = 0) = 0. At *t* > 0, diffusion of X into the particle results in the development of concentration gradients on both the aqueous and the particle sides of the particle/water interface (Figure 1). During an initial transient stage, the concentration of X at the aqueous side of the particle/water interface, *c*_X,w_^int^, decreases and that at the particle side, *c*_X,p_^int^, increases. After a characteristic time, τ, a steady-state flux is attained, and the concentration gradients are then constant in the aqueous and polymeric phases over a spatial scale denoted as the diffusion layer thickness, δ. The diffusion layer thickness in the aqueous medium, δ_w_, for spherical micro- and nanosized objects is equal to the particle radius;^34,35^ for larger entities, δ_w_ depends on the hydrodynamic conditions and is typically of the order of 10 μm for moderately stirred solutions.^36^ The diffusion layer thickness within the polymer phase, δ_p_, depends on the diffusion coefficient of the target compound. At times less than τ, the uptake kinetics reflect the relaxation of the flux from transient toward steady-state values.^37^ In the typical environmental setting, the time course of the relaxation of the diffusion process from transient to steady-state will be coupled with relaxation of the polymer structure (due to ongoing aging processes, e.g., photodegradation, mechanical abrasion, etc.) and also with relaxation of all physicochemical interactions between the target analyte and the polymer phase since hydrophobic/electrostatic interactions may well change over time due to relaxation of the polymer backbone structure and aging processes. As formulated herein, all possible contributions to relaxation time are effectively included.

Given that diffusion coefficients within the polymer phase can be very low—typically in the range 10^–14^ to 10^–17^ m^2^ s^–1^ depending on the nature of X and the polymer type^32,33^ – transient flux conditions can be in effect for many hours, even for particles with dimensions of the order of 1 μm. The transient regime corresponds to the time domain over which the diffusion layers are developing (increasing in thickness, Figure 1B), and the diffusive flux as well as the rate constants are functions of time.^37^ We highlight that the magnitude of the transient flux is always greater than that of the steady-state one.

The flux, *J*_w_, of component X from the bulk water to the water/polymer interface (superscript “int”) of a spherical particle is given by
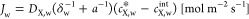
1where *D*_X,w_ is the aqueous diffusion coefficient for X, *a* is the particle radius, *c*_X,w_^*^ is the bulk aqueous concentration of the physicochemical form of X which partitions into the polymeric phase,^39^ and *c*_X,w_^int^ is the concentration of X on the aqueous side of the water/particle interface. The flux, *J*_p_, from the interface to the bulk polymer phase is given by
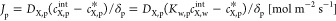
2where *D*_X,p_ is the diffusion coefficient of X in the polymer material, *c*_X,p_^int^ is the concentration of X on the polymer side of the interface, *c*_X,p_^*^ is the concentration of X in the bulk polymer phase, and *K*_w,p_ = *c*_X,p_^int^/*c*_X,w_^int^ is the (dimensionless) water/polymer equilibrium partition coefficient. We highlight that eq 2 holds in the linear part of the sorption isotherm, the so-called Henry regime.

Once steady-state has been achieved, the fluxes *J*_p_ and *J*_w_ necessarily verify at any time *t*
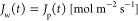
3

By combining eqs 1–3, and simple rearrangement, we obtain the expressions for the concentration of X at the aqueous and polymer sides of the interface, respectively
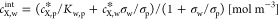
4
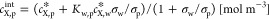
5where σ_w_ and σ_p_ are the mass transport coefficients in the aqueous and polymer phases, respectively
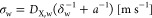
6
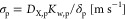
7

Substituting the above expression of the interfacial concentration of X at the water side (eq 4) in the flux expression (eq 1), we obtain for the inherently time-dependent steady-state flux of X
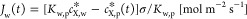
8where σ is the composite mass transport coefficient given by
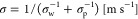
9

The sorption process comprises an initial transient stage, followed by the attainment of a steady state. The differential equation that governs the time dependence (from *t* = 0 to ∞) of the total concentration of X in the polymer phase, *c*_X,p_^tot^(*t*), is given by
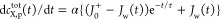
10where τ is the effective time constant for the relaxation of the flux from its initial (transient) value (given by *J*_0_^+^) to the steady-state value (given by *J*_p_(*t*) = *J*_w_(*t*)), and α is the particle surface area/volume ratio given by α = 3/*a*.

Following ref (38), we identify the total concentration *c*_X,p_^tot^(*t*) to *c*_X,p_^*^(*t*). Then, combining eqs 8 and 10, after some algebra, we show that eq 10 can be rewritten in the explicit form
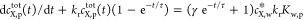
11where the introduced terms γ and *k*_r_ correspond to
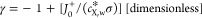
12and
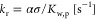
13Once the transient stage is completed (i.e., for *t*/τ ≫ 1), eq 11 simply reduces to
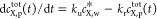
14which corresponds to the simple differential equation associated with the 2-compartment model described by Bayen et al.,^38^ with *k*_r_ and *k*_u_ the rate constants (valid at any time in the steady-state regime) for the uptake and release of X by the particle, respectively, *k*_r_ and *k*_u_ being interrelated by
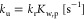
15

The developed expressions of *k*_u_ and *k*_r_, after substitution of eq 9 into the expressions for *k*_r_ (eq 13) and *k*_u_ (eq 15), are given by
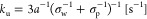
16and
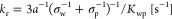
17

Using the relevant boundary condition *c*_X,p_^tot^(*t* = 0) = 0 that reflects the absence of X in the plastic particles at initial time, the solution of the differential equation representing the complete formulation of the problem (eq 11) can be written in the form
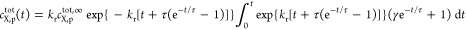
18where *c*_X,p_^tot,∞^ = *c*_X,p_^tot^(*t* → ∞) is the equilibrium plateau value in the kinetic sorption curves, defined by
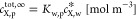
19In the limit where the transient contribution is negligible, i.e., for τ → 0, eq 18 becomes
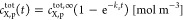
20

In practical cases where τ → 0 (cf. data modeling results), eq 20 must be corrected for the finite nonzero value of *c*_X,p_^tot^ measured at the very beginning of the experiment just after the transient has been completed (in an infinitely fast manner). Denoting this value as , we obtain
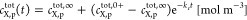
21

The preceding rationale is the true justification of why the quantity  must be adjusted, together with *c*_X,p_^tot,∞^ and *k*_r_, to fit absorption kinetic data to the monoexponential expression of *c*_X,p_^tot^(*t*) given by eq 21.

## Methods

### Experimental Data

Sufficiently documented experimental data were identified in 9 literature reports^40−48^ yielding a total of 64 individual sorption curves. The data comprises the uptake of metal ions and a range of organic compounds by spherical or approximately spherical fragments of pristine and aged polystyrene (PS), polypropylene (PP), polyethylene (PE), polyethylene terephthalate (PET), polyvinyl chloride (PVC), polybutylene adipate terephthalate (PBAT), and polyurethane (PU) (see Table S1 for details of the experimental conditions). The data modeled herein correspond to the linear Henry regime and lie well below saturation of the target compound in the polymer. We estimated that the extent to which the target analyte was depleted from the bulk aqueous medium did not exceed 10%. The aqueous dispersions were typically shaken or stirred during the sorption process; under such hydrodynamic conditions, the aqueous diffusion layer thickness, δ_w_, is of the order of 10^–5^ m;^36^ for particles with dimensions of the order of 10^–5^ m or less, the particle radius *a* is thus the relevant spatial scale for evaluation of the flux *J*_w_ (eq 1) and mass transfer coefficient σ_w_ (eq 6).^34,35^

### Data Fitting Strategies

For each of the experimental data sets, both the approximate monoexponential (eq 21) and involved integral (eq 18) formulations of the problem were adopted as data fitting strategies. More specifically, eqs 18 and 21 were used while considering or not within the fitting procedure the initial 0-absorption point, at *t* = 0, as a “measured” data point (both options were considered). Additional data fitting attempts were made on the basis of eq 20, considering the initial 0-absorption point as part of the ensemble of experimental data subjected to analysis.

Decisions on which approach provided the best reconstruction to the experimental data are based on the robustness of the fitting (i.e., the same outcomes obtained irrespective of the initial input values), the magnitude of the normalized root-mean-square error (NRMSE) (the lower the better; *R*^2^ = 1 – NRMSE), and the constrained physicochemical validity of the computed parameters, e.g., γ must be positive. Herein, NRMSE is evaluated according to , where *N* is the number of experimental data points *y*_*k*=1,...,*N*_^(exp)^ measured at *t* = *t*_*k*=1,...,*N*_, and *y*_*k*_^(theor)^ are the corresponding theoretical predictions after fitting the {*y*_*k*=1,...,*N*_^(exp)^}according to eq 18, 20, or 21. The inclusion or not of the 0-absorption point at *t* = 0 in the fitting of the involved integral eq 18 had negligible effect on the corresponding derived parameters (Tables 1, and S2 and S3 in Supporting Information) simply because eq 18 verifies, by construction, *c*_X,p_^tot^(*t* = 0) = 0. In cases where similar NRMSEs were obtained for all fitting strategies, identification of the best fit was aided by scrutiny of the agreement between the experimental data and the computed values at short times, where the transient contribution is operative. Nevertheless, for some data sets, the best fitting strategy could not be unambiguously identified due to an insufficient number of experimental data points or scatter in the experimental data.

**Table 1:** Fitted and Derived Parameters from Data Analysis According to the Involved Integral and Monoexponential Expressions of *c*_X,p_^tot^(*t*) (eqs 18 and 20 and 21, Respectively)

system	fit^a^^,^^b^	monoexponential				involved integral^e^						
		*k*_r_ (s^–1^)	*K*_w,p_	*c*_X,p_(*t* = 0)/*c*_X,p_(eq)^c^	NRMSE^d^	*k*_r_ (s^–1^)	τ (s)	*K*_w,p_	δ_p_/*a*	*D*_X,p_ (m^2^ s^–^^1^)	*J*_0_^+^/(*c*_X,w_^*^σ)	NRMSE^d^
PS pristine, 2 ppm of Cd^40^	incl 0,0	**1.02** × **10^–^**^**4**^	**9.8**	**–0.03**	**0.031**	**7.58** × **10^–^**^**5**^	**5943**	**9.8**	**1.50** × **10^–^**^**1**^	**3.41** × **10^–^**^**19**^	**1.12**	**0.029**
	excl. 0,0	**1.11** × **10^–^**^**4**^	**9.8**	**–0.08**	**0.031**	7.58 × 10^–^^5^	5943	9.8	1.50 × 10^–^^1^	3.41 × 10^–^^19^	1.12	0.034
	incl 0 (eq 20)	**9.80** × **10^–^**^**5**^	**9.8**	**0.0**	**0.032**							
PS aged 7 day Fenton, 2 ppm of Cd^40^	incl 0,0	3.45 × 10^–^^4^	112.7	0.02	0.062	**4.71** × **10^–^**^**5**^	**1501**	**119.6**	**2.36** × **10^–^**^**2**^	**3.33** × **10^–^**^**20**^	**9.50**	**0.014**
	excl. 0,0	9.45 × 10^–^^5^	118.1	0.46	0.068	4.71 × 10^–^^5^	1501	119.6	2.36 × 10^–^^2^	3.33 × 10^–^^20^	9.50	0.029
	incl 0 (eq 20)	3.55 × 10^–^^4^	112.6	0.0	0.063							
PS pristine, 5 mg/L atrazine^42^	incl 0,0	**7.32** × **10^–^**^**5**^	**114.2**	**0.06**	**0.056**	**5.45** × **10^–^**^**5**^	**193.3**	**118.0**	**3.52** × **10^–^**^**3**^	**1.48** × **10^–^**^**16**^	**20.2**	**0.036**
	excl. 0,0	**5.45** × **10^–^**^**5**^	**118.0**	**0.21**	**0.063**	5.45 × 10^–^^5^	193.7	118.0	3.52 × 10^–^^3^	1.48 × 10^–^^16^	20.1	0.063
	incl 0 (eq 20)	**8.29** × **10^–^**^**5**^	**112.4**	**0.0**	**0.062**							
PS aged, 5 mg/L atrazine^42^	incl 0,0	**9.02** × **10^–^**^**5**^	**132.5**	**0.05**	**0.039**	**6.72** × **10^–^**^**5**^	**190.8**	**136.6**	**4.29** × **10^–^**^**3**^	**2.60** × **10^–^**^**16**^	**15.9**	**0.016**
	excl. 0,0	**6.72** × **10^–^**^**5**^	**136.6**	**0.19**	**0.028**	6.72 × 10^–^^5^	188.2	136.6	4.23 × 10^–^^3^	2.56 × 10^–^^16^	16.1	0.028
	incl 0 (eq 20)	**9.82** × **10^–^**^**5**^	**131.3**	**0.0**	**0.042**							
PS pristine, 1 mg/L Cd^44^	incl 0,0	2.82 × 10^–^^5^	181.1	0.06	0.046	**1.43** × **10^–^**^**5**^	**7798.5**	**200.6**	**3.72** × **10^–^**^**2**^	**1.00** × **10^–^**^**15**^	**3.13**	**0.026**
	excl. 0,0	2.61 × 10^–^^5^	183.3	0.08	0.047	1.43 × 10^–^^5^	7798.5	200.6	3.72 × 10^–^^2^	1.00 × 10^–^^15^	3.13	0.029
	incl 0 (eq 20)	3.31 × 10^–^^5^	176.6	0.0	0.052							
PS aged, 1 mg/L Cd^44^	incl 0,0	5.63 × 10^–^^5^	208.1	0.07	0.034	**3.16** × **10^–^**^**5**^	**4787.8**	**216.8**	**5.05** × **10^–^**^**2**^	**3.01** × **10^–^**^**15**^	**2.79**	**0.010**
	excl. 0,0	4.91 × 10^–^^5^	210.8	0.12	0.030	3.16 × 10^–^^5^	4787.8	216.8	5.05 × 10^–^^2^	3.01 × 10^–^^15^	2.79	0.012
	incl 0 (eq 20)	6.62 × 10^–^^5^	204.9	0.0	0.042							
PP pristine, 10 mg/L sulfamethoxazole^47^	incl 0,0	6.73 × 10^–^^5^	24.2	0.03	0.059	**4.34** × **10^–^**^**11**^	**9413.7**	**7.84 × 10**^**5**^	**1.36** × **10^–^**^**7**^	**6.21** × **10^–^**^**26**^	**58.1**	**0.033**
	excl. 0,0	6.46 × 10^–^^5^	24.2	0.05	0.067	4.33 × 10^–^^11^	9413.7	7.85 × 10^5^	1.36 × 10^–^^7^	6.19 × 10^–^^26^	58.1	0.038
	incl 0 (eq 20)	7.14 × 10^–^^5^	24.1	0.0	0.061							
PP aged, 10 mg/L sulfamethoxazole^47^	incl 0,0	2.38 × 10^–^^5^	55.8	0.11	0.049	**1.54** × **10^–^**^**5**^	**982.3**	**58.5**	**5.06** × **10^–^**^**3**^	**8.21** × **10^–^**^**16**^	**13.0**	**0.016**
	excl. 0,0	1.85 × 10^–^^5^	57.3	0.15	0.033	1.54 × 10^–^^5^	982.3	58.5	5.08 × 10^–^^3^	8.21 × 10^–^^16^	13.0	0.019
	incl 0 (eq 20)	4.11 × 10^–^^5^	53.6	0.0	0.071							
PS pristine, 20 mg/L tetracycline^48^	incl 0,0	2.10 × 10^–^^4^	5.8	0.15	0.105	**3.69** × **10^–^**^**5**^	**422.7**	**6.3**	**5.20** × **10^–^**^**3**^	**1.60** × **10^–^**^**16**^	**31.0**	**0.009**
	excl. 0,0	3.86 × 10^–^^5^	6.3	0.47	0.023	3.69 × 10^–^^5^	422.7	6.3	5.20 × 10^–^^3^	1.60 × 10^–^^16^	31.0	0.018
	incl 0 (eq 20)	3.05 × 10^–^^4^	5.7	0.0	0.113							
PS aged, 20 mg/L tetracycline^48^	incl 0,0	4.32 × 10^–^^4^	9.1	0.06	0.073	**3.96** × **10^–^**^**5**^	**833.9**	**9.8**	**1.10** × **10^–^**^**2**^	**3.64** × **10^–^**^**16**^	**19.9**	**0.017**
	excl. 0,0	8.22 × 10^–^^5^	9.6	0.55	0.070	3.96 × 10^–^^5^	833.9	9.8	1.10 × 10^–^^2^	3.64 × 10^–^^16^	19.9	0.039
	incl 0 (eq 20)	4.74 × 10^–^^4^	9.1	0.0	0.074							

a “Incl 0” means that the 0-absorption point at *t* = 0 is included as a “measured” data point; “excl. 0” means that the 0-absorption point at *t* = 0 is not included as a “measured” data point. The monoexponential fits were performed with eq 21 except for the rows specified with eq 20; the involved integral fits were performed with eq 18. See text for further details.

b Bold font denotes the best fit options. More than one option is bold in cases where similar goodness-of-fit was obtained and/or when data scattering precludes the definitive discarding of the merits of a given fitting strategy as compared to another, cf. e.g. Figure 5.

c *c*_X,p_(eq) corresponds to the equilibrium plateau value *c*_X,p_^tot,∞^. See text for details.

d NRMSE = normalized root-mean-square error; the closer this value is to zero, the better the fit; *R*^2^ = 1 – NRMSE.

e Involved integral fit parameters are only entered for the cases for which a robust fitting of experimental data with eq 18 was obtained.

### Involved Integral Fitting Strategy and Exploitation of Fitted Parameters

Fitting of the experimental data using the integral formulation (eq 18) of *c*_X,p_^tot^(*t*) (corresponding to inclusion of the transient contribution to the flux via the time scale τ), involves the adjustment of the parameters *k*_r_, τ, γ, and *c*_X,p_^tot,∞^. Once these parameters are obtained, the additional quantities *K*_w,p,_*k*_u_, δ_p_, σ_p_, *D*_X,p_, and *J*_0_^+^ involved in the formulation of the kinetics of X absorption by plastic particles can be derived along the lines briefly described below and further elaborated in the Supporting Information. Namely, *K*_w,p_ (dimensionless) is computed from the ratio of the fitted *c*_X,p_^tot,∞^ (mass/mass units) and the known bulk aqueous concentration of X, converted to per mass unit using the density of water (1 kg dm^–3^) (eq 19). From the fitted value of *k*_r_, *k*_u_ then follows simply via eq 15. The fitted relaxation time, τ, is the sum of the involved transient processes in the aqueous phase (eq S1 in Supporting Information), τ_w_ (eq S2), and the polymer phase, τ_p_ (eq S3). For all practical cases considered (see Tables S2 and S3), we have τ_p_ ≫ τ_w_, thus τ ≈ τ_p_. Under such conditions applicable to the data analyzed, here, the expressions for τ can be simplified according to eq S4. Then, knowing the particle radius *a*, *K*_wp_ (obtained from fitting), *k*_r_ (obtained from fitting), and knowing σ_w_ (which implies that we know or have reasonable estimates of *D*_X,w_ and δ_w_), combination of eqs S4 and 17 for τ and *k*_r_, respectively (whose values are known from fitting), leads to estimation of σ_p_ (eq S6) and δ_p_ (eq S7). The obtained values of σ_p_ and δ_p_ can then be used to compute *D*_X,p_ via eq S8. The obtained *D*_X,p_ is considered to back-check the inequality τ_p_/τ_w_ ≫ 1, i.e., that the approximation τ = τ_p_ is valid. Finally, using eq 12 and adopting the values of γ (obtained from fitting) and σ (derived by eq 9 after substitution of the known σ_p_ and σ_w_), we derive the initial flux *J*_0_^+^ that holds within the transient regime.

### Monoexponential Fitting Strategy

Application of the monoexponential expressions (no transient considered, eqs 20 and 21) involves adjusting the parameters *k*_r_, *c*_X,p_^tot,∞^, and  (relevant only for eq 21) to reconstruct the kinetic absorption data. In cases where eqs 20 or 21 provide a satisfactory description of the data, the only parameters that can be obtained using the fitted *k*_r_ and *c*_X,p_^tot,∞^ are *K*_w,p_ (eq 19) and *k*_u_ (eq 15).

The data analyzed herein were obtained in simple aqueous media, i.e., the target compounds were present in their “free” forms and possible complexes could be neglected. Expressions which account for the lability of complexed X species are available in our previous work.^32,33^ Fitting of data to eqs 18, 20, and 21 was performed using the PTC Mathcad Prime 8.0 calculus environment with the Levenberg–Marquardt algorithm for minimization of residues upon adjustment of the parameters detailed above for each fitting strategy.

## Results and Discussion

Depending on the nature of the target analyte X and the plastic particle (polymer type, pristine or aged), the experimental data were best described by either (i) the monoexponential expressions (eqs 20 or 21), (ii) the involved integral expression (eq 18), or, in some cases, (iii) both the monoexponential and integral expressions provided a satisfactory description of the data. Cases (iii) correspond to situations where the relaxation time, τ, is rather short. Still, we have the means to discriminate between an absorption process that has a short (but nonzero value) relaxation time versus an infinitely fast surface adsorption at a short time via comparison between the involved integral fit and the monoexponential fit performed without including the initial 0-absorption point at *t* = 0 (which then provides a fitted value for  in case of eq 21). Our strategy, which includes proper accounting of transient processes, provides a robust means of characterizing the sorption kinetics.

The outcomes of the fittings discussed in detail below are given in Table 1; the fitted parameters for all data sets are given in Supporting Information Table S2, and the additional parameters evaluated from the involved integral fitting are collected in Table S3. In all cases, the mass transport coefficients in the polymer matrices are much lower than those in water, i.e., σ_p_ ≪ σ_w_, or, equivalently, σ ≈ σ_p_ (Table S3), which in turn implies τ_p_/τ_w_ ≫ 1 and legitimates the approximation τ = τ_p_ (cf. eq S4). Accordingly, diffusion in the particle body is overall rate limiting the absorption process, and the precise values estimated for *D*_X,w_ and δ_w_ are thus immaterial. The developed expressions defining the intraparticulate mass transfer coefficient of X, σ_p_ (eq S6), and the diffusion layer thickness, δ_p_ (eqs S7 and S8), show the interplay between *K*_w,p_ and *D*_X,p_ in defining the parameters that are retrieved from data fitting to eq 18. Thus, a proper interpretation of the kinetic features of each example requires consideration of all the involved parameters.

In the following, we discuss in detail the results for some selected examples, which illustrate the different effects various parameters have on the absorption kinetics. The complete set of curves for all absorption data considered is given in the Supporting Information.

**Figure 2:**
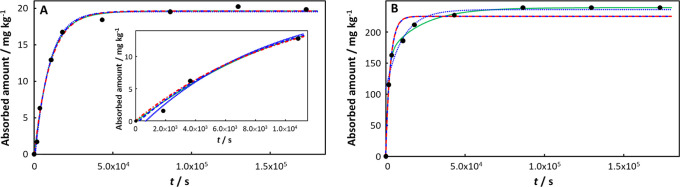
Absorption of Cd(II) on (A) pristine PS, with the inset showing data at short times, and (B) PS aged for 7 days in a mixture of H_2_O_2_ and Fe^2+^. Experimental data (black solid circles) from ref (40) for *c*_Cd,w_^*^ = 1.8 × 10^–5^ mol dm^–3^ (2 ppm). Computed curves correspond to the involved integral fit, eq 18 (including the initial 0-absorption point: green solid curve), the monoexponential, eq 21 (including the initial 0-absorption point: blue dashed line; excluding the initial 0-absorption point: blue dotted line), and the monoexponential, eq 20 (including the initial 0-absorption point: red dot-dashed line). See text for details of the fitting procedures.

In the case of Cd(II) absorption by PS^40^ the data for pristine PS are well described by both the monoexponential and involved integral expressions (Figure 2A), while only the involved integral expression provides a satisfactory description of the absorption by aged PS (Figure 2B). Upon aging, *K*_w,p_ for the Cd(II)-PS system increases from 9.8 in the pristine case to 119.6 for PS aged for 7 days in a mixture of H_2_O_2_ and Fe^2+^. Concomitantly, the diffusion coefficient of Cd(II) in the PS particle decreased by an order of magnitude from 3.41 × 10^–19^ m^2^ s^–1^ in the pristine case to 3.33 × 10^–20^ m^2^ s^–1^ in H_2_O_2_/Fe^2+^ -aged PS (Table 1). In the Cd(II)-PS example, the diffusion layer thickness in the particle, δ_p_, decreases by an order of magnitude upon aging (from 4.5 × 10^–8^ m to 7.07 × 10^–9^ m; Table S2 in Supporting Information), which in combination with the increased *K*_w,p_ results in a ca. order of magnitude increase in the normalized initial transient flux *J*_0_^+^/(*c*_X,w_^*^σ) (from 1.12 to 9.50; Table 1), and thus a decrease in the relaxation time τ from 5943 s in the pristine case to 1501 s for H_2_O_2_/Fe^2+^ aged PS. The rate constant *k*_r_ for Cd(II)-release slightly decreased upon aging (7.58 × 10^–5^ s^–1^ (6.55 day^–1^) for pristine and 4.71 × 10^–5^ s^–1^ (4.07 day^–1^) for H_2_O_2_/Fe^2+^ aged PS), while the rate constant for uptake *k*_u_ increases by almost an order of magnitude (from 7.43 × 10^–4^ s^–1^ (64.20 day^–1^) for pristine to 5.6 × 10^–3^ s^–1^ (483.84 day^–1^) for H_2_O_2_/Fe^2+^ aged PS).

**Figure 3:**
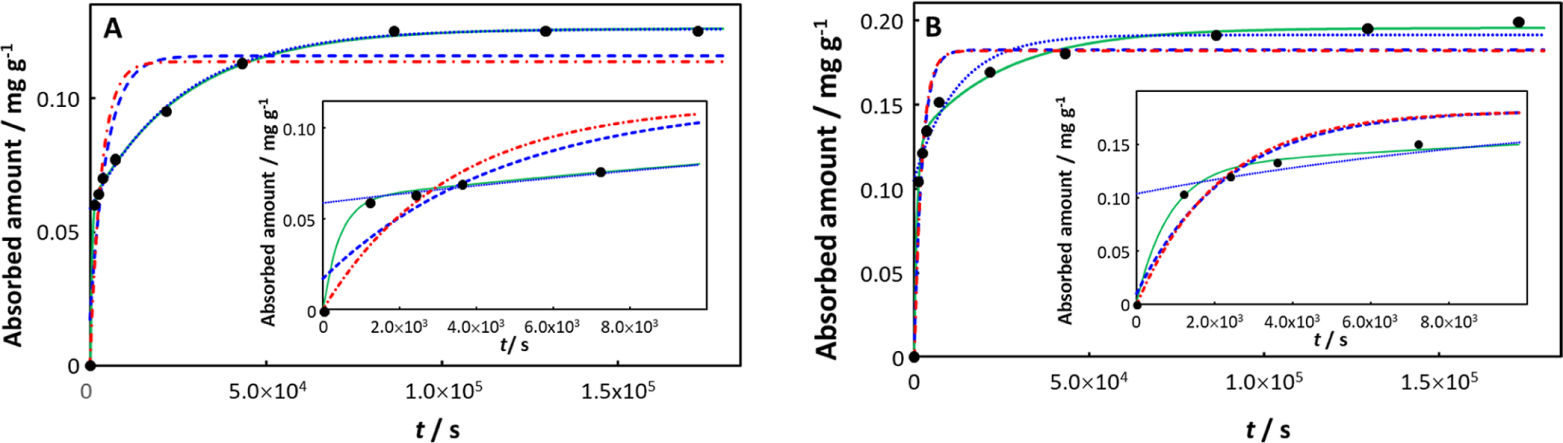
Absorption of tetracycline on (A) pristine PS and (B) aged PS, each with the inset showing data at short times. Experimental data (black solid circles) from ref (48). Computed curves correspond to the involved integral fit, eq 18 (including the initial 0-absorption point: green solid curve), the monoexponential, eq 21 (including the initial 0-absorption point: blue dashed line; excluding the initial 0-absorption point: blue dotted line) and the monoexponential, eq 20 (including the initial 0-absorption point: red dot-dashed line). See text for details of the fitting procedures.

In the case of tetracycline absorption by PS,^48^ only the involved integral expression provides a satisfactory description of the data for both pristine and aged PS (30 days of UV irradiation), Figure 3. The absorption curves for both pristine and aged PS exhibit a very rapid increase at short time. Scrutiny of the computed curves at short time (see Figure 3 insets) and comparison of the NRMSE values for the various fitting modes clearly shows that the involved integral fit with a short relaxation time (of the order of 10 min) provides the best description of the data. That is, the option of an initial infinitely fast surface adsorption process followed by a monoexponential steady-state absorption process does not adequately describe the experimental data. In this example, *K*_w,p_ increases somewhat upon aging from 6.3 for pristine PS to 9.8 for aged PS, *D*_X,p_ increases by a factor of ca. 2 (1.60 × 10^–16^ m^2^ s^–1^ in pristine, 3.64 × 10^–16^ m^2^ s^–1^ in aged PS), δ_p_ increases by a factor of ca. 2 (2.60 × 10^–7^ m for pristine, 5.51 × 10^–7^ m for aged PS), and the transient mass transport coefficient defined by *J*_0_^+^/*c*_X,w_^*^ remains approximately the same (1.20 × 10^–7^ m s^–1^ for pristine PS, 1.28 × 10^–7^ m s^–1^ for aged PS), with the net effect that upon aging both τ and *k*_u_ increase by a factor of ca. 2 (from 423 to 834 s, and 2.32 × 10^–4^ s^–1^ (20.04 day^–1^) to 3.88 × 10^–4^ s^–1^ (33.52 day^–1^), respectively).

**Figure 4:**
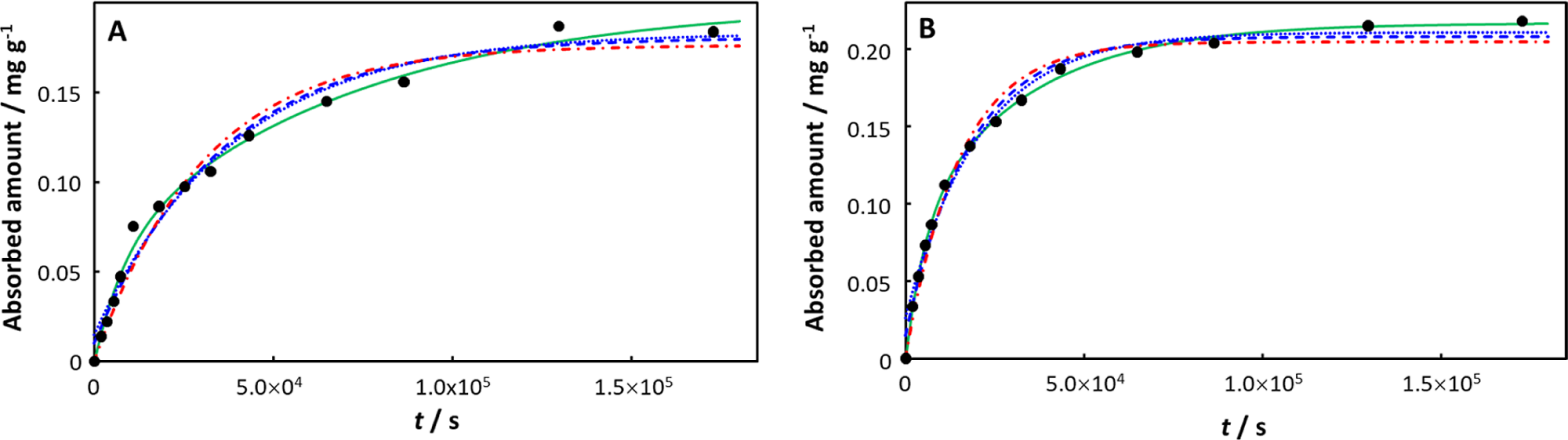
Absorption of Cd(II) on (A) pristine PS and (B) aged PS. Experimental data (black solid circles) are from ref (44). Computed curves correspond to the involved integral fit, eq 18 (including the initial 0-absorption point: green solid curve), the monoexponential, eq 21 (including the initial 0-absorption point: blue dashed line; excluding the initial 0-absorption point: blue dotted line), and the monoexponential, eq 20 (including the initial 0-absorption point: red dot-dashed line). See the text for details of the fitting procedures.

In some cases, superficial consideration might conclude that a monoexponential expression is sufficient to describe the data,^44,45^ but closer scrutiny reveals the importance of the transient contribution to the kinetics and thus the need to implement the involved integral equation. An example of this situation is provided by the kinetics of Cd(II) absorption by PS for both pristine and aged PS:^44^ the data are best described by the involved integral fit (eq 18), which captures the experimental points over the entire time range considered (Figure 4), including the equilibrium part (when reached) of the kinetic curve, and provides the lowest NRMSE values (Table 1). Indeed, the first few experimental data points lie within the transient regime (τ = 7799 and 4788 s for the pristine and aged PS, respectively). In this example, *K*_w,p_ increases only slightly upon aging (from 200.6 to 216.8) while *D*_X,p_ increases 3-fold (from 1.00 × 10^–15^ to 3.01 × 10^–15^ m^2^ s^–1^) and δ_p_ increases slightly (from 2.79 × 10^–6^ to 3.79 × 10^–6^ m), with the net outcome that the magnitude of the transient mass transport coefficient *J*_0_^+^/*c*_X,w_^*^ increases by a factor of ca. 2 upon aging (from 2.25 × 10^–7^ to 4.78 × 10^–7^ m s^–1^) resulting in a shorter relaxation time for sorption on the aged PS and a somewhat greater *k*_r_ for aged PS at 3.16 × 10^–5^ s^–1^ (2.7 day^–1^) cf. 1.43 × 10^–5^ s^–1^ (1.2 day^–1^) for pristine.

**Figure 5:**
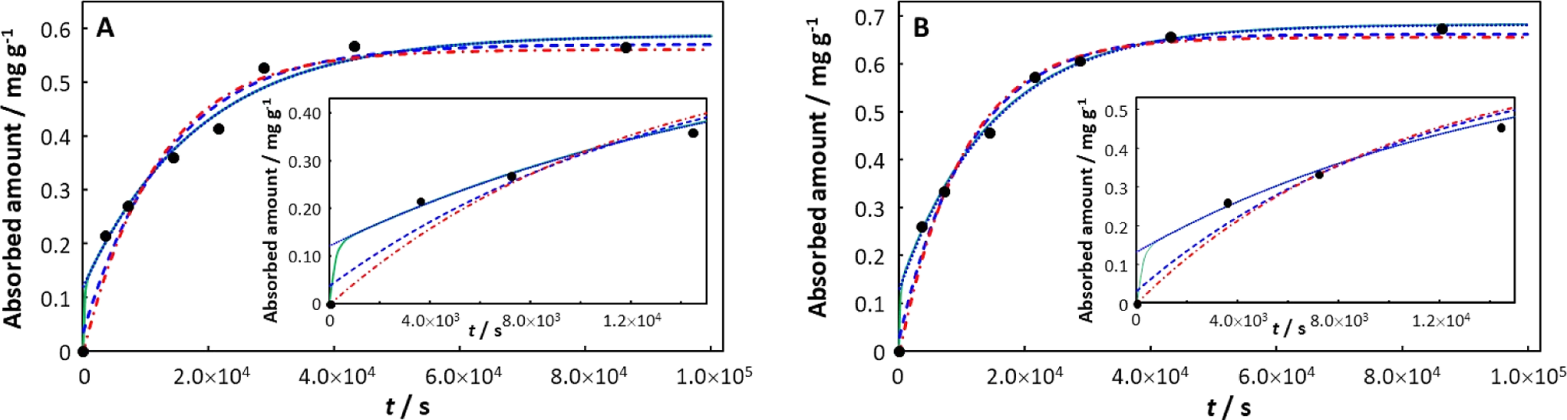
Absorption of atrazine by (A) pristine PS and (B) aged PS, each with an inset showing data at short times. Experimental data (black solid circles) from ref (42). Computed curves correspond to the involved integral fit, eq 18 (including the initial 0-absorption point: green solid curve), the monoexponential, eq 21 (including the initial 0-absorption point: blue dashed line; excluding the initial 0-absorption point: blue dotted line), and the monoexponential, eq 20 (including the initial 0-absorption point: red dot-dashed line). See the text for details of the fitting procedures.

Another example, which evidences the transient contribution, is the absorption of atrazine by PS;^42^Figure 5. In this case, the NRMSE for the involved integral fits is significantly lower than those obtained with the use of the monoexponential expressions, even though data scattering prevents the definitive exclusion of the latter fits. In particular, the data at short times, i.e., when the transient is operative, are best captured by the involved integral (see insets in Figure 5). Furthermore, *D*_X,p_ values in the range 6.97 × 10^–17^ to 2.51 × 10^–16^ m^2^ s^–1^ derived from analysis of the measured absorption data for pristine PS (for particle radius in the range 3.30 × 10^–5^ to 6.25 × 10^–5^ m)^42^ are in excellent agreement with an independently measured value of 1.51 × 10^–16^ m^2^ s^–1^.^49^ This result underscores the robustness and physicochemical consistency of the involved integral fitting (see also the comments below).

**Figure 6:**
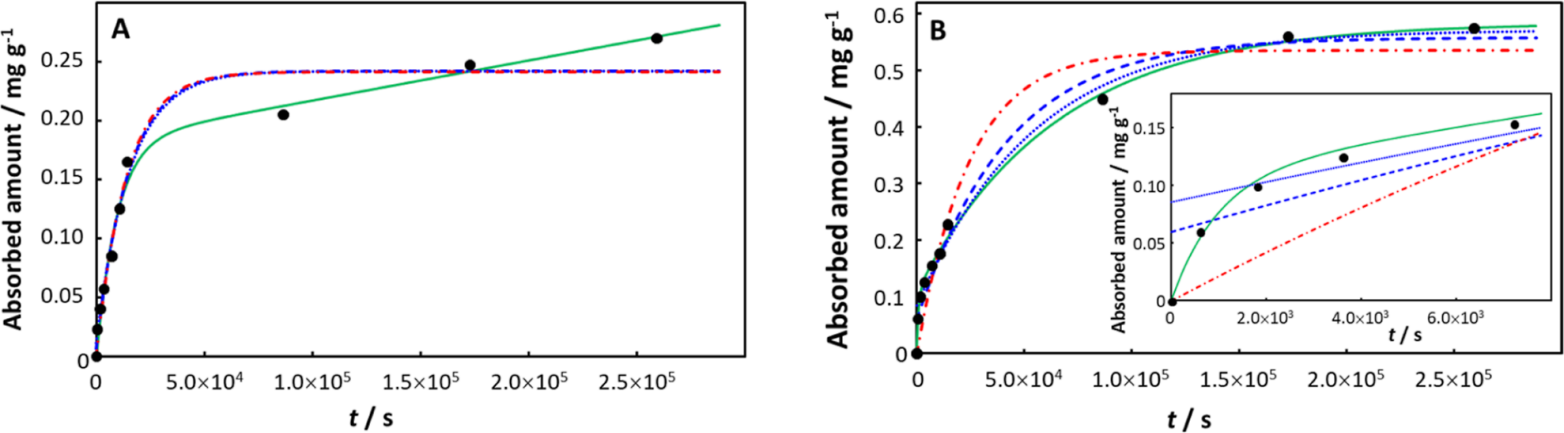
Absorption of sulfamethoxazole by (A) pristine PP and (B) aged PP, with the inset showing data at a short time. Experimental data (black solid circles) from ref (47). Computed curves correspond to the involved integral fit, eq 18 (including the initial 0-absorption point: green solid curve), the monoexponential, eq 21 (including the initial 0-absorption point: blue dashed line; excluding the initial 0-absorption point: blue dotted line), and the monoexponential, eq 20 (including the initial 0-absorption point: red dot-dashed line). See the text for details of the fitting procedures.

In some cases, a very long time is required to achieve equilibrium, and ongoing absorption at long times prevents observation of a true plateau within the experimental time frame. This situation is illustrated by the absorption kinetics of sulfamethoxazole in pristine PP.^47^Figure 6A shows that only the involved integral fit is able to describe the full curve, including the ongoing absorption at times greater than ca. 4 h. The obtained low values of *k*_r_ (4.34 × 10^–11^ s^–1^) and *D*_X,p_ (6.21 × 10^–26^ m^2^ s^–1^), together with the long relaxation time (τ = 9414 s), are all consistent with the observed features. The kinetics are rather different for the absorption of sulfamethoxazole by aged PP (Figure 6B). The transient contribution at short times can again only be captured by the involved integral fit, and the derived parameters show that the faster absorption kinetics of sulfamethoxazole by aged PP are largely due to the ca. 10 orders of magnitude increase in *D*_X,p_ (to 8.21 × 10^–16^ m^2^ s^–1^) together with a ca. 4 orders of magnitude decrease in *K*_w,p_ (from 7.84 × 10^5^ to 58.5). Upon aging, the relaxation time decreases by ca. an order of magnitude (to 982 s), and *k*_r_ becomes 6 orders of magnitude greater (1.54 × 10^–5^ s^–1^).

A similar shift in kinetic features upon aging is observed for the absorption of sulfamethazine by PP,^47^ (Figure S1). Again, only the integral fit is able to describe the experimental data over the entire time range considered. As compared to sulfamethoxazole, similar, albeit less dramatic, changes in the various parameters were found for sulfamethazine, i.e., upon aging, *D*_X,p_ increased by ca. 2 orders of magnitude (from 7.28 × 10^–16^ to 4.86 × 10^–14^ m^2^ s^–1^), the relaxation time decreased from 1898 to 1204 s, and *k*_r_ increased by an order of magnitude (from 1.05 × 10^–5^ to 1.07 × 10^–4^ s^–1^).

In general, for the cases that exhibit an initial rapid increase in absorption at short time, the goodness of fit using the involved integral formulation of the absorption kinetics (eq 18) with a short relaxation time τ is superior to that for any of the monoexponential fit modes (eqs 20 and 21); Table S2 in Supporting Information.

### Overall Trends in Kinetic Features

**Figure 7:**
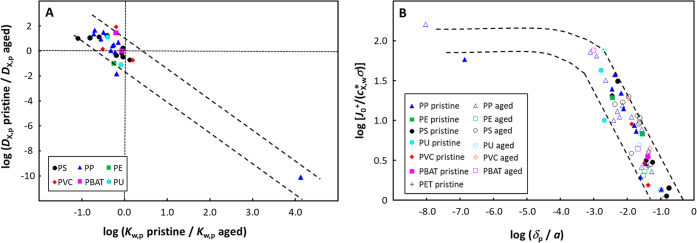
(A) Relative change in *K*_w,p_ and *D*_X,p_ upon the aging of plastic particles. (B) Plot of the dimensionless transient flux term, *J*_0_^+^/(*c*_X,w_^*^σ), versus the magnitude of the intraparticulate diffusion layer thickness relative to the particle radius, δ_p_/*a*. Dashed lines are guides to the eye. In both (A,B), parameters are obtained by involved integral fitting (eq 18, including the 0-absorption point at *t* = 0) of published experimental sorption curves.^40−48^

The complex interplay between the various parameters that govern the processes that contribute to the overall absorption kinetics confounds the construction of master curves that globally describe the interrelated parameters across all data sets considered herein. For a given combination of polymer type and target chemical, the effect of aging on the absorption kinetics will depend on the aging process, the polymer formulation, as well as the conditions in the aqueous medium, e.g., pH, electrolyte concentration, and composition. Nevertheless, the interconnection between the changes in *K*_w,p_ and *D*_X,p_ upon aging is rather consistent across all data, comprising the absorption of metal ions and a range of organic compounds by different polymer types (Figure 7A). Figure 7A suggests that the changes in *K*_w,p_ and *D*_X,p_ upon polymer aging are correlated by a power-law relationship of the form (*D*_X,p_ pristine)/(*D*_X,p_ aged) = ε{(*K*_w,p_ pristine)/(*K*_w,p_ aged)}^β^. However, it is not straightforward to a priori identify the physical origin of such a nonlinear connection between quantities that refer to essentially dynamic and equilibrium processes, i.e., diffusion (*D*_X,p_) and chemical partitioning equilibria of components at an interface (*K*_w,p_), respectively. As a hint to the underlying connections, we mention that complex nonlinear expressions combining dynamic and equilibrium quantities can be found in the literature on advanced physical modeling.^50,51^ For example, Lifson and Jackson^51^ established an expression for the diffusion coefficient of an inertial particle in a fixed static energy field; we recall that the Gibbs free energy of contaminant adsorption on a surface can be estimated in our case from the relevant thermodynamic constant, *K*_w,p_.

In most cases, *K*_w,p_ increases upon aging, whereas both increases and decreases in *D*_X,p_ occur upon aging. The increase in *K*_w,p_ can be ascribed to changes in the functional groups on the polymer backbone, e.g., photooxidation results in an increased proportion of carbonyl groups^52^ and conjugated π bonds,^53^ which form stronger interactions with the target compounds as compared to the pristine polymer. A decrease in *D*_X,p_ upon aging could be caused by decreases in the pore size and/or pore volume in the polymer matrix^46,47,54^ as well as by preferential degradation of the amorphous polymer content, and thus an increased degree of crystallinity in the aged polymer. For example, in the case of polystyrene, UV aging for 30 days resulted in an increase in the degree of crystallinity from 12 to 23%.^48^ In PET, the diffusion coefficient for water decreased from ca. 6 × 10^–13^ m^2^ s^–1^ at 10% crystallinity (by volume) to ca. 4 × 10^–13^ m^2^ s^–1^ at 30% crystallinity.^55^ In PET, the diffusion coefficients at *T* = 23 °C for 1-octanol and 1-butanol decreased by a factor of *ca*. 2 as the degree of crystallinity increased from 37 to 44%.^56^ Along this line of reasoning, an increase in *D*_X,p_ upon aging may be due to an increase in the pore size and/or pore volume in the polymer matrix.^42,54^

In several of the cases that can only be described by the involved integral fitting (eq 18), there is an ongoing increase in the extent of absorption over long periods of time (e.g., Figure 6A).^41,45,46^ This gradual, ongoing increase in absorption is often ignored in the literature, and “equilibrium isotherms” are recorded at an arbitrary time point, thereby resulting in *K*_w,p_ values that (grossly) underestimate the true equilibrium situation.^41,45,46^ Our approach provides a robust value for *c*_X,p_^tot,∞^ which can be used to determine the true *K*_w,p_ via eq 19. A comparison of these true *K*_w,p_ values with those determined from the slope of the experimental Henry isotherm data reveals the cases where the experimental equilibration time adopted for measurements of the isotherms was clearly insufficient (Table S4 in Supporting Information).

The ability to compute the diffusion coefficient of the target compound within the polymer matrix (eq S8) is a very useful feature of our computational strategy. While there are few literature data on independently determined *D*_X,p_ values for the target compounds considered herein,^32,33^ as noted above, the literature value for atrazine in pristine PS of 1.51 × 10^–16^ m^2^ s^–1^^49^ is in very good agreement with values derived herein of 6.97 × 10^–17^ to 2.51 × 10^–16^ m^2^ s^–1^. Furthermore, the *D*_X,p_ values derived herein are of a similar order of magnitude as those reported for a range of metal ions and organic compounds in various polymers.^32,33^ Although the computed *D*_X,p_ depends on the magnitude of the particle radius, which is in some cases somewhat distributed or poorly documented, the derived *D*_X,p_ is not very sensitive to the particle size within the ranges reported (Table S5 in Supporting Information).

**Figure 8:**
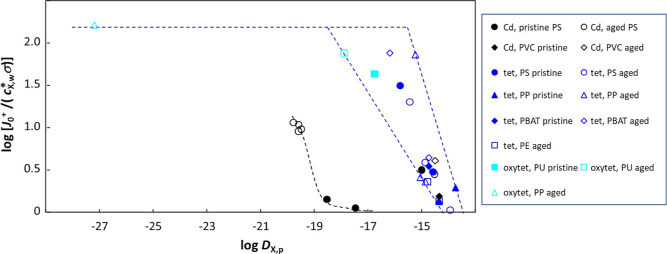
Relationship between log of the dimensionless transient flux term, *J*_0_^+^/(*c*_X,w_^*^σ), and log *D*_X,p_ for Cd(II), tetracycline (“tet”), and oxytetracycline (“oxytet”) absorption by plastic particles of various polymer types. Dashed lines are guides to the eye. Parameters obtained by involved integral fitting (eq 18, including the 0-absorption point at *t* = 0) of published experimental sorption curves.^40,41,44,46−48^

Our strategy provides a robust determination of the mass transport parameters, and overall, the results reveal the general importance of the transient and intraparticulate diffusion limitations in defining the kinetics of contaminant absorption by particulate micro/nanoplastics. This is illustrated by a plot of the dimensionless transient flux term, *J*_0_^+^/(*c*_X,w_^*^σ), versus the magnitude of the intraparticulate diffusion layer relative to the particle size, δ_p_/*a*, for all data that were described by the involved integral fitting (Figure 7B). The lower is the δ_p_/*a*, the higher the transient term, with indications that a limiting effect is observed at very low δ_p_/*a*, which corresponds to very low *D*_X,p_ for the data shown in Figure 7B. Across all data, the dimensionless transient flux term *J*_0_^+^/(*c*_X,w_^*^σ) generally decreases with increasing *D*_X,p_, albeit with some scatter in the plot due to the mitigating effects of concomitant changes in *K*_w,p_ (Figure S2 in Supporting Information). Useful insights are obtained by considering a subset of data for common target analytes. Grouping the results for Cd(II)^40,44^ and (oxy)tetracycline^41,46−48^ (Figure 8) reveals a threshold value for *D*_X,p_ with respect to its impact on the transient flux (cf. comment above regarding Figure 7B). Specifically, for Cd(II) the dimensionless transient flux *J*_0_^+^/(*c*_X,w_^*^σ) is low and approximately constant for *D*_X,p_ values greater than ca. 10^–19^ m^2^ s^–1^; then, the transient term increases with decreasing *D*_X,p_, while for (oxy)tetracycline the threshold lies at ca. 10^–15^ m^2^ s^–1^. Furthermore, for this subset of data, the change in the transient flux terms (γ, eq 12 and *J*_0_^+^/(*c*_X,w_^*^σ)) upon aging is largely driven by the change in *D*_X,p_ (Figure S3 in Supporting Information). The integral approach presented herein does not explicitly formulate a quantitative connection between the initial transient flux *J*_0_^+^ and *D*_X,p_, but rather accounts for relaxation of diffusive transport from transient to steady-state via the first term on the right-hand side of eq 10 and the introduction of the time constant τ. Thus, the observed increase in the dimensionless form of *J*_0_^+^ with decreasing δ_p_/*a* or decreasing *D*_X,p_ is consistent with the transient nature of *J*_0_^+^, i.e., the initial nonequilibrium diffusion of compounds from the bulk solution to the particle/solution interface. At very low δ_p_/*a* or *D*_X,p_, our data fitting exercise suggests that *J*_0_^+^ attains a plateau value, albeit that few experimental data correspond to extreme values (Figures 7B and 8). In reality, it might be expected that the transient flux would continue to increase with, e.g., decreasing *D*_X,p_, and thus that the observed plateau in the dimensionless form of *J*_0_^+^ may rather represent practical limitations in the (measurable) time required to detect changes in the amount of compound accumulated in the particles. Further experimental evidence is needed to comprehensively document the dependence of *J*_0_^+^ on δ_p_/*a* and *D*_X,p_ over a wide range of values.

By taking into account key elements of the flux of target compounds in the aqueous and polymer phases, our theory provides a flux-based identification of conditions under which the 2-compartment model (eqs 20 and 21) is applicable for any time point. The approach is generically applicable to any type of particle, with proper accounting for the involved geometry in the flux expressions and the particle surface-to-volume ratio involved in the expression of the kinetic constant for compound release (eq 13). In the long term, degradation will eventually lead to a temporal decrease in particle dimensions, which in itself results in faster uptake/release kinetics (eqs 16 and 17).^11,57^ Thus, faster degradation of the polymer matrix, e.g., as inherent by design in biobased/biodegradable plastics, may in fact pose a greater environmental risk in the context of the bioavailability of associated compounds. Nevertheless, as illustrated herein, changes in factors other than the mere particle size play a role in mediating the uptake/release kinetics, e.g., changes in the diffusion coefficient in the polymer phase and/or the equilibrium partitioning coefficient.

There are observations that aged plastics are ingested more frequently than pristine ones;^58,59^ however, there is disparity in the literature on the relative bioavailability of the associated compounds^60,61^ and the ensuing relative toxicity of pristine versus aged plastics.^62−64^ Establishment of scientifically sound environmental risk assessment of micro- and nanoplastics requires a mechanistic approach that couples the dynamic nature of particle-based processes—as detailed herein—with those occurring within biota, including local exposure conditions, gut transit times, membrane permeability, and translocation rates.

## Supporting Information Available

Additional elaboration of the involved integral fitting strategy and exploitation of the fitted parameters (eqs S1−S9), table summarizing the experimental conditions employed in the analyzed data sets (Table S1), table of collated fitted and derived parameters from data analysis according to the involved integral and mono-exponential expressions (Table S2), table of additional parameters obtained for the involved integral fitting (Table S3), table of equilibrium water/polymer partition coefficient values, *K*_w,p_ (Table S4), table of values adopted for *D*_X,p_ and particle radius, *a*, and sensitivity of *D*_X,p_ to *a* (Table S5), figure showing absorption kinetics of sulfamethazine on pristine and aged PP (Figure S1), figure showing the dimensionless transient flux *J*_0_^+^/(*c*_X,w_^*^σ) as a function of log of the diffusion coefficient in the polymer, *D*_X,p_ (Figure S2), log−log plot of change in *D*_X,p_ versus change in γ upon plastic particle aging and change in *D*_X,p_ versus change in the dimensionless transient flux term *J*_0_^+^/(*c*_X,w_^*^σ) upon plastic particle aging (Figure S3), figures showing the absorption kinetics of all systems reported in Table S2 that are not otherwise given in the manuscript (Figures S4−S27) (PDF)
