# Supplementary material for: Effect of Polymer Aging on Uptake/Release Kinetics of Metal Ions and Organic Molecules by Micro- and Nanoplastics: Implications for the Bioavailability of the Associated Compounds
Source: Environ Sci Technol. 2023 Oct 19;57(43):16552–63. doi: 10.1021/acs.est.3c05148 (PMC10620988; doi:10.1021/acs.est.3c05148)
Supplement: Supplementary file 1 — es3c05148_si_001.pdf [file es3c05148_si_001.pdf]

Supplementary Information

for the manuscript

Effect of Polymer Aging on Uptake/Release Kinetics of Metal Ions and Organic Molecules by Micro- and Nanoplastics: Implications for the Bioavailability of the Associated Compounds

Raewyn M. Town[1]*, Herman P. van Leeuwen[1,2], Jérôme F.L. Duval[3]*

[1]ECOSPHERE, Department of Biology, Universiteit Antwerpen, Groenenborgerlaan 171, 2020 Antwerpen, Belgium. Corresponding author, e-mail: raewyn.town@uantwerpen.be

[2]Physical Chemistry and Soft Matter, Wageningen University & Research, Stippeneng 4, 6708 WE Wageningen, The Netherlands

[3]Université de Lorraine, CNRS, LIEC, F-54000 Nancy, France. Corresponding author, e-mail: jerome.duval@univ-lorraine.fr

This document contains 54 pages, 9 equations, 5 tables, and 27 figures.

Specifically, the contents comprise additional elaboration of the involved integral fitting strategy and exploitation of the fitted parameters (**eqs S1-S9**,pp S2-S3), a Table summarizing the experimental conditions employed in the analysed data sets (**Table S1**, p. S4), a Table of collated fitted and derived parameters from data analysis according to the involved integral and mono-exponential expressions (**Table S2**, p. S6), a Table of additional parameters obtained for the involved integral fitting (**Table S3**, p. S13), a Table of equilibrium water/polymer partition coefficient values, $K_{w,p}$ (**Table S4**, p. S17), a Table of values adopted for $D_{X,w}$ and particle radius, a, and sensitivity of $D_{X,p}$ to a (**Table S5**, p. S19), a Figure showing absorption kinetics of sulfamethazine on pristine and aged PP (**Figure S1**, p.S23), a Figure showing the dimensionless transient flux $J_0^+ / \left(c^*_{X,w}\sigma\right)$ as a function of log of the diffusion coefficient in the polymer, $D_{X,p}$ (**Figure S2**, p. S24), Log-log plot of change in $D_{X,p}$ versus change in γ upon plastic particle aging and change in $D_{X,p}$ versus change in the dimensionless transient flux term $J_0^+ / \left(c^*_{X,w}\sigma\right)$ upon plastic particle aging (**Figure S3**, p. S24), and Figures showing the absorption kinetics of all systems reported in Table S2 that are not otherwise given in the manuscript (**Figures S4-S27**, pp. S25-S53).

For ease of cross-referencing, the reference numbers for the experimental data sources are maintained the same as those in the main text; additional references that are cited only in the SI are numbered sequentially from S1.

Involved integral fitting strategy and exploitation of the fitted parameters

Fitting of the experimental data using the involved integral formulation of $c_{\mathrm{X,p}}^{\mathrm{tot}}(t)$, eq 18 (corresponding to inclusion of the transient contribution to the flux via the time constant τ), requires adjusting the parameters k_{r}, τ, γ and $c_{\mathrm{X,p}}^{\mathrm{tot},\infty}$. The procedure for derivation of the additional parameters $K_{\mathrm{w,p}}$, k_{u}, δ_{p}, σ_{p}, $D_{\mathrm{X,p}}$ and J_0^+ is elaborated below. The total relaxation time, τ, (eq S1) is the sum of the involved process in the aqueous phase, τ_{w} (eq S2) and the polymer phase, τ_{p} (eq S3). The data modelling results confirm that the inequality $\tau_{\mathrm{p}} >> \tau_{\mathrm{w}}$ always applies.

$$\tau = \tau_{\mathrm{p}} + \tau_{\mathrm{w}} \qquad \text{[s]} \qquad \text{(S1)}$$

where

$$\tau_{\mathrm{p}} = \delta_{\mathrm{p}}^2 / D_{\mathrm{X,p}} \qquad \text{[s]} \qquad \text{(S2)}$$

and

$$\tau_{\mathrm{w}} = \frac{\delta_{\mathrm{w}}^2}{D_{\mathrm{X,w}}\left(1+\frac{\delta_{\mathrm{w}}}{a}\right)^2} \qquad \text{[s]} \qquad \text{(S3)}$$

For all practical cases considered (see **Table 1** in the main text and **Tables S2 and S3**), we have $\tau_{\mathrm{p}} >> \tau_{\mathrm{w}}$, thus $\tau \approx \tau_{\mathrm{p}}$. The dimensionless partitioning coefficient $K_{\mathrm{w,p}}$ is computed from the ratio of the fitted $c_{\mathrm{X,p}}^{\mathrm{tot},\infty}$ (mass/mass units) and the known bulk aqueous concentration of X, converted to per mass units using the density of water (1 kg dm^{-3}). From the fitted value of k_{r}, k_{u} follows simply via $k_{\mathrm{u}} = k_{\mathrm{r}}K_{\mathrm{w,p}}$ (eq 15). In the practically relevant cases where $\tau \approx \tau_{\mathrm{p}}$, using eq S2, 7 and 9 the expressions for τ (eq S1) and k_{r} (eq 13) can be rewritten as:

$$\tau \approx \delta_{\mathrm{p}} K_{\mathrm{w,p}} / \sigma_{\mathrm{p}} \qquad \text{[s]} \qquad \text{(S4)}$$

and

$$k_{\mathrm{r}} = \frac{\alpha}{K_{\mathrm{w,p}}} \frac{\sigma_{\mathrm{p}}\sigma_{\mathrm{w}}}{\sigma_{\mathrm{p}}+\sigma_{\mathrm{w}}} \qquad [\mathrm{s^{-1}}] \qquad \text{(S5)}$$

where $\alpha = 3/a$. Knowing the particle radius a, $K_{\mathrm{w,p}}$ (obtained from fitting), k_{r} (obtained from fitting), and knowing σ_{w} (which implies that we know or have reasonable estimates of $D_{\mathrm{X,w}}$ and δ_{w}), eqs S4 and S5 for τ and k_{r} respectively (whose values are known from fitting) are solved to obtain δ_{p} and σ_{p}. The solutions are:

$$\sigma_{\mathrm{p}} = \sigma_{\mathrm{w}} \Big/ \left(3\frac{\sigma_{\mathrm{w}}}{K_{\mathrm{w,p}}k_{\mathrm{r}}a} - 1\right) \qquad [\mathrm{m\ s^{-1}}] \qquad \text{(S6)}$$

and

$$\delta_{\mathrm{p}} = \tau\sigma_{\mathrm{p}} / K_{\mathrm{w,p}} = \tau\sigma_{\mathrm{w}} K_{\mathrm{w,p}}^{-1} / \left(3\frac{\sigma_{\mathrm{w}}}{K_{\mathrm{w,p}} k_{\mathrm{r}} a} - 1 \right) \qquad [\mathrm{m\ s^{-1}}] \qquad \text{(S7)}$$

The obtained values of σ_{p} and δ_{p} can then be used to compute $D_{\mathrm{X,p}}$ via:

$$D_{\mathrm{X,p}} = \sigma_{\mathrm{p}}\delta_{\mathrm{p}} / K_{\mathrm{w,p}} \qquad [\mathrm{m^2\ s^{-1}}] \qquad \text{(S8)}$$

which simply derives from eq 7. The obtained $D_{\mathrm{X,p}}$ is used to back-check that $\tau_{\mathrm{p}} / \tau_{\mathrm{w}} >> 1$, i.e. that the approximation $\tau \approx \tau_{\mathrm{p}}$ is valid. Finally, from the values of γ (obtained from fitting) and σ (computed from eq 9), using eq 12 we derive J_0^+ via:

$$J_0^+ = (\gamma + 1) c_{\mathrm{X,w}}^* \sigma \qquad [\mathrm{mol\ m^{-2}\ s^{-1}}] \qquad \text{(S9)}$$

Table S1. Experimental conditions employed in the analysed data sets

Plastic type, source, and radius (*a*; see further details in Table S5) [a]	Aging process	Absorption experimental conditions	ref
PS ($a = 3\times10^{-7}$ m), Shanghai Aladdin Biochemical Technology Co.	H_2O_2: PS shaken for 7 days in 1.5%H_2O_2, at pH 4, 25°C.<br>Fenton: PS shaken for 7 days in a mixture of 1.5%H_2O_2 and 3 mM Fe^{2+} at pH 4, 25°C.	0.5 g plastic particles per dm^3, Cd concentrations of 0.5 2 mg dm^{-3}, pH 4, 0.01 mol dm^{-3} KNO_3, shaken on "vibrating machine" at 25°C.	40
PS ($a = 5.73\times10^{-5}$ m), PP ($a = 3.83\times10^{-5}$ m), PE ($a = 4.34\times10^{-5}$ m), PBAT ($a = 3.82\times10^{-5}$ m), Dongguan Tesulang Chemical Materials Co.	UV: radiated for 96 h using a 250 W high pressure mercury lamp with wavelength 365 nm, radiant intensity 20 W m^{-2}.<br>Kps: 12 days in 0.37 mol dm^{-3} $K_2S_2O_8$ at pH 7.	1 g plastic particles per dm^3, 10 mg dm^{-3} tetracycline, shaken at 150 rpm (oscillator) at 25°C, pH and electrolyte composition not stated.	41
PS ($a = 4.8\times10^{-5}$ m), PP ($a = 1.5\times10^{-4}$ m), and PE ($a = 8.5\times10^{-5}$ m), Dupont China Holding Co.; PP, Sinopec Group.	UV irradiation for 96 h using 4 × 15 W UVC bulbs, wavelength 254 nm at 25°C.	0.4 g plastic particles per dm^3, 5 mg dm^{-3} atrazine, shaken at 150 rpm, at 25°C, pH and electrolyte composition not stated.	42
PS ($a = 3.75\times10^{-5}$ m) and PVC ($a = 3.75\times10^{-5}$ m), Dongguan Jing Tian Raw Materials of Plastics Co.	UV irradiation for 96 h using 4 × 15 W UVC bulbs, wavelength 254 nm at 25°C.	0.4 g plastic particles per dm^3, 10 mg dm^{-3} ciprofloxacin, shaken at 150 rpm, at 25°C, pH and electrolyte composition not stated.	43
PS ($a = 7.5\times10^{-5}$ m) and PVC ($a = 7.5\times10^{-5}$ m), Shanghai Aladdin Biochemical Technology Co.	Aged in filtered (0.45 µm) natural seawater for 3 months with UVA 340 nm, 20 W irradiation.	1 g plastic particles per dm^3; 1 mg dm^{-3} Cd(II), shaken at 160 rpm at 25°C, pH and electrolyte composition not stated.	44
PE ($a = 1.13\times10^{-4}$ m), PET ($a = 1.06\times10^{-4}$ m), and PVC ($a = 8.05\times10^{-5}$ m), Guangzhou Fuqiao Plastic Technology Co.	Aged for 30 days in 0.1 mol dm^{-3} $K_2S_2O_8$ at pH 7 and 70°C.	0.4 g plastic particles per dm^3, 10 mg dm^{-3} carbamazepine, shaken at 150 rpm at 25°C, pH 5, electrolyte composition not stated.	45
PU ($a = 5\times10^{-5}$ m), Shenzhen plastic raw materials Co.; ground and sieved.	UV irradiation for 10 days at 254 nm, intensity 5 mW cm^{-2}.	3.33 g plastic particles per dm^3, 10 mg dm^{-3} Cu(II) or oxytetracycline, shaken at 100 rpm at 25°C, pH and electrolyte composition not stated.	46

**Plastic type, source, and radius (*a*; see further details in Table S5)** [a]	**Aging process**	**Absorption experimental conditions**	**ref**
PP (a = 1.775×10^{-4} m), source not stated; mechanical crushing of industrial PP.	Aged by sonication in 65 % w/w HNO_3 for 30 min then autoclaved at 80°C for 60 min.	0.5 g plastic particles per dm^3, 10 mg dm^{-3} of oxytetracycline, chloramphenicol, sulfamethoxazole, sulfamethazine, enrofloxacin, ciprofloxacin, ofloxacin, norfloxacin, sulfamerazine, sulfathiazole, or tetracycline. Shaken at 150 rpm at 25°C, pH and electrolyte composition not stated.	47
PS (a = 5×10^{-5} m), Dongguan Yineng Plastics Co., ground and sieved.	UV irradiation for 30 days with a boron germicidal lamp, wavelength 254 nm, power 30 W, intensity 107 µW m^{-3}.	6.67 g plastic particles per dm^3, 20 mg dm^{-3} tetracycline, rotated at 8 rpm at 25°C, pH 7, electrolyte composition not stated.	48

[a] in all cases the particles have (approximate) spherical geometry

Table S2. Fitted and derived parameters from data analysis according to the involved integral and mono-exponential expressions of $c_{X,p}^{tot}(t)$ (eqs 18 and 20-21, respectively)

System	Fit[a,b]	Mono-exponential				Involved integral[e]						
		k_r (s^{-1})	$K_{w,p}$	$c_{X,p}(t=0)$/ $c_{X,p}(eq)$ [c]	NRMSE[d]	k_r (s^{-1})	τ (s)	$K_{w,p}$	δ_p / a	$D_{X,p}$ ($m^2\ s^{-1}$)	$J_0^+ / (c_{X,w}^* \sigma)$	NRMSE[d]
PS pristine, 0.5 ppm Cd [40]	incl 0,0	9.66×10^{-5}	26.6	-0.03	0.021	5.54×10^{-5}	9576	26.5	1.77×10^{-1}	2.94×10^{-19}	1.41	0.019
	excl. 0,0	1.04×10^{-4}	26.6	-0.07	0.018	5.54×10^{-5}	9576	26.5	1.77×10^{-1}	2.94×10^{-19}	1.41	0.023
	incl 0 (eq 20)	9.62×10^{-5}	26.7	0.0	0.024							
PS aged 7 day H_2O_2, 0.5 ppm Cd [40]	incl 0,0	2.76×10^{-4}	63.5	0.01	0.054	2.77×10^{-5}	2182	67.6	2.02×10^{-2}	1.67×10^{-20}	11.4	0.005
	excl. 0,0	7.38×10^{-5}	66.0	0.39	0.065	2.77×10^{-5}	2182	67.5	2.02×10^{-2}	1.67×10^{-20}	11.4	0.008
	incl 0 (eq 20)	2.81×10^{-4}	63.5	0.0	0.055							
PS aged 7 day Fenton, 0.5 ppm Cd [40]	incl 0,0	3.59×10^{-4}	164.6	-2.5×10^{-9}	0.036	3.73×10^{-5}	1898	170.5	2.36×10^{-2}	2.64×10^{-20}	10.7	0.003
	excl. 0,0	1.41×10^{-4}	166.9	0.40	0.069	3.73×10^{-5}	1898	170.5	2.36×10^{-2}	2.64×10^{-20}	10.7	0.005
	incl 0 (eq 20)	3.59×10^{-4}	164.6	0.0	0.036							
PS pristine, 2 ppm Cd [40]	incl 0,0	1.02×10^{-4}	9.8	-0.03	0.031	7.58×10^{-5}	5943	9.8	1.50×10^{-1}	3.41×10^{-19}	1.12	0.029
	excl. 0,0	1.11×10^{-4}	9.8	-0.08	0.031	7.58×10^{-5}	5943	9.8	1.50×10^{-1}	3.41×10^{-19}	1.12	0.034
	incl 0 (eq 20)	9.80×10^{-5}	9.8	0.0	0.032							
PS aged 7 day H_2O_2, 2 ppm Cd [40]	incl 0,0	2.06×10^{-4}	36.1	0.06	0.074	3.86×10^{-5}	1739	38.6	2.24×10^{-2}	2.60×10^{-20}	8.95	0.014
	excl. 0,0	7.52×10^{-5}	37.9	0.38	0.060	3.86×10^{-5}	1739	38.6	2.24×10^{-2}	2.60×10^{-20}	8.95	0.025
	incl 0 (eq 20)	2.45×10^{-4}	35.8	0.0	0.076							
PS aged 7 day Fenton, 2 ppm Cd [40]	incl 0,0	3.45×10^{-4}	112.7	0.02	0.062	4.71×10^{-5}	1501	119.6	2.36×10^{-2}	3.33×10^{-20}	9.50	0.014
	excl. 0,0	9.45×10^{-5}	118.1	0.46	0.068	4.71×10^{-5}	1501	119.6	2.36×10^{-2}	3.33×10^{-20}	9.50	0.029
	incl 0 (eq 20)	3.55×10^{-4}	112.6	0.0	0.063							
PP pristine, 10 mg/L tetracycline [41]	incl 0,0	1.18×10^{-4}	47.4	9×10^{-3}	0.011	8.62×10^{-5}	3645	48.1	1.05×10^{-1}	4.42×10^{-15}	1.36	0.009
	excl. 0,0	1.17×10^{-4}	47.4	0.02	0.012	8.62×10^{-5}	3645	48.1	1.05×10^{-1}	4.42×10^{-15}	1.36	0.010
	incl 0 (eq 20)	1.20×10^{-4}	47.3	0.0	0.012							
PP aged-UV, 10 mg/L tetracycline [41]	incl 0,0	8.58×10^{-5}	64.7	0.05	0.025	6.84×10^{-5}	1180	66.7	2.69×10^{-2}	9.03×10^{-16}	2.58	0.013
	excl. 0,0	7.95×10^{-5}	65.3	0.08	0.020	6.84×10^{-5}	1180	66.7	2.69×10^{-2}	9.03×10^{-16}	2.58	0.015
	incl 0 (eq 20)	9.54×10^{-5}	63.8	0.0	0.034							
PP aged-Kps, 10 mg/L tetracycline [41]	incl 0,0	9.07×10^{-5}	79.6	0.04	0.028	5.35×10^{-5}	2993	85.1	5.35×10^{-2}	1.40×10^{-15}	2.28	0.015
	excl. 0,0	8.58×10^{-5}	80.2	0.06	0.027	5.35×10^{-5}	2993	85.1	5.35×10^{-2}	1.40×10^{-15}	2.28	0.016
	incl 0 (eq 20)	9.79×10^{-5}	78.8	0.0	0.032							

**System**	**Fit[a,b]**	**Mono-exponential**				**Involved integral[e]**						
		k_r (s^{-1})	$K_{w,p}$	$c_{X,p}(t=0)/c_{X,p}(eq)$ [c]	NRMSE[d]	k_r (s^{-1})	τ (s)	$K_{w,p}$	δ_p / a	$D_{X,p}$ (m^2 s^{-1})	$J_0^+ / (c^*_{X,w}\sigma)$	NRMSE[d]
PE pristine, 10 mg/L tetracycline [41]	incl 0,0	8.91×10^{-5}	38.4	3×10^{-3}	0.014							
	excl. 0,0	8.88×10^{-5}	38.4	5×10^{-3}	0.015							
	incl 0 (eq 20)	8.96×10^{-5}	38.8	0.0	0.014							
PE aged-UV, 10 mg/L tetracycline [41]	incl 0,0	9.99×10^{-5}	49.4	0.04	0.023	8.13×10^{-5}	1212	50.5	3.29×10^{-2}	1.68×10^{-15}	2.28	0.011
	excl. 0,0	9.42×10^{-5}	49.7	0.07	0.020	8.13×10^{-5}	1212	50.5	3.29×10^{-2}	1.68×10^{-15}	2.19	0.012
	incl 0 (eq 20)	1.08×10^{-4}	48.9	0.0	0.028							
PE aged-Kps, 10 mg/L tetracycline [41]	incl 0,0	9.50×10^{-5}	68.4	0.02	0.018	7.08×10^{-5}	4361	69.8	1.03×10^{-1}	4.58×10^{-15}	1.33	0.018
	excl. 0,0	9.28×10^{-5}	69.6	0.03	0.019	7.08×10^{-5}	4361	69.8	1.03×10^{-1}	4.58×10^{-15}	1.33	0.020
	incl 0 (eq 20)	9.81×10^{-5}	68.2	0.0	0.019							
PBAT pristine, 10 mg/L tetracycline [41]	incl 0,0	1.72×10^{-4}	77.4	0.07	0.046	9.21×10^{-5}	1343	80.4	4.14×10^{-2}	1.85×10^{-15}	3.48	0.022
	excl. 0,0	1.47×10^{-4}	78.2	0.14	0.044	9.21×10^{-5}	1343	80.4	4.14×10^{-2}	1.85×10^{-15}	3.48	0.027
	incl 0 (eq 20)	1.98×10^{-4}	76.6	0.0	0.052							
PBAT aged-UV, 10 mg/L tetracycline [41]	incl 0,0	2.53×10^{-4}	91.4	0.07	0.037	1.86×10^{-4}	333	92.7	2.07×10^{-2}	1.87×10^{-15}	4.39	0.021
	excl. 0,0	2.11×10^{-4}	92.2	0.16	0.030	1.86×10^{-4}	333	92.7	2.07×10^{-2}	1.87×10^{-15}	4.39	0.027
	incl 0 (eq 20)	2.87×10^{-4}	90.9	0.0	0.044							
PBAT aged-Kps, 10 mg/L tetracycline [41]	incl 0,0	1.69×10^{-4}	125.8	0.11	0.050	1.34×10^{-4}	22	127.6	9.95×10^{-4}	6.51×10^{-17}	75.7	0.022
	excl. 0,0	1.34×10^{-4}	127.6	0.22	0.031	1.34×10^{-4}	22	127.6	1.00×10^{-3}	6.57×10^{-17}	75.0	0.031
	incl 0 (eq 20)	2.14×10^{-4}	123.8	0.0	0.066							
PS pristine, 10 mg/L tetracycline [41]	incl 0,0	1.07×10^{-4}	72.5	0.04	0.029	4.27×10^{-5}	3997	79.8	5.69×10^{-2}	2.66×10^{-15}	2.98	0.018
	excl. 0,0	1.01×10^{-4}	73.0	0.06	0.029	4.27×10^{-5}	3997	79.8	5.69×10^{-2}	2.66×10^{-15}	2.98	0.021
	incl 0 (eq 20)	1.15×10^{-4}	72.0	0.0	0.033							
PS aged-UV, 10 mg/L tetracycline [41]	incl 0,0	1.01×10^{-4}	59.0	-4×10^{-3}	0.014	8.39×10^{-5}	4517	59.5	1.27×10^{-1}	1.16×10^{-14}	1.05	0.012
	excl. 0,0	1.02×10^{-4}	59.0	-7×10^{-3}	0.015	8.39×10^{-5}	4517	59.5	1.27×10^{-1}	1.16×10^{-14}	1.05	0.013
	incl 0 (eq 20)	1.01×10^{-4}	59.1	0.0	0.014							
PS aged-Kps, 10 mg/L tetracycline [41]	incl 0,0	1.05×10^{-4}	85.4	0.05	0.023	9.08×10^{-5}	443	86.8	1.34×10^{-2}	1.34×10^{-15}	3.86	0.008
	excl. 0,0	9.71×10^{-5}	86.1	0.09	0.015	9.08×10^{-5}	443	86.8	1.34×10^{-2}	1.34×10^{-15}	3.86	0.010
	incl 0 (eq 20)	1.16×10^{-4}	84.5	0.0	0.032							
PE pristine, 5 mg/L atrazine [42]	incl 0,0	7.69×10^{-5}	105.5	0.06	0.043	5.65×10^{-5}	192.9	109.3	3.65×10^{-3}	4.97×10^{-16}	19.2	0.015
	excl. 0,0	5.65×10^{-5}	109.3	0.20	0.026	5.65×10^{-5}	195.0	109.3	3.68×10^{-3}	5.02×10^{-16}	19.0	0.026
	incl 0 (eq 20)	8.62×10^{-5}	104.0	0.0	0.048							

**System**	**Fit**[a,b]	**Mono-exponential**				**Involved integral**[e]						
		k_r (s^{-1})	$K_{w,p}$	$c_{X,p}$(t=0)/ $c_{X,p}$(eq)[c]	NRMSE[d]	k_r (s^{-1})	τ (s)	$K_{w,p}$	δ_p / a	$D_{X,p}$ (m^2 s^{-1})	$J_0^+ / (c^*_{X,w}\sigma)$	NRMSE[d]
PE aged, 5 mg/L atrazine [42]	incl 0,0	7.56×10^{-5}	189.0	0.03	0.037	6.50×10^{-5}	1374.4	192.1	3.00×10^{-2}	5.30×10^{-15}	2.04	0.031
	excl. 0,0	6.60×10^{-5}	191.9	0.10	0.046	6.50×10^{-5}	1374.4	192.1	3.00×10^{-2}	5.30×10^{-15}	2.04	0.046
	incl 0 (eq 20)	7.95×10^{-5}	187.9	0.0	0.038							
PP pristine, 5 mg/L atrazine [42]	incl 0,0	8.44×10^{-5}	80.1	0.07	0.052	5.46×10^{-5}	194.9	84.1	3.56×10^{-3}	1.46×10^{-15}	24.7	0.014
	excl. 0,0	5.46×10^{-5}	84.1	0.26	0.026	5.46×10^{-5}	195.8	84.1	3.58×10^{-3}	1.47×10^{-15}	24.6	0.026
	incl 0 (eq 20)	9.76×10^{-5}	78.7	0.0	0.058							
PP aged, 5 mg/L atrazine [42]	incl 0,0	6.95×10^{-5}	136.9	0.03	0.032	6.06×10^{-5}	192.9	139.1	3.93×10^{-3}	2.60×10^{-15}	10.1	0.024
	excl. 0,0	6.06×10^{-5}	139.1	0.11	0.037	6.06×10^{-5}	191.2	139.1	3.90×10^{-3}	2.57×10^{-15}	10.2	0.037
	incl 0 (eq 20)	7.36×10^{-5}	136.0	0.0	0.035							
PS pristine, 5 mg/L atrazine [42]	incl 0,0	7.32×10^{-5}	114.2	0.06	0.056	5.45×10^{-5}	193.3	118.0	3.52×10^{-3}	1.48×10^{-16}	20.2	0.036
	excl. 0,0	5.45×10^{-5}	118.0	0.21	0.063	5.45×10^{-5}	193.7	118.0	3.52×10^{-3}	1.48×10^{-16}	20.1	0.063
	incl 0 (eq 20)	8.29×10^{-5}	112.4	0.0	0.062							
PS aged, 5 mg/L atrazine [42]	incl 0,0	9.02×10^{-5}	132.5	0.05	0.039	6.72×10^{-5}	190.8	136.6	4.29×10^{-3}	2.60×10^{-16}	15.9	0.016
	excl. 0,0	6.72×10^{-5}	136.6	0.19	0.028	6.72×10^{-5}	188.2	136.6	4.23×10^{-3}	2.56×10^{-16}	16.1	0.028
	incl 0 (eq 20)	9.82×10^{-5}	131.3	0.0	0.042							
PS pristine, 10 mg/L ciprofloxacin [43]	incl 0,0	3.71×10^{-5}	255.3	-0.04	0.060							
	excl. 0,0	4.13×10^{-5}	251.9	-0.09	0.062							
	incl 0 (eq 20)	3.45×10^{-5}	257.7	0.0	0.062							
PS aged, 10 mg/L ciprofloxacin [43]	incl 0,0	2.80×10^{-5}	539.9	0.20	0.091	1.18×10^{-5}	1824.3	626.1	7.28×10^{-3}	4.09×10^{-17}	16.9	0.022
	excl. 0,0	1.34×10^{-5}	610.9	0.34	0.042	1.18×10^{-5}	1824.3	626.1	7.28×10^{-3}	4.09×10^{-17}	16.9	0.036
	incl 0 (eq 20)	7.18×10^{-5}	486.7	0.0	0.109							
PVC pristine, 10 mg/L ciprofloxacin [43]	incl 0,0	3.64×10^{-5}	271.0	-0.02	0.050	2.20×10^{-6}	18641	558.6	1.37×10^{-2}	1.42×10^{-17}	8.94	0.043
	excl. 0,0	3.87×10^{-5}	268.3	-0.04	0.056	2.20×10^{-6}	18641	558.6	1.37×10^{-2}	1.42×10^{-17}	8.94	0.049
	incl 0 (eq 20)	3.49×10^{-5}	272.9	0.0	0.050							
PVC aged, 10 mg/L ciprofloxacin [43]	incl 0,0	1.79×10^{-4}	306.9	0.04	0.083	1.52×10^{-5}	2123.4	356.5	1.08×10^{-2}	7.77×10^{-17}	18.8	0.018
	excl. 0,0	2.17×10^{-5}	345.8	0.54	0.070	1.52×10^{-5}	2123.4	356.5	1.08×10^{-2}	7.77×10^{-17}	18.8	0.040
	incl 0 (eq 20)	1.90×10^{-4}	306.2	0.0	0.084							
PS pristine, 1 mg/L Cd [44]	incl 0,0	2.82×10^{-5}	181.1	0.06	0.046	1.43×10^{-5}	7798.5	200.6	3.72×10^{-2}	1.00×10^{-15}	3.13	0.026
	excl. 0,0	2.61×10^{-5}	183.3	0.08	0.047	1.43×10^{-5}	7798.5	200.6	3.72×10^{-2}	1.00×10^{-15}	3.13	0.029
	incl 0 (eq 20)	3.31×10^{-5}	176.6	0.0	0.052							

System	Fit[a,b]	Mono-exponential				Involved integral[e]						
		k_r (s^{-1})	$K_{w,p}$	$c_{X,p}(t=0)/c_{X,p}(eq)$ [c]	NRMSE[d]	k_r (s^{-1})	τ (s)	$K_{w,p}$	δ_p / a	$D_{X,p}$ ($m^2\ s^{-1}$)	$J_0^+ / (c^*_{X,w}\sigma)$	NRMSE[d]
PS aged, 1 mg/L Cd [44]	incl 0,0	5.63×10^{-5}	208.1	0.07	0.034	3.16×10^{-5}	4787.8	216.8	5.05×10^{-2}	3.01×10^{-15}	2.79	0.010
	excl. 0,0	4.91×10^{-5}	210.8	0.12	0.030	3.16×10^{-5}	4787.8	216.8	5.05×10^{-2}	3.01×10^{-15}	2.79	0.012
	incl 0 (eq 20)	6.62×10^{-5}	204.9	0.0	0.042							
PVC pristine, 1 mg/L Cd [44]	incl 0,0	6.22×10^{-5}	157.6	0.03	0.029	5.57×10^{-5}	2292.2	158.7	4.27×10^{-2}	4.47×10^{-15}	1.53	0.026
	excl. 0,0	5.91×10^{-5}	158.2	0.06	0.032	5.57×10^{-5}	2292.2	158.7	4.27×10^{-2}	4.47×10^{-15}	1.53	0.031
	incl 0 (eq 20)	6.60×10^{-5}	156.8	0.0	0.031							
PVC aged, 1 mg/L Cd [44]	incl 0,0	9.95×10^{-5}	485.6	0.08	0.045	3.89×10^{-5}	3331.0	511.5	4.35×10^{-2}	3.19×10^{-15}	4.04	0.011
	excl. 0,0	7.13×10^{-5}	496.9	0.21	0.040	3.89×10^{-5}	3331.0	511.5	4.35×10^{-2}	3.19×10^{-15}	4.04	0.015
	incl 0 (eq 20)	1.18×10^{-4}	480.0	0.0	0.051							
PVC pristine, 10 mg/L carbamazepine [45]	incl 0,0	2.14×10^{-5}	119.4	0.04	0.034	1.22×10^{-5}	8018.1	133.3	3.25×10^{-2}	8.56×10^{-16}	2.87	0.017
	excl. 0,0	1.98×10^{-5}	121.1	0.07	0.034	1.22×10^{-5}	8018.1	133.3	3.25×10^{-2}	8.56×10^{-16}	2.87	0.019
	incl 0 (eq 20)	2.46×10^{-5}	116.4	0.0	0.041							
PVC aged, 10 mg/L carbamazepine [45]	incl 0,0	7.82×10^{-5}	197.7	0.04	0.032	2.14×10^{-5}	6689.4	208.8	4.76×10^{-2}	1.04×10^{-17}	4.44	0.006
	excl. 0,0	6.96×10^{-5}	198.9	0.09	0.034	2.14×10^{-5}	6689.4	208.8	4.76×10^{-2}	1.04×10^{-17}	4.44	0.008
	incl 0 (eq 20)	8.44×10^{-5}	196.8	0.0	0.035							
PE pristine, 10 mg/L carbamazepine [45]	incl 0,0	3.60×10^{-5}	165.6	0.02	0.030	4.92×10^{-6}	17264	214.0	2.83×10^{-2}	5.94×10^{-16}	6.72	0.013
	excl. 0,0	3.48×10^{-5}	166.2	0.03	0.033	4.92×10^{-6}	17264	214.0	2.83×10^{-2}	5.94×10^{-16}	6.72	0.015
	incl 0 (eq 20)	3.75×10^{-5}	164.9	0.0	0.031							
PET pristine, 10 mg/L carbamazepine [45]	incl 0,0	3.26×10^{-5}	139.3	0.04	0.035	1.98×10^{-5}	6385.8	147.3	4.22×10^{-2}	3.15×10^{-15}	2.59	0.017
	excl. 0,0	3.02×10^{-5}	140.5	0.07	0.035	1.98×10^{-5}	6385.8	147.3	4.22×10^{-2}	3.13×10^{-15}	2.67	0.019
	incl 0 (eq 20)	3.65×10^{-5}	137.5	0.0	0.039							
PU pristine, 10 mg/L Cu [46]	incl 0,0	1.62×10^{-5}	28.4	0.03	0.036	1.51×10^{-5}	407.8	28.7	2.06×10^{-3}	2.58×10^{-17}	10.0	0.033
	excl. 0,0	1.51×10^{-5}	28.7	0.06	0.042	1.51×10^{-5}	418.4	28.7	2.10×10^{-3}	2.65×10^{-17}	9.77	0.042
	incl 0 (eq 20)	1.72×10^{-5}	28.2	0.0	0.038							
PU aged, 10 mg/L Cu [46]	incl 0,0	2.99×10^{-5}	33.4	0.09	0.065	1.73×10^{-5}	3978.2	34.9	2.30×10^{-2}	3.30×10^{-16}	4.93	0.037
	excl. 0,0	1.95×10^{-5}	34.6	0.25	0.060	1.73×10^{-5}	3978.2	34.9	2.30×10^{-2}	3.30×10^{-16}	4.93	0.057
	incl 0 (eq 20)	3.60×10^{-5}	32.9	0.0	0.073							
PU pristine, 10 mg/L oxytetracycline [46]	incl 0,0	1.87×10^{-5}	28.2	0.09	0.057	1.27×10^{-5}	389.8	29.7	1.65×10^{-3}	1.76×10^{-17}	42.4	0.027
	excl. 0,0	1.27×10^{-5}	29.7	0.21	0.040	1.27×10^{-5}	379.6	29.7	1.61×10^{-3}	1.71×10^{-17}	43.5	0.040
	incl 0 (eq 20)	2.39×10^{-5}	27.4	0.0	0.070							

System	Fit[a,b]	Mono-exponential				Involved integral[e]						
		k_r (s^{-1})	$K_{w,p}$	$c_{X,p}(t=0)/c_{X,p}(eq)$ [c]	NRMSE[d]	k_r (s^{-1})	τ (s)	$K_{w,p}$	δ_p / a	$D_{X,p}$ ($m^2 s^{-1}$)	$J_0^+ / (c^*_{X,w}\sigma)$	NRMSE[d]
PU aged, 10 mg/L oxytetracycline [46]	incl 0,0	9.89×10^{-5}	37.6	0.01	0.043	7.82×10^{-7}	7704.1	74.3	2.00×10^{-3}	1.31×10^{-18}	75.3	0.007
	excl. 0,0	5.34×10^{-5}	38.7	0.36	0.070	7.82×10^{-7}	7704.1	74.3	2.00×10^{-3}	1.31×10^{-18}	75.3	0.015
	incl 0 (eq 20)	1.01×10^{-4}	37.6	0.0	0.043							
PP pristine, 10 mg/L oxytetracycline [47]	incl 0,0	1.56×10^{-4}	126.6	-0.04	0.058							
	excl. 0,0	1.64×10^{-4}	126.4	-0.08	0.061							
	incl 0 (eq 20)	1.48×10^{-4}	126.9	0.0	0.061							
PP aged, 10 mg/L oxytetracycline [47]	incl 0,0	1.41×10^{-4}	219.5	0.05	0.062	6.68×10^{-12}	4192.2	3.96×10^{7}	9.32×10^{-9}	6.56×10^{-28}	161.0	0.028
	excl. 0,0	1.16×10^{-4}	221.3	0.16	0.083	6.93×10^{-12}	4192.2	3.82×10^{7}	9.69×10^{-9}	7.05×10^{-28}	161.0	0.042
	incl 0 (eq 20)	1.52×10^{-4}	218.4	0.0	0.064							
PP pristine, 10 mg/L chloramphenicol [47]	incl 0,0	6.83×10^{-5}	71.2	0.08	0.057	2.47×10^{-5}	2232.8	73.6	1.84×10^{-2}	4.77×10^{-15}	7.26	0.033
	excl. 0,0	5.97×10^{-5}	71.4	0.14	0.055	2.47×10^{-5}	2232.8	73.6	1.84×10^{-2}	4.77×10^{-15}	7.26	0.039
	incl 0 (eq 20)	8.17×10^{-5}	70.7	0.0	0.069							
PP aged, 10 mg/L chloramphenicol [47]	incl 0,0	4.95×10^{-5}	222.4	0.04	0.071	2.76×10^{-6}	7885.2	384.9	7.27×10^{-3}	2.11×10^{-16}	15.4	0.023
	excl. 0,0	4.44×10^{-5}	223.5	0.07	0.077	2.76×10^{-6}	7885.2	385.0	7.27×10^{-3}	2.11×10^{-16}	15.4	0.025
	incl 0 (eq 20)	5.58×10^{-5}	221.3	0.0	0.073							
PP pristine, 10 mg/L sulfamethoxazole [47]	incl 0,0	6.73×10^{-5}	24.2	0.03	0.059	4.34×10^{-11}	9413.7	7.84×10^{5}	1.36×10^{-7}	6.21×10^{-26}	58.1	0.033
	excl. 0,0	6.46×10^{-5}	24.2	0.05	0.067	4.33×10^{-11}	9413.7	7.85×10^{5}	1.36×10^{-7}	6.19×10^{-26}	58.1	0.038
	incl 0 (eq 20)	7.14×10^{-5}	24.1	0.0	0.061							
PP aged, 10 mg/L sulfamethoxazole [47]	incl 0,0	2.38×10^{-5}	55.8	0.11	0.049	1.54×10^{-5}	982.3	58.5	5.06×10^{-3}	8.21×10^{-16}	13.0	0.016
	excl. 0,0	1.85×10^{-5}	57.3	0.15	0.033	1.54×10^{-5}	982.3	58.5	5.08×10^{-3}	8.21×10^{-16}	13.0	0.019
	incl 0 (eq 20)	4.11×10^{-5}	53.6	0.0	0.071							
PP pristine, 10 mg/L sulfamethazine [47]	incl 0,0	7.17×10^{-5}	18.7	0.12	0.084	1.05×10^{-5}	1897.5	21.5	6.65×10^{-3}	7.28×10^{-16}	21.8	0.028
	excl. 0,0	5.65×10^{-5}	18.9	0.20	0.084	1.05×10^{-5}	1897.5	21.5	6.65×10^{-3}	7.28×10^{-16}	21.8	0.033
	incl 0 (eq 20)	9.39×10^{-5}	18.5	0.0	0.098							
PP aged, 10 mg/L sulfamethazine [47]	incl 0,0	2.71×10^{-4}	31.4	0.06	0.049	1.07×10^{-4}	1203.8	32.7	4.32×10^{-2}	4.86×10^{-14}	4.13	0.007
	excl. 0,0	1.99×10^{-4}	32.1	0.19	0.049	1.07×10^{-4}	1203.8	32.7	4.32×10^{-2}	4.86×10^{-14}	4.13	0.009
	incl 0 (eq 20)	3.11×10^{-4}	31.1	0.0	0.054							
PP pristine, 10 mg/L enrofloxacin [47]	incl 0,0	1.09×10^{-4}	50.8	0.03	0.071	2.78×10^{-6}	4599.7	77.3	4.27×10^{-3}	1.25×10^{-16}	37.3	0.033
	excl. 0,0	1.04×10^{-4}	50.9	0.05	0.077	2.78×10^{-6}	4599.7	77.3	4.28×10^{-3}	1.25×10^{-16}	37.3	0.036
	incl 0 (eq 20)	1.15×10^{-4}	50.7	0.0	0.072							

**System**	**Fit[a,b]**	**Mono-exponential**				**Involved integral[e]**						
		k_r (s^{-1})	$K_{w,p}$	$c_{X,p}(t=0)$/ $c_{X,p}(eq)$ [c]	NRMSE[d]	k_r (s^{-1})	τ (s)	$K_{w,p}$	δ_p / a	$D_{X,p}$ (m^2 s^{-1})	$J_0^+ / (c^*_{X,w}\sigma)$	NRMSE[d]
PP aged, 10 mg/L enrofloxacin [47]	incl 0,0	8.74×10^{-6}	134.6	0.16	0.094	4.88×10^{-6}	1366.3	161.6	2.24×10^{-3}	1.15×10^{-16}	31.7	0.053
	excl. 0,0	6.85×10^{-6}	144.3	0.18	0.083	4.88×10^{-6}	1366.3	161.6	2.24×10^{-3}	1.15×10^{-16}	31.7	0.063
	incl 0 (eq 20)	5.10×10^{-5}	102.8	0.0	0.133							
PP pristine, 10 mg/L ciprofloxacin [47]	incl 0,0	7.45×10^{-5}	76.1	-0.02	0.013							
	excl. 0,0	7.66×10^{-5}	76.0	-0.04	0.010							
	incl 0 (eq 20)	7.13×10^{-5}	76.1	0.0	0.019							
PP aged, 10 mg/L ciprofloxacin [47]	incl 0,0	1.22×10^{-4}	152.9	0.02	0.030							
	excl. 0,0	1.18×10^{-4}	153.1	0.04	0.035							
	incl 0 (eq 20)	1.27×10^{-4}	152.6	0.0	0.032							
PP pristine, 10 mg/L ofloxacin [47]	incl 0,0	1.44×10^{-4}	19.8	2×10^{-3}	0.040							
	excl. 0,0	1.44×10^{-4}	19.8	4×10^{-3}	0.046							
	incl 0 (eq 20)	1.45×10^{-4}	19.8	0.0	0.040							
PP aged, 10 mg/L ofloxacin [47]	incl 0,0	5.63×10^{-5}	63.4	0.03	0.050	7.59×10^{-6}	10675	71.5	2.71×10^{-2}	2.16×10^{-15}	7.75	0.036
	excl. 0,0	5.40×10^{-5}	63.4	0.04	0.056	7.59×10^{-6}	10675	71.5	2.71×10^{-2}	2.16×10^{-15}	7.75	0.042
	incl 0 (eq 20)	5.98×10^{-5}	63.2	0.0	0.051							
PP pristine, 10 mg/L norfloxacin [47]	incl 0,0	2.11×10^{-4}	12.3	0.06	0.041	1.54×10^{-4}	699.8	12.5	3.61×10^{-2}	5.84×10^{-14}	2.84	0.022
	excl. 0,0	1.79×10^{-4}	12.4	0.15	0.036	1.54×10^{-4}	699.8	12.5	3.61×10^{-2}	5.84×10^{-14}	2.84	0.029
	incl 0 (eq 20)	2.39×10^{-4}	12.2	0.0	0.047							
PP aged, 10 mg/L norfloxacin [47]	incl 0,0	6.12×10^{-5}	52.7	0.07	0.065	8.85×10^{-6}	4594	61.8	1.36×10^{-2}	1.26×10^{-15}	11.0	0.039
	excl. 0,0	5.40×10^{-5}	52.9	0.12	0.071	8.85×10^{-6}	4594	61.8	1.36×10^{-2}	1.26×10^{-15}	11.0	0.046
	incl 0 (eq 20)	7.18×10^{-5}	52.4	0.0	0.072							
PP pristine, 10 mg/L sulfamerazine [47]	incl 0,0	7.69×10^{-5}	31.5	0.10	0.070	2.89×10^{-5}	1693.1	32.3	1.64×10^{-2}	4.97×10^{-15}	8.67	0.020
	excl. 0,0	6.40×10^{-5}	31.7	0.18	0.066	2.89×10^{-5}	1693.1	32.3	1.64×10^{-2}	4.97×10^{-15}	8.67	0.023
	incl 0 (eq 20)	9.72×10^{-5}	31.2	0.0	0.085							
PP aged, 10 mg/L sulfamerazine [47]	incl 0,0	1.36×10^{-5}	107.3	0.11	0.057	9.26×10^{-6}	1886.7	115.7	5.86×10^{-3}	5.68×10^{-16}	11.0	0.029
	excl. 0,0	1.16×10^{-5}	110.5	0.13	0.047	9.26×10^{-6}	1886.7	115.7	5.86×10^{-3}	5.68×10^{-16}	11.0	0.033
	incl 0 (eq 20)	3.05×10^{-5}	96.1	0.0	0.086							
PP pristine, 10 mg/L sulfathiazole [47]	incl 0,0	8.38×10^{-5}	59.7	0.16	0.070	5.49×10^{-5}	420.9	60.2	7.77×10^{-3}	4.47×10^{-15}	14.0	0.027
	excl. 0,0	6.27×10^{-5}	60.1	0.27	0.044	5.49×10^{-5}	420.9	60.2	7.77×10^{-3}	4.47×10^{-15}	14.0	0.038
	incl 0 (eq 20)	1.20×10^{-4}	58.5	0.0	0.100							

System	Fit[a,b]	Mono-exponential				Involved integral[e]						
		k_r (s^{-1})	$K_{w,p}$	$c_{X,p}$(t=0)/ $c_{X,p}$(eq)[c]	NRMSE[d]	k_r (s^{-1})	τ (s)	$K_{w,p}$	δ_p / a	$D_{X,p}$ (m^2 s^{-1})	$J_0^+ / (c^*_{X,w}\sigma)$	NRMSE[d]
PP aged, 10 mg/L sulfathiazole [47]	incl 0,0	1.67×10^{-4}	113.4	0.12	0.060	1.36×10^{-4}	26.2	114.5	1.21×10^{-3}	1.75×10^{-15}	64.3	0.030
	excl. 0,0	1.36×10^{-4}	114.5	0.23	0.047	1.36×10^{-4}	25.2	114.5	1.16×10^{-3}	1.68×10^{-15}	66.8	0.047
	incl 0 (eq 21)	2.09×10^{-4}	111.7	0.0	0.077							
PP pristine, 10 mg/L tetracycline [47]	incl 0,0	7.68×10^{-5}	12.5	0.03	0.021	6.88×10^{-5}	1100.7	12.5	2.53×10^{-2}	1.82×10^{-14}	1.94	0.014
	excl. 0,0	7.43×10^{-5}	12.5	0.04	0.020	6.88×10^{-5}	1100.7	12.5	2.53×10^{-2}	1.82×10^{-14}	1.94	0.016
	incl 0 (eq 20)	8.07×10^{-5}	12.5	0.0	0.024							
PP aged, 10 mg/L tetracycline [47]	incl 0,0	8.09×10^{-5}	38.8	0.10	0.063	7.04×10^{-5}	33.4	38.9	7.87×10^{-4}	5.82×10^{-16}	72.2	0.048
	excl. 0,0	7.04×10^{-5}	38.9	0.17	0.065	7.04×10^{-5}	34.1	38.9	8.06×10^{-4}	5.94×10^{-16}	70.7	0.065
	incl 0 (eq 20)	9.79×10^{-5}	38.6	0.0	0.080							
PS pristine, 20 mg/L tetracycline [48]	incl 0,0	2.10×10^{-4}	5.8	0.15	0.105	3.69×10^{-5}	422.7	6.3	5.20×10^{-3}	1.60×10^{-16}	31.0	0.009
	excl. 0,0	3.86×10^{-5}	6.3	0.47	0.023	3.69×10^{-5}	422.7	6.3	5.20×10^{-3}	1.60×10^{-16}	31.0	0.018
	incl 0 (eq 20)	3.05×10^{-4}	5.7	0.0	0.113							
PS aged, 20 mg/L tetracycline [48]	incl 0,0	4.32×10^{-4}	9.1	0.06	0.073	3.96×10^{-5}	833.9	9.8	1.10×10^{-2}	3.64×10^{-16}	19.9	0.017
	excl. 0,0	8.22×10^{-5}	9.6	0.55	0.070	3.96×10^{-5}	833.9	9.8	1.10×10^{-2}	3.64×10^{-16}	19.9	0.039
	incl 0 (eq 20)	4.74×10^{-4}	9.1	0.0	0.074							

[a] "Incl 0" means that the 0-absorption point at t = 0 is included as a 'measured' data point; "excl. 0" means that the 0-absorption point at t = 0 is not included as a 'measured' data point. The mono-exponential fits were performed with eq 21 except for the rows specified with eq 20; the involved integral fits were performed with eq 18. See main text for further details.
[b] pale green shading denotes the best fit options. More than one option is shaded in cases where similar goodness-of-fit were obtained and/or when data scattering precludes definitive discarding of the merits of a given fitting strategy as compared to another, *cf.* e.g. **Figure 5** in the main text.
[c] $c_{X,p}$(eq) corresponds to the equilibrium plateau value $c_{X,p}^{tot,\infty}$. See text for details.
[d]NRMSE = normalized root mean square error; the closer this value is to zero, the better the fit; R^2 = 1-NRMSE
[e] involved integral fit parameters are only entered for the cases for which a robust fitting of experimental data with eq 18 was obtained.

Table S3. Involved integral data fitting (eq 18): additional fitted and derived parameters

System	Fit[a,b]	τ (s)	γ	σ_w (m s^{-1})	σ_p (m s^{-1})	δ_p (m)	a (m)	$J_0^+ / c^*_{X,w}$ (m s^{-1})	NRMSE[c]
PS pristine, 0.5 ppm Cd [40]	incl 0	9576	0.41	2.75×10^{-3}	1.47×10^{-10}	5.31×10^{-8}	3×10^{-7}	2.06×10^{-10}	0.019
	excl. 0	9576	0.41	2.75×10^{-3}	1.47×10^{-10}	5.31×10^{-8}	3×10^{-7}	2.06×10^{-10}	0.023
PS aged 7 day H_2O_2, 0.5 ppm Cd [40]	incl 0	2182	10.41	2.75×10^{-3}	1.87×10^{-10}	6.05×10^{-9}	3×10^{-7}	2.14×10^{-9}	0.005
	excl. 0	2182	10.41	2.75×10^{-3}	1.87×10^{-10}	6.05×10^{-9}	3×10^{-7}	2.14×10^{-9}	0.008
PS aged 7 day Fenton, 0.5 ppm Cd [40]	incl 0	1898	9.73	2.75×10^{-3}	6.36×10^{-10}	7.08×10^{-9}	3×10^{-7}	6.83×10^{-9}	0.003
	excl. 0	1898	9.73	2.75×10^{-3}	6.36×10^{-10}	7.08×10^{-9}	3×10^{-7}	6.83×10^{-9}	0.005
PS pristine, 2 ppm Cd [40]	incl 0	5943	0.12	2.75×10^{-3}	7.44×10^{-11}	4.50×10^{-8}	3×10^{-7}	8.36×10^{-11}	0.029
	excl. 0	5943	0.12	2.75×10^{-3}	7.44×10^{-11}	4.50×10^{-8}	3×10^{-7}	8.36×10^{-11}	0.034
PS aged 7 day H_2O_2, 2 ppm Cd [40]	incl 0	1739	7.95	2.75×10^{-3}	1.49×10^{-10}	6.72×10^{-9}	3×10^{-7}	1.33×10^{-9}	0.014
	excl. 0	1739	7.95	2.75×10^{-3}	1.49×10^{-10}	6.72×10^{-9}	3×10^{-7}	1.33×10^{-10}	0.025
PS aged 7 day Fenton, 2 ppm Cd [40]	incl 0	1501	8.5	2.75×10^{-3}	5.63×10^{-10}	7.07×10^{-9}	3×10^{-7}	5.35×10^{-9}	0.014
	excl. 0	1501	8.5	2.75×10^{-3}	5.63×10^{-10}	7.07×10^{-9}	3×10^{-7}	5.35×10^{-9}	0.029
PP pristine, 10 mg/L tetracycline [41]	incl 0	3645	0.36	7.31×10^{-5}	5.30×10^{-8}	4.01×10^{-6}	3.83×10^{-5}	7.17×10^{-8}	0.009
	excl. 0	3645	0.36	7.31×10^{-5}	5.30×10^{-8}	4.01×10^{-6}	3.83×10^{-5}	7.17×10^{-8}	0.010
PP aged-UV, 10 mg/L tetracycline [41]	incl 0	1180	1.58	7.31×10^{-5}	5.83×10^{-8}	1.03×10^{-6}	3.83×10^{-5}	1.51×10^{-7}	0.013
	excl. 0	1180	1.58	7.31×10^{-5}	5.83×10^{-8}	1.03×10^{-6}	3.83×10^{-5}	1.51×10^{-7}	0.015
PP aged-Kps, 10 mg/L tetracycline [41]	incl 0	2993	1.28	7.31×10^{-5}	5.82×10^{-8}	2.05×10^{-6}	3.83×10^{-5}	1.33×10^{-7}	0.015
	excl. 0	2993	1.28	7.31×10^{-5}	5.82×10^{-8}	2.05×10^{-6}	3.83×10^{-5}	1.33×10^{-7}	0.016
PE aged-UV, 10 mg/L tetracycline [41]	incl 0	1212	1.19	7.14×10^{-5}	5.95×10^{-8}	1.43×10^{-6}	4.34×10^{-5}	1.30×10^{-7}	0.011
	excl. 0	1212	1.19	7.14×10^{-5}	5.95×10^{-8}	1.43×10^{-6}	4.34×10^{-5}	1.30×10^{-8}	0.012
PE aged-Kps, 10 mg/L tetracycline [41]	incl 0	4361	0.33	7.14×10^{-5}	7.15×10^{-8}	4.47×10^{-6}	4.34×10^{-5}	9.53×10^{-8}	0.018
	excl. 0	4361	0.33	7.14×10^{-5}	7.15×10^{-8}	4.47×10^{-6}	4.34×10^{-5}	9.53×10^{-8}	0.020
PBAT pristine, 10 mg/L tetracycline [41]	incl 0	1343	2.48	7.32×10^{-5}	9.44×10^{-8}	1.58×10^{-6}	3.82×10^{-5}	3.28×10^{-7}	0.022
	excl. 0	1343	2.48	7.32×10^{-5}	9.44×10^{-8}	1.58×10^{-6}	3.82×10^{-5}	3.28×10^{-7}	0.027
PBAT aged-UV, 10 mg/L tetracycline [41]	incl 0	333	3.39	7.32×10^{-5}	2.20×10^{-7}	7.90×10^{-7}	3.82×10^{-5}	9.61×10^{-7}	0.021
	excl. 0	333	3.39	7.32×10^{-5}	2.20×10^{-7}	7.90×10^{-7}	3.82×10^{-5}	9.61×10^{-7}	0.027
PBAT aged-Kps, 10 mg/L tetracycline [41]	incl 0	22	74.66	7.32×10^{-5}	2.19×10^{-7}	3.80×10^{-8}	3.82×10^{-5}	1.65×10^{-5}	0.022
	excl. 0	22	73.98	7.32×10^{-5}	2.19×10^{-7}	3.83×10^{-8}	3.82×10^{-5}	1.65×10^{-5}	0.031

System	Fit[a,b]	τ (s)	γ	σ_w (m s^{-1})	σ_p (m s^{-1})	δ_p (m)	a (m)	$J_0^+ / c_{X,w}^*$ (m s^{-1})	NRMSE[c]
PS pristine, 10 mg/L tetracycline [41]	incl 0	3997	1.98	6.81×10^{-5}	6.51×10^{-8}	3.26×10^{-6}	5.73×10^{-5}	1.94×10^{-7}	0.018
	excl. 0	3997	1.98	6.81×10^{-5}	6.51×10^{-8}	3.26×10^{-6}	5.73×10^{-5}	1.94×10^{-7}	0.021
PS aged-UV, 10 mg/L tetracycline [41]	incl 0	4517	0.05	6.81×10^{-5}	9.54×10^{-8}	7.25×10^{-6}	5.73×10^{-5}	1.00×10^{-7}	0.012
	excl. 0	4517	0.05	6.81×10^{-5}	9.54×10^{-8}	7.25×10^{-6}	5.73×10^{-5}	1.00×10^{-7}	0.013
PS aged-Kps, 10 mg/L tetracycline [41]	incl 0	443	2.86	6.81×10^{-5}	1.51×10^{-7}	7.69×10^{-7}	5.73×10^{-5}	5.80×10^{-7}	0.008
	excl. 0	443	2.86	6.81×10^{-5}	1.51×10^{-7}	7.69×10^{-7}	5.73×10^{-5}	5.80×10^{-7}	0.010
PE pristine, 5 mg/L atrazine [42]	incl 0	192.9	18.2	5.67×10^{-5}	1.75×10^{-7}	3.10×10^{-7}	8.5×10^{-5}	3.36×10^{-6}	0.015
	excl. 0	195.0	18.0	5.67×10^{-5}	1.75×10^{-7}	3.13×10^{-7}	8.5×10^{-5}	3.32×10^{-6}	0.026
PE aged, 5 mg/L atrazine [42]	incl 0	1374.4	1.04	5.67×10^{-5}	3.77×10^{-7}	2.70×10^{-6}	9.0×10^{-5}	7.63×10^{-7}	0.031
	excl. 0	1374.4	1.04	5.67×10^{-5}	3.77×10^{-7}	2.70×10^{-6}	9.0×10^{-5}	7.63×10^{-7}	0.046
PP pristine, 5 mg/L atrazine [42]	incl 0	194.9	23.72	5.67×10^{-5}	2.30×10^{-7}	5.34×10^{-7}	1.5×10^{-4}	5.67×10^{-6}	0.014
	excl. 0	195.8	23.60	5.67×10^{-5}	2.30×10^{-7}	5.37×10^{-7}	1.5×10^{-4}	5.65×10^{-6}	0.026
PP aged, 5 mg/L atrazine [42]	incl 0	192.9	9.00	5.67×10^{-5}	5.10×10^{-7}	7.08×10^{-7}	1.8×10^{-4}	5.10×10^{-6}	0.024
	excl. 0	191.2	9.08	5.67×10^{-5}	5.10×10^{-7}	7.02×10^{-7}	1.8×10^{-4}	5.14×10^{-6}	0.037
PS pristine, 5 mg/L atrazine [42]	incl 0	193.3	19.2	5.67×10^{-5}	1.03×10^{-7}	1.69×10^{-7}	4.8×10^{-5}	2.07×10^{-6}	0.036
	excl. 0	193.7	19.1	5.67×10^{-5}	1.03×10^{-7}	1.69×10^{-7}	4.8×10^{-5}	2.07×10^{-6}	0.063
PS aged, 5 mg/L atrazine [42]	incl 0	190.8	14.9	5.67×10^{-5}	1.59×10^{-7}	2.23×10^{-7}	5.2×10^{-5}	2.52×10^{-6}	0.016
	excl. 0	188.2	15.1	5.67×10^{-5}	1.59×10^{-7}	2.20×10^{-7}	5.2×10^{-5}	2.56×10^{-6}	0.028
PS aged, 10 mg/L ciprofloxacin [43]	incl 0	1824.3	15.9	7.85×10^{-6}	9.37×10^{-8}	2.73×10^{-7}	3.75×10^{-5}	1.57×10^{-6}	0.022
	excl. 0	1824.3	15.9	7.85×10^{-6}	9.37×10^{-8}	2.73×10^{-7}	3.75×10^{-5}	1.57×10^{-6}	0.036
PVC pristine, 10 mg/L ciprofloxacin [43]	incl 0	18641	7.94	7.85×10^{-6}	1.54×10^{-8}	5.14×10^{-7}	3.75×10^{-5}	1.37×10^{-7}	0.043
	excl. 0	18641	7.94	7.85×10^{-6}	1.54×10^{-8}	5.14×10^{-7}	3.75×10^{-5}	1.37×10^{-7}	0.049
PVC aged, 10 mg/L ciprofloxacin [43]	incl 0	2123.4	17.7	7.85×10^{-6}	6.82×10^{-8}	4.06×10^{-7}	3.75×10^{-5}	1.27×10^{-6}	0.018
	excl. 0	2123.4	17.7	7.85×10^{-6}	6.82×10^{-8}	4.06×10^{-7}	3.75×10^{-5}	1.27×10^{-6}	0.040
PS pristine, 1 mg/L Cd [44]	incl 0	7798.5	2.1	9.07×10^{-5}	7.18×10^{-8}	2.79×10^{-6}	7.5×10^{-5}	2.25×10^{-7}	0.026
	excl. 0	7798.5	2.1	9.07×10^{-5}	7.18×10^{-8}	2.79×10^{-8}	7.5×10^{-5}	2.25×10^{-7}	0.029
PS aged, 1 mg/L Cd [44]	incl 0	4787.8	1.8	9.07×10^{-5}	1.72×10^{-7}	3.79×10^{-6}	7.5×10^{-5}	4.78×10^{-7}	0.010
	excl. 0	4787.8	1.8	9.07×10^{-5}	1.72×10^{-7}	3.79×10^{-6}	7.5×10^{-5}	4.78×10^{-7}	0.012
PVC pristine, 1 mg/L Cd [44]	incl 0	2292.2	0.5	9.07×10^{-5}	2.22×10^{-7}	3.20×10^{-6}	7.5×10^{-5}	3.38×10^{-7}	0.026
	excl. 0	2292.2	0.5	9.07×10^{-5}	2.22×10^{-7}	3.20×10^{-6}	7.5×10^{-5}	3.38×10^{-7}	0.031

System	Fit[a,b]	τ (s)	γ	σ_w (m s^{-1})	σ_p (m s^{-1})	δ_p (m)	a (m)	$J_0^+ / c^*_{X,w}$ (m s^{-1})	NRMSE[c]
PVC aged, 1 mg/L Cd [44]	incl 0	3331.0	3.0	9.07×10^{-5}	5.00×10^{-7}	3.26×10^{-6}	7.5×10^{-5}	2.01×10^{-6}	0.011
	excl. 0	3331.0	3.0	9.07×10^{-5}	5.00×10^{-7}	3.26×10^{-6}	7.5×10^{-5}	2.01×10^{-6}	0.015
PVC pristine, 10 mg/L carbamazepine [45]	incl 0	8018.1	1.9	5.62×10^{-5}	4.35×10^{-8}	2.62×10^{-6}	8.05×10^{-5}	1.25×10^{-7}	0.017
	excl. 0	8018.1	1.9	5.62×10^{-5}	4.35×10^{-8}	2.62×10^{-6}	8.05×10^{-5}	1.25×10^{-7}	0.019
PVC aged, 10 mg/L carbamazepine [45]	incl 0	6689.4	3.4	1.4×10^{-4}	8.25×10^{-9}	2.64×10^{-7}	5.55×10^{-6}	3.66×10^{-8}	0.006
	excl. 0	6689.4	3.4	1.4×10^{-4}	8.25×10^{-9}	2.64×10^{-7}	5.55×10^{-6}	3.66×10^{-8}	0.008
PE pristine, 10 mg/L carbamazepine [45]	incl 0	17264	5.7	5.44×10^{-5}	3.97×10^{-8}	3.20×10^{-6}	1.13×10^{-4}	2.66×10^{-7}	0.013
	excl. 0	17264	5.7	5.44×10^{-5}	3.97×10^{-8}	3.20×10^{-6}	1.13×10^{-4}	2.66×10^{-7}	0.015
PET pristine, 10 mg/L carbamazepine [45]	incl 0	6385.8	1.6	5.47×10^{-5}	1.03×10^{-7}	4.47×10^{-6}	1.06×10^{-4}	2.67×10^{-7}	0.017
	excl. 0	6385.8	1.6	5.47×10^{-5}	1.03×10^{-7}	4.47×10^{-6}	1.06×10^{-4}	2.67×10^{-7}	0.019
PU pristine, 10 mg/L Cu [46]	incl 0	407.8	9.0	8.40×10^{-5}	7.22×10^{-9}	1.03×10^{-7}	5×10^{-5}	7.22×10^{-8}	0.033
	excl. 0	418.4	8.8	8.40×10^{-5}	7.22×10^{-9}	1.05×10^{-7}	5×10^{-5}	7.06×10^{-8}	0.042
PU aged, 10 mg/L Cu [46]	incl 0	3978.2	3.9	8.40×10^{-5}	1.01×10^{-8}	1.15×10^{-6}	5×10^{-5}	4.96×10^{-8}	0.037
	excl. 0	3978.2	3.9	8.40×10^{-5}	1.01×10^{-8}	1.15×10^{-6}	5×10^{-5}	4.96×10^{-8}	0.057
PU pristine, 10 mg/L oxytetracycline [46]	incl 0	389.8	41.4	6.96×10^{-5}	6.31×10^{-9}	8.27×10^{-8}	5×10^{-5}	2.67×10^{-7}	0.027
	excl. 0	379.6	42.5	6.96×10^{-5}	6.31×10^{-9}	8.05×10^{-8}	5×10^{-5}	2.74×10^{-7}	0.040
PU aged, 10 mg/L oxytetracycline [46]	incl 0	7704.1	74.3	6.96×10^{-5}	9.69×10^{-10}	1.00×10^{-7}	5×10^{-5}	7.30×10^{-8}	0.007
	excl. 0	7704.1	74.3	6.96×10^{-5}	9.69×10^{-10}	1.00×10^{-7}	5×10^{-5}	7.30×10^{-8}	0.015
PP aged, 10 mg/L oxytetracycline [47]	incl 0	4192.2	160.3	6.13×10^{-5}	1.57×10^{-8}	1.66×10^{-12}	1.775×10^{-4}	2.53×10^{-6}	0.028
	excl. 0	4192.2	160.3	6.13×10^{-5}	1.57×10^{-8}	1.72×10^{-12}	1.775×10^{-4}	2.53×10^{-6}	0.042
PP pristine, 10 mg/L chloramphenicol [47]	incl 0	2232.8	6.3	6.97×10^{-5}	1.08×10^{-7}	3.27×10^{-6}	1.775×10^{-4}	7.82×10^{-7}	0.033
	excl. 0	2232.8	6.3	6.97×10^{-5}	1.08×10^{-7}	3.27×10^{-6}	1.775×10^{-4}	7.82×10^{-7}	0.039
PP aged, 10 mg/L chloramphenicol [47]	incl 0	7885.2	14.4	6.97×10^{-5}	6.31×10^{-8}	1.29×10^{-6}	1.775×10^{-4}	9.72×10^{-7}	0.023
	excl. 0	7885.2	14.4	6.97×10^{-5}	6.31×10^{-8}	1.29×10^{-6}	1.775×10^{-4}	9.72×10^{-7}	0.025
PP pristine, 10 mg/L sulfamethoxazole [47]	incl 0	9413.7	57.1	5.28×10^{-5}	2.02×10^{-9}	2.42×10^{-11}	1.775×10^{-4}	1.17×10^{-7}	0.033
	excl. 0	9413.7	57.1	5.28×10^{-5}	2.02×10^{-9}	2.42×10^{-11}	1.775×10^{-4}	1.17×10^{-7}	0.038
PP aged, 10 mg/L sulfamethoxazole [47]	incl 0	982.3	12.0	5.28×10^{-5}	5.36×10^{-8}	9.01×10^{-7}	1.775×10^{-4}	6.95×10^{-7}	0.016
	excl. 0	982.3	12.0	5.28×10^{-5}	5.36×10^{-8}	9.01×10^{-7}	1.775×10^{-4}	6.95×10^{-7}	0.019
PP pristine, 10 mg/L sulfamethazine [47]	incl 0	1897.5	20.8	5.28×10^{-5}	1.34×10^{-8}	1.18×10^{-6}	1.775×10^{-4}	2.91×10^{-7}	0.028
	excl. 0	1897.5	20.8	5.28×10^{-5}	1.34×10^{-8}	1.18×10^{-6}	1.775×10^{-4}	2.91×10^{-7}	0.033

System	Fit[a,b]	τ (s)	γ	σ_w (m s^{-1})	σ_p (m s^{-1})	δ_p (m)	a (m)	$J_0^+ / c^*_{X,w}$ (m s^{-1})	NRMSE[c]
PP aged, 10 mg/L sulfamethazine [47]	incl 0	1203.8	3.1	5.28×10^{-5}	2.09×10^{-7}	7.67×10^{-6}	1.775×10^{-4}	8.58×10^{-7}	0.007
	excl. 0	1203.8	3.1	5.28×10^{-5}	2.09×10^{-7}	7.67×10^{-6}	1.775×10^{-4}	8.58×10^{-7}	0.009
PP pristine, 10 mg/L enrofloxacin [47]	incl 0	4599.7	36.3	8.45×10^{-6}	1.28×10^{-8}	7.60×10^{-7}	1.775×10^{-4}	4.75×10^{-7}	0.033
	excl. 0	4599.7	36.3	8.45×10^{-6}	1.28×10^{-8}	7.60×10^{-7}	1.775×10^{-4}	4.75×10^{-7}	0.036
PP aged, 10 mg/L enrofloxacin [47]	incl 0	1366.3	30.7	8.45×10^{-6}	4.70×10^{-8}	3.98×10^{-7}	1.775×10^{-4}	1.48×10^{-6}	0.053
	excl. 0	1366.3	30.7	8.45×10^{-6}	4.70×10^{-8}	3.98×10^{-7}	1.775×10^{-4}	1.48×10^{-6}	0.063
PP aged, 10 mg/L ofloxacin [47]	incl 0	10675	6.7	3.59×10^{-5}	3.22×10^{-8}	4.81×10^{-6}	1.775×10^{-4}	2.49×10^{-7}	0.036
	excl. 0	10675	6.7	3.59×10^{-5}	3.22×10^{-8}	4.81×10^{-6}	1.775×10^{-4}	2.49×10^{-7}	0.042
PP pristine, 10 mg/L norfloxacin [47]	incl 0	699.8	1.84	5.28×10^{-5}	1.14×10^{-7}	6.41×10^{-6}	1.775×10^{-4}	3.24×10^{-7}	0.022
	excl. 0	699.8	1.84	5.28×10^{-5}	1.14×10^{-7}	6.41×10^{-6}	1.775×10^{-4}	3.24×10^{-7}	0.029
PP aged, 10 mg/L norfloxacin [47]	incl 0	4594	10.0	5.28×10^{-5}	3.25×10^{-8}	2.41×10^{-6}	1.775×10^{-4}	3.57×10^{-7}	0.039
	excl. 0	4594	10.0	5.28×10^{-5}	3.25×10^{-8}	2.41×10^{-6}	1.775×10^{-4}	3.57×10^{-7}	0.046
PP pristine, 10 mg/L sulfamerazine [47]	incl 0	1693.1	7.7	5.28×10^{-5}	5.55×10^{-8}	2.91×10^{-6}	1.775×10^{-4}	4.81×10^{-7}	0.020
	excl. 0	1693.1	7.7	5.28×10^{-5}	5.55×10^{-8}	2.91×10^{-6}	1.775×10^{-4}	4.81×10^{-7}	0.023
PP aged, 10 mg/L sulfamerazine [47]	incl 0	1886.7	10.0	5.28×10^{-5}	6.37×10^{-8}	1.04×10^{-6}	1.775×10^{-4}	6.97×10^{-7}	0.029
	excl. 0	1886.7	10.0	5.28×10^{-5}	6.37×10^{-8}	1.04×10^{-6}	1.775×10^{-4}	6.97×10^{-7}	0.033
PP pristine, 10 mg/L sulfathiazole [47]	incl 0	420.9	13.0	5.28×10^{-5}	1.97×10^{-7}	1.38×10^{-6}	1.775×10^{-4}	2.75×10^{-6}	0.027
	excl. 0	420.9	13.0	5.28×10^{-5}	1.97×10^{-7}	1.38×10^{-6}	1.775×10^{-4}	2.75×10^{-6}	0.038
PP aged, 10 mg/L sulfathiazole [47]	incl 0	26.2	63.2	5.28×10^{-5}	9.37×10^{-7}	2.15×10^{-7}	1.775×10^{-4}	5.92×10^{-5}	0.030
	excl. 0	25.2	65.8	5.28×10^{-5}	9.37×10^{-7}	2.06×10^{-7}	1.775×10^{-4}	6.15×10^{-5}	0.047
PP pristine, 10 mg/L tetracycline [47]	incl 0	1100.7	0.9	6.13×10^{-5}	5.11×10^{-8}	4.49×10^{-6}	1.775×10^{-4}	9.92×10^{-8}	0.014
	excl. 0	1100.7	0.9	6.13×10^{-5}	5.11×10^{-8}	4.49×10^{-6}	1.775×10^{-4}	9.92×10^{-8}	0.016
PP aged, 10 mg/L tetracycline [47]	incl 0	33.4	71.1	6.13×10^{-5}	1.63×10^{-7}	1.40×10^{-7}	1.775×10^{-4}	1.17×10^{-5}	0.048
	excl. 0	34.1	69.7	6.13×10^{-5}	1.63×10^{-7}	1.43×10^{-7}	1.775×10^{-4}	1.15×10^{-5}	0.065
PS pristine, 20 mg/L tetracycline [48]	incl 0	422.7	30.0	6.96×10^{-5}	3.88×10^{-9}	2.60×10^{-7}	5×10^{-5}	1.20×10^{-7}	0.009
	excl. 0	422.7	30.0	6.96×10^{-5}	3.88×10^{-9}	2.60×10^{-7}	5×10^{-5}	1.20×10^{-7}	0.018
PS aged, 20 mg/L tetracycline [48]	incl 0	833.9	18.9	6.96×10^{-5}	6.46×10^{-9}	5.51×10^{-7}	5×10^{-5}	1.28×10^{-7}	0.017
	excl. 0	833.9	18.9	6.96×10^{-5}	6.46×10^{-9}	5.51×10^{-7}	5×10^{-5}	1.28×10^{-7}	0.039

[a] "Incl 0" means that the 0-absorption point at t = 0 is included as a 'measured' data point; "excl. 0" means that the 0-absorption point at t = 0 is not included as a 'measured' data point.
[b] pale green shading denotes the best fit options. [c]NRMSE = normalized root mean square error; the closer this value is to zero, the better the fit; R^2 = 1-NRMSE.

Table S4. Summary of equilibrium water/polymer partition coefficient values, $K_{w,p}$, derived from data fitting using (i) mono-exponential expression (eq 20 or 21) and (ii) the involved integral expression (eq 18), and from evaluation (iii) of the slope of the experimental Henry 'equilibrium' isotherms. Values are entered for both fits to mono-exponential and integral expressions if both provide a comparable and adequate description of the data, otherwise only the value for the superior fit (lowest NRMSE) is given. $K_{w,p}$ values for all fitting modes are collected in **Table S2**.

System	mono-exp eq 20 or 21	Integral eq 18	Henry	Comments
PS pristine, 0.5 ppm Cd [40]	26.64		5.2	24 hr equilibration time used for isotherm measurements; from the absorption kinetic plots this time is indeed sufficient to warrant equilibration of the contaminant absorption process (**Fig. 2** in main text, **Figs. S4** and **S5** in SI).
PS aged 7 day H_2O_2, 0.5 ppm Cd [40]		67.6	29.6	
PS aged 7 day Fenton, 0.5 ppm Cd [40]		170.5	106.2	
PS pristine, 2 ppm Cd [40]	9.8		5.2	
PS aged 7 day H_2O_2, 2 ppm Cd [40]		38.6	29.6	
PS aged 7 day Fenton, 2 ppm Cd [40]		119.6	106.2	
PP pristine, 10 mg/L tetracycline [41]	47.4		34.3	10 hr equilibration time used for isotherm measurements; from the absorption kinetic plots, this time is not really long enough: there is still some ongoing increase of the amount of absorbed contaminant at long times in some cases (**Figs. S6-S9** in SI).
PP aged-UV, 10 mg/L tetracycline [41]	64.7	66.7	25.0	
PP aged-Kps, 10 mg/L tetracycline [41]	79.6	85.1	36.1	
PE pristine, 10 mg/L tetracycline [41]	38.4		24.5	
PE aged-UV, 10 mg/L tetracycline [41]	49.4	50.5	24.8	
PE aged-Kps, 10 mg/L tetracycline [41]	68.4	69.8	32.5	
PBAT pristine, 10 mg/L tetracycline [41]		80.4	30.9	
PBAT aged-UV, 10 mg/L tetracycline [41]		92.7	36.3	
PBAT aged-Kps, 10 mg/L tetracycline [41]		127.6	41.5	
PS pristine, 10 mg/L tetracycline [41]	72.5	79.8	29.3	
PS aged-UV, 10 mg/L tetracycline [41]	59.0	59.5	36.3	
PS aged-Kps, 10 mg/L tetracycline [41]	85.4	86.8	40.5	
PE pristine, 5 mg/L atrazine [42]		109.3	80.3	96 hr equilibration time used for isotherm measurements; from the kinetic plots, this time is indeed sufficient to warrant equilibration of the contaminant absorption process (**Fig. 5** in main text, **Figs. S10** and **S11** in SI).
PE aged, 5 mg/L atrazine [42]	189.0	192.1	189.1	
PP pristine, 5 mg/L atrazine [42]		84.1	72.7	
PP aged, 5 mg/L atrazine [42]	136.9	139.1	146.4	
PS pristine, 5 mg/L atrazine [42]	114.2	118.0	80.3	
PS aged, 5 mg/L atrazine [42]	132.5	136.6	130.5	
PS pristine, 10 mg/L ciprofloxacin [43]	255.3		258.2	Equilibration time for isotherm measurements is not stated.
PS aged, 10 mg/L ciprofloxacin [43]	539.9	626.1	317.8	
PVC pristine, 10 mg/L ciprofloxacin [43]	271	558.6	255.2	
PVC aged, 10 mg/L ciprofloxacin [43]	306.9	356.5	250	
PS pristine, 1 mg/L Cd [44]		200.6	205.1	Equilibration time for isotherm measurements is not explicitly stated, but is probably 48 hr, which is sufficient to warrant equilibration of the contaminant absorption process (**Fig. 4** in main text, **Fig. S14** in SI).
PS aged, 1 mg/L Cd [44]		216.8	458.6	
PVC pristine, 1 mg/L Cd [44]	157.6	158.7	245.8	
PVC aged, 1 mg/L Cd [44]		511.5	594.2	
PVC pristine, 10 mg/L carbamazepine [45]		133.3	60.1	48 hr equilibration time used for isotherm measurements; from the kinetic plots, this time is not really long enough: there is still some ongoing increase of the amount of absorbed contaminant at long times (**Figs. S15** and **S16** in SI).
PVC aged, 10 mg/L carbamazepine [45]		208.8	75.1	
PE pristine, 10 mg/L carbamazepine [45]		214.0	43.7	
PET pristine, 10 mg/L carbamazepine [45]		147.3	51.6	
PU pristine, 10 mg/L Cu [46]	28.4		23.7	48 hr equilibration time used for isotherm measurements; from the kinetic plots this time is not really long enough: there is still some ongoing increase of the amount of absorbed contaminant at long times (**Figs. S17** and **S18** in SI.
PU aged, 10 mg/L Cu [46]		34.9	19.8	
PU pristine, 10 mg/L oxytetracycline [46]		29.7	27.3	
PU aged, 10 mg/L oxytetracycline [46]		74.3	40.3	
PP pristine, 10 mg/L oxytetracycline [47]	126.6			No Henry isotherm data for pristine PP.
PP aged, 10 mg/L oxytetracycline [47]	219.5	3.96×10^7	133	Equilibration time for isotherm measurements is not stated.
PP pristine, 10 mg/L chloramphenicol [47]		73.6		No Henry isotherm data for pristine PP.

System	mono-exp eq 20 or 21	Integral eq 18	Henry	Comments
PP aged, 10 mg/L chloramphenicol [47]		384.9	55	Equilibration time for isotherm measurements is not stated.
PP pristine, 10 mg/L sulfamethoxazole [47]	24.2	7.8×10^{5}		No Henry isotherm data for pristine PP.
PP aged, 10 mg/L sulfamethoxazole [47]		58.5	12.9	Equilibration time for isotherm measurements is not stated.
PP pristine, 10 mg/L sulfamethazine [47]		21.5		No Henry isotherm data for pristine PP.
PP aged, 10 mg/L sulfamethazine [47]		32.7	15.1	Equilibration time for isotherm measurements is not stated.
PP pristine, 10 mg/L enrofloxacin [47]		77.3		No Henry isotherm data for pristine
PP aged, 10 mg/L enrofloxacin [47]		161.6	58.0	Equilibration time for isotherm measurements not stated.
PP pristine, 10 mg/L ciprofloxacin [47]	76.0			No Henry isotherm data for pristine.
PP aged, 10 mg/L ciprofloxacin [47]	152.9		19.6	Equilibration time for isotherm measurements not stated.
PP pristine, 10 mg/L ofloxacin [47]	19.8			No Henry isotherm data for pristine.
PP aged, 10 mg/L ofloxacin [47]		71.5	19.6	Equilibration time for isotherm measurements not stated.
PP pristine, 10 mg/L norfloxacin [47]	12.3	12.5		No Henry isotherm data for pristine PP.
PP aged, 10 mg/L norfloxacin [47]		61.8	31.2	Equilibration time for isotherm measurements is not stated.
PP pristine, 10 mg/L sulfamerazine [47]		32.3		No Henry isotherm data for pristine PP.
PP aged, 10 mg/L sulfamerazine [47]		115.7	18.3	Equilibration time for isotherm measurements is not stated.
PP pristine, 10 mg/L sulfathiazole [47]		60.2		No Henry isotherm data for pristine PP.
PP aged, 10 mg/L sulfathiazole [47]	113.4	114.5	28.2	Equilibration time for isotherm measurements is not stated.
PP pristine, 10 mg/L tetracycline [47]	12.5	12.5		No Henry isotherm data for pristine PP.
PP aged, 10 mg/L tetracycline [47]	38.8	38.9	51.1	Equilibration time for isotherm measurements is not stated.
PS pristine, 20 mg/L tetracycline [48]		6.3	6.2	36 hr equilibration time used for isotherm measurements; from the kinetic plots, this time is indeed sufficient to warrant equilibration of the contaminant absorption process (**Fig. 3** in main text).
PS aged, 20 mg/L tetracycline [48]		9.8	10.1	

Table S5. Summary of values adopted for $D_{X,w}$ and particle radius, *a*, and sensitivity of $D_{X,p}$ to *a*

"na" in the $D_{X,p}$ column means "not applicable" because the data fitting according to eq 18 was not robust (in such cases the $D_{X,w}$ and *a* are also immaterial). If no literature value was available for $D_{X,w}$, a value of 5×10^{-10} m^2 s^{-1} was considered (the results are anyway insensitive to this value; see explanation in main text).

System	$D_{X,w}$ (m^2 s^{-1})	*a* (m) used in calculations	$D_{X,p}$ computed (m^2 s^{-1})	Reported range of *a* (m)	Computed range of $D_{X,p}$ (m^2 s^{-1}) for range of *a*
PS pristine, 0.5 ppm Cd [40]	8×10^{-10} [S1]	3×10^{-7}	2.94×10^{-19}	No range reported. SEM images show reasonably homogenously sized particles	-
PS aged 7 day H_2O_2, 0.5 ppm Cd [40]	8×10^{-10}	3×10^{-7}	1.67×10^{-20}		-
PS aged 7 day Fenton, 0.5 ppm Cd [40]	8×10^{-10}	3×10^{-7}	2.64×10^{-20}		-
PS pristine, 2 ppm Cd [40]	8×10^{-10}	3×10^{-7}	3.41×10^{-19}		-
PS aged 7 day H_2O_2, 2 ppm Cd [40]	8×10^{-10}	3×10^{-7}	2.60×10^{-20}		-
PS aged 7 day Fenton, 2 ppm Cd [40]	8×10^{-10}	3×10^{-7}	3.33×10^{-20}		-
PP pristine, 10 mg/L tetracycline [41]	5.8×10^{-10} [S2]	3.83×10^{-5} (50th percentile)	4.42×10^{-15}	25th percentile: 3.425×10^{-5} m<br>95th percentile: 6.345×10^{-5} m	3.53×10^{-15} – 1.21×10^{-14}
PP aged-UV, 10 mg/L tetracycline [41]	5.8×10^{-10} [S2]	3.83×10^{-5}	9.03×10^{-16}	Assuming same as pristine:<br>25th percentile: 3.425×10^{-5} m<br>95th percentile: 6.345×10^{-5} m	7.22×10^{-16} – 2.48×10^{-15}
PP aged-Kps, 10 mg/L tetracycline [41]	5.8×10^{-10} [S2]	3.83×10^{-5}	1.40×10^{-15}	Assuming same as pristine:<br>25th percentile: 3.425×10^{-5} m<br>95th percentile: 6.345×10^{-5} m	1.12×10^{-15} – 3.85×10^{-15}
PE pristine, 10 mg/L tetracycline [41]	5.8×10^{-10} [S2]	4.34×10^{-5} (50th percentile; values only given for pristine, assume aged is the same)	na	25th percentile: 3.58×10^{-5} m<br>95th percentile: 6.24×10^{-5} m	-
PE aged-UV, 10 mg/L tetracycline [41]	5.8×10^{-10} [S2]	4.34×10^{-5}	1.68×10^{-15}	Assuming same as pristine:<br>25th percentile: 3.58×10^{-5} m<br>95th percentile: 6.24×10^{-5} m	1.14×10^{-15} – 3.48×10^{-15}
PE aged-Kps, 10 mg/L tetracycline [41]	5.8×10^{-10} [S2]	4.34×10^{-5}	4.58×10^{-15}	Assuming same as pristine:<br>25th percentile: 3.58×10^{-5} m<br>95th percentile: 6.24×10^{-5} m	3.12×10^{-15} – 9.49×10^{-15}
PBAT pristine, 10 mg/L tetracycline [41]	5.8×10^{-10} [S2]	3.82×10^{-5} (50th percentile; values only given for pristine, assume aged is the same)	1.85×10^{-15}	25th percentile: 3.46×10^{-5} m<br>95th percentile: 6.09×10^{-5} m	1.52×10^{-15} – 4.72×10^{-15}

System	$D_{X,w}$ (m^2 s^{-1})	*a* (m) used in calculations	$D_{X,p}$ computed (m^2 s^{-1})	Reported range of *a* (m)	Computed range of $D_{X,p}$ (m^2 s^{-1}) for range of *a*
PBAT aged-UV, 10 mg/L tetracycline [41]	5.8×10^{-10} (S2)	3.82×10^{-5}	1.87×10^{-15}	Assuming same as pristine: 25th percentile: 3.46×10^{-5} m 95th percentile: 6.09×10^{-5} m	1.53×10^{-15} – 4.78×10^{-15}
PBAT aged-Kps, 10 mg/L tetracycline [41]	5.8×10^{-10} (S2)	3.82×10^{-5}	6.51×10^{-17}	Assuming same as pristine: 25th percentile: 3.46×10^{-5} m 95th percentile: 6.09×10^{-5} m	5.34×10^{-17} – 1.66×10^{-16}
PS pristine, 10 mg/L tetracycline [41]	5.8×10^{-10} (S2)	5.73×10^{-5} (50th percentile; values only given for pristine, assume aged is the same)	2.66×10^{-15}	25th percentile: 5.085×10^{-5} m 95th percentile: 1.008×10^{-4} m	2.10×10^{-15} – 8.26×10^{-15}
PS aged-UV, 10 mg/L tetracycline [41]	5.8×10^{-10} (S2)	5.73×10^{-5}	1.16×10^{-14}	Assuming same as pristine: 25th percentile: 5.085×10^{-5} m 95th percentile: 1.008×10^{-4} m	9.15×10^{-15} – 3.61×10^{-14}
PS aged-Kps, 10 mg/L tetracycline [41]	5.8×10^{-10} (S2)	5.73×10^{-5}	1.34×10^{-15}	Assuming same as pristine: 25th percentile: 5.085×10^{-5} m 95th percentile: 1.008×10^{-4} m	1.05×10^{-15} – 4.15×10^{-15}
PE pristine, 5 mg/L atrazine [42]	5×10^{-10} (S3)	8.5×10^{-5}	4.97×10^{-16}	7.05×10^{-5} – 9.85×10^{-5} m, estimated from SEM images.	3.41×10^{-16} – 6.68×10^{-16}
PE aged, 5 mg/L atrazine [42]	5×10^{-10} (S3)	9.0×10^{-5}	5.30×10^{-15}	Estimated from single SEM image.	-
PP pristine, 5 mg/L atrazine [42]	5×10^{-10} (S3)	1.5×10^{-4}	1.46×10^{-15}	Estimated from single SEM image.	-
PP aged, 5 mg/L atrazine [42]	5×10^{-10} (S3)	1.8×10^{-4}	2.60×10^{-15}	Estimated from single SEM image.	-
PS pristine, 5 mg/L atrazine [42]	5×10^{-10} (S3)	4.8×10^{-5}	1.48×10^{-16}	3.30×10^{-5} – 6.25×10^{-5} m, estimated from SEM images.	6.97×10^{-17} – 2.51×10^{-16}
PS aged, 5 mg/L atrazine [42]	5×10^{-10} (S3)	5.2×10^{-5}	2.60×10^{-16}	2.95×10^{-5} – 7.40×10^{-5} m, estimated from SEM images.	1.48×10^{-16} – 5.28×10^{-16}
PS pristine, 10 mg/L ciprofloxacin [43]	6.2×10^{-11} (S4)	3.75×10^{-5}	na	Only the average radius was reported.	-
PS aged, 10 mg/L ciprofloxacin [43]	6.2×10^{-11} (S4)	3.75×10^{-5}	4.09×10^{-17}		-
PVC pristine, 10 mg/L ciprofloxacin [43]	6.2×10^{-11} (S4)	3.75×10^{-5}	1.42×10^{-17}		-
PVC aged, 10 mg/L ciprofloxacin [43]	6.2×10^{-11} (S4)	3.75×10^{-5}	7.77×10^{-17}		-
PS pristine, 1 mg/L Cd [44]	8×10^{-10} (S1)	7.5×10^{-5}	1.00×10^{-15}	Range of radii for pristine reported as 7.5×10^{-5} to	1.00×10^{-15} – 1.35×10^{-14}
PS aged, 1 mg/L Cd [44]	8×10^{-10} (S1)	7.5×10^{-5}	3.01×10^{-15}		3.01×10^{-15} – 4.09×10^{-14}

System	$D_{X,w}$ (m^2 s^{-1})	*a* (m) used in calculations	$D_{X,p}$ computed (m^2 s^{-1})	Reported range of *a* (m)	Computed range of $D_{X,p}$ (m^2 s^{-1}) for range of *a*
PVC pristine, 1 mg/L Cd [44]	8×10^{-10} [S1]	7.5×10^{-5}	4.47×10^{-15}	2.75×10^{-4} m; assume aged is the same	$4.47\times10^{-15} – 6.09\times10^{-14}$
PVC aged, 1 mg/L Cd [44]	8×10^{-10} [S1]	7.5×10^{-5}	3.19×10^{-15}		$3.19\times10^{-15} – 4.43\times10^{-14}$
PVC pristine, 10 mg/L carbamazepine [45]	5×10^{-10}	8.05×10^{-5}	8.56×10^{-16}	Average size taken from Fig 2; range of radii taken from Fig S1: 25 to 150 μm	$8.25\times10^{-17} – 2.98\times10^{-15}$
PVC aged, 10 mg/L carbamazepine [45]	5×10^{-10}	5.55×10^{-6}	1.04×10^{-17}	Average size taken from Fig 2; range of radii taken from Fig S1: 5 to 150 μm.	$8.48\times10^{-18} – 7.70\times10^{-15}$
PE pristine, 10 mg/L carbamazepine [45]	5×10^{-10}	1.13×10^{-4}	5.94×10^{-16}	Average size taken from Fig 2; range of radii taken from Fig S1: 5 to 250 μm.	$1.16\times10^{-18} – 2.92\times10^{-15}$
PET pristine, 10 mg/L carbamazepine [45]	5×10^{-10}	1.06×10^{-4}	3.15×10^{-15}	Average size taken from Fig 2; range of radii taken from Fig S1: 50 to 250 μm.	$6.95\times10^{-16} – 1.75\times10^{-14}$
PU pristine, 10 mg/L Cu [46]	7×10^{-10} [S1]	5×10^{-5}	2.58×10^{-17}	Most particles have radius in range 50 μm to 100 μm, both pristine and aged.	$2.58\times10^{-17} – 1.03\times10^{-16}$
PU aged, 10 mg/L Cu [46]	7×10^{-10} [S1]	5×10^{-5}	3.30×10^{-16}		$3.30\times10^{-16} – 1.32\times10^{-15}$
PU pristine, 10 mg/L oxytetracycline [46]	5.8×10^{-10} (assumed same as tetracycline[S2])	5×10^{-5}	1.76×10^{-17}		$1.76\times10^{-17} – 7.02\times10^{-17}$
PU aged, 10 mg/L oxytetracycline [46]	5.8×10^{-10} (assumed same as tetracycline[S2])	5×10^{-5}	1.31×10^{-18}		$1.30\times10^{-18} – 5.24\times10^{-18}$
PP pristine, 10 mg/L oxytetracycline [47]	5.8×10^{-10} (assumed same as tetracycline[S2])	1.775×10^{-4}	na	Reported radii range for pristine: 1.775×10^{-4} to 2.5×10^{-4} m; no information for aged, thus assume same as pristine.	-
PP aged, 10 mg/L oxytetracycline [47]	5.8×10^{-10} (assumed same as tetracycline[S2])	1.775×10^{-4}	6.56×10^{-28}		$6.56\times10^{-28} – 1.30\times10^{-27}$
PP pristine, 10 mg/L chloramphenicol [47]	6.6×10^{-10} [S2]	1.775×10^{-4}	4.77×10^{-15}		$4.77\times10^{-15} – 9.48\times10^{-15}$
PP aged, 10 mg/L chloramphenicol [47]	6.6×10^{-10} [S2]	1.775×10^{-4}	2.11×10^{-16}		$2.11\times10^{-16} – 4.18\times10^{-16}$
PP pristine, 10 mg/L sulfamethoxazole [47]	5×10^{-10} [S3]	1.775×10^{-4}	6.21×10^{-26}		$6.21\times10^{-26} – 1.23\times10^{-25}$
PP aged, 10 mg/L sulfamethoxazole [47]	5×10^{-10} [S3]	1.775×10^{-4}	8.21×10^{-16}		$8.21\times10^{-16} – 1.63\times10^{-15}$
PP pristine, 10 mg/L sulfamethazine [47]	5×10^{-10}	1.775×10^{-4}	7.28×10^{-16}		$7.28\times10^{-16} – 1.45\times10^{-15}$
PP aged, 10 mg/L sulfamethazine [47]	5×10^{-10}	1.775×10^{-4}	4.86×10^{-14}		$4.86\times10^{-14} – 9.68\times10^{-14}$
PP pristine, 10 mg/L enrofloxacin [47]	8.0×10^{-11} [S4]	1.775×10^{-4}	1.25×10^{-16}		$1.25\times10^{-16} – 2.48\times10^{-16}$
PP aged, 10 mg/L enrofloxacin [47]	8.0×10^{-11} [S4]	1.775×10^{-4}	1.15×10^{-16}		$1.15\times10^{-16} – 2.29\times10^{-16}$
PP pristine, 10 mg/L ciprofloxacin [47]	5×10^{-10}	1.775×10^{-4}	na		-
PP aged, 10 mg/L ciprofloxacin [47]	5×10^{-10}	1.775×10^{-4}	na		-
PP pristine, 10 mg/L ofloxacin [47]	3.4×10^{-10} [S4]	1.775×10^{-4}	na		-

System	$D_{X,w}$ (m^2 s^{-1})	a (m) used in calculations	$D_{X,p}$ computed (m^2 s^{-1})	Reported range of a (m)	Computed range of $D_{X,p}$ (m^2 s^{-1}) for range of a
PP aged, 10 mg/L ofloxacin [47]	3.4×10^{-10} (S4)	1.775×10^{-4}	2.16×10^{-15}		2.16×10^{-15} – 4.28×10^{-15}
PP pristine, 10 mg/L norfloxacin [47]	5×10^{-10}	1.775×10^{-4}	5.84×10^{-14}		5.84×10^{-14} – 1.16×10^{-13}
PP aged, 10 mg/L norfloxacin [47]	5×10^{-10}	1.775×10^{-4}	1.26×10^{-15}		1.26×10^{-15} – 2.51×10^{-15}
PP pristine, 10 mg/L sulfamerazine [47]	5×10^{-10}	1.775×10^{-4}	4.97×10^{-15}		4.97×10^{-15} – 9.87×10^{-15}
PP aged, 10 mg/L sulfamerazine [47]	5×10^{-10}	1.775×10^{-4}	5.68×10^{-16}		5.68×10^{-16} – 1.13×10^{-15}
PP pristine, 10 mg/L sulfathiazole [47]	5×10^{-10}	1.775×10^{-4}	4.47×10^{-15}		4.47×10^{-15} – 8.89×10^{-15}
PP aged, 10 mg/L sulfathiazole [47]	5×10^{-10}	1.775×10^{-4}	1.75×10^{-15}		1.75×10^{-15} – 3.52×10^{-15}
PP pristine, 10 mg/L tetracycline [47]	5.8×10^{-10} (S2)	1.775×10^{-4}	1.82×10^{-14}		1.82×10^{-14} – 3.62×10^{-14}
PP aged, 10 mg/L tetracycline [47]	5.8×10^{-10} (S2)	1.775×10^{-4}	5.82×10^{-16}		5.82×10^{-16} – 1.16×10^{-15}
PS pristine, 20 mg/L tetracycline [48]	5.8×10^{-10} (S2)	5×10^{-5}	1.60×10^{-16}	Reported radii for pristine in range 5×10^{-5} to 1.25×10^{-4} m; aged assumed to be the same.	1.60×10^{-16} – 1.00×10^{-15}
PS aged, 20 mg/L tetracycline [48]	5.8×10^{-10} (S2)	5×10^{-5}	3.64×10^{-16}		3.64×10^{-16} – 2.28×10^{-15}

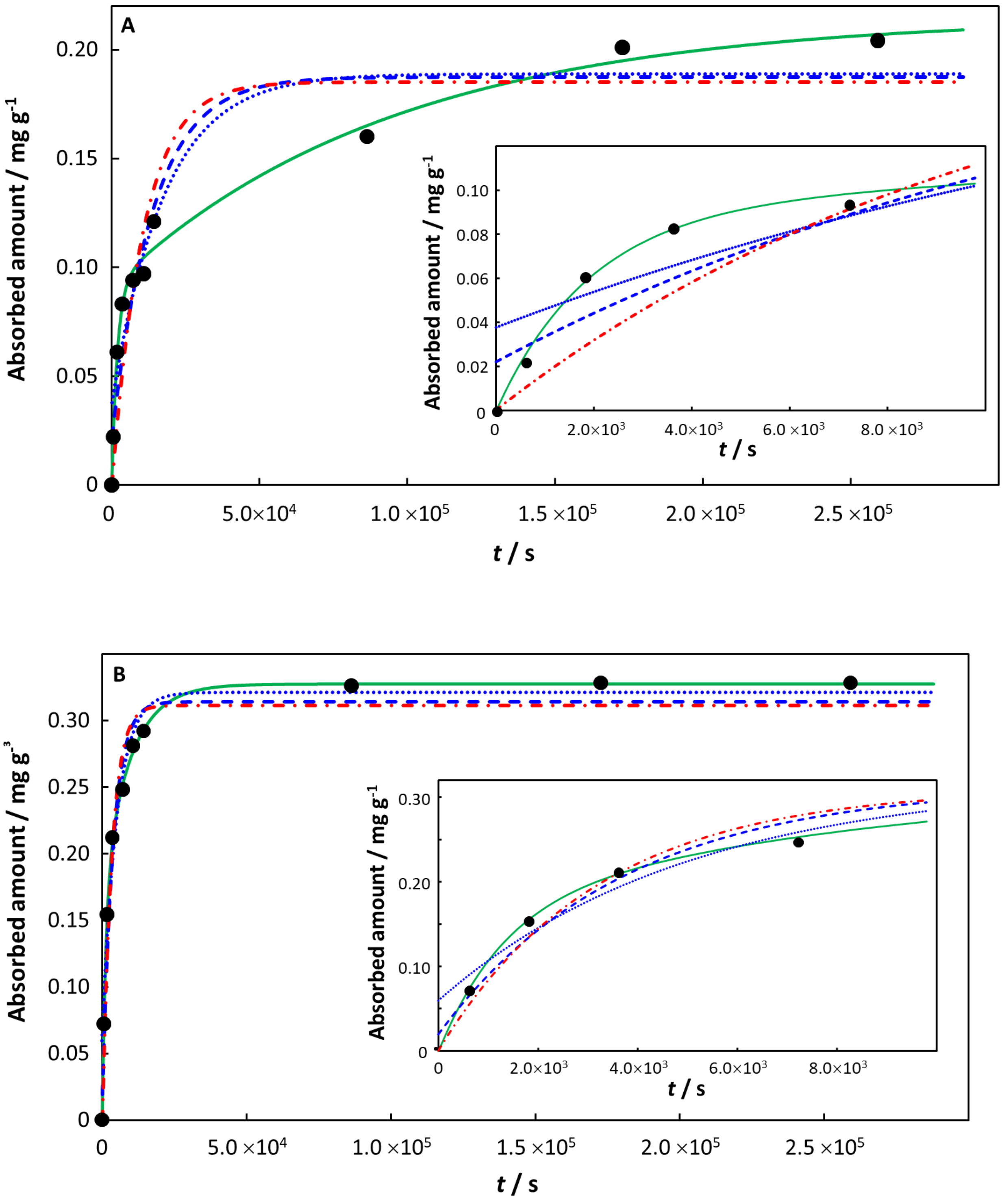

Figure S1. Absorption of sulfamethazine by **(A)** pristine PP and **(B)** aged PP, with insets showing data at short time. Experimental data (black solid circles) from Ref. [47] for for $c^*_{X,w}$ = 3.59×10^{-2} mol m^{-3} (10 mg dm^{-3}). Computed curves correspond to the involved integral fit, eq 18 (including the initial 0-absorption point: green solid curve), the mono-exponential, eq 21 (including the initial 0-absorption point: blue dashed line; excluding the initial 0-absorption point: blue dotted line) and the mono-exponential, eq 20 (including the initial 0-absorption point: red dot-dashed line). See main text for details of the fitting procedures.

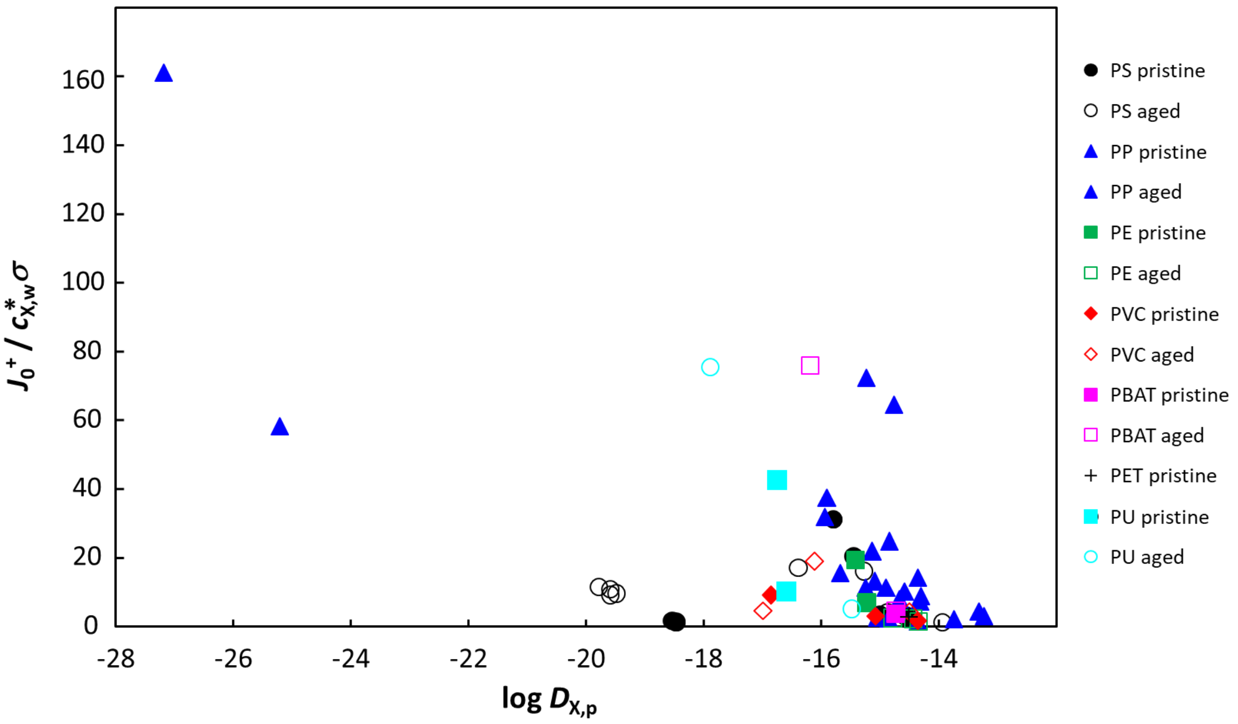

Figure S2. Dimensionless transient flux $J_0^+ / \left(c^*_{X,w}\sigma\right)$ as a function of log of the diffusion coefficient in the polymer, $D_{X,p}$ for all data sets that are well described by involved integral fitting (eq 18, including the 0-absorption point at t = 0).[40-48]

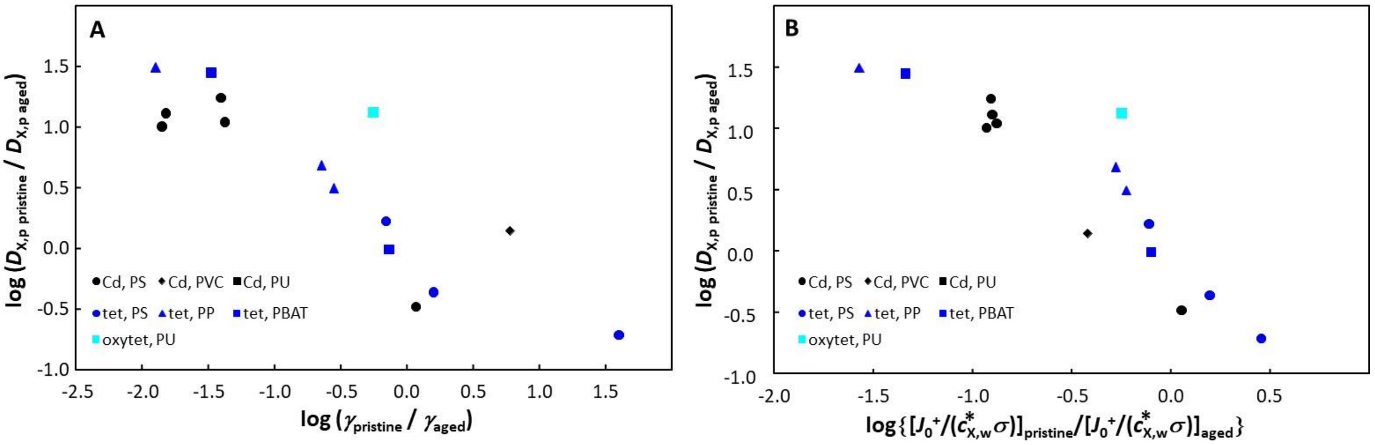

Figure S3. Log-log plot of **(A)** change in γ versus change in $D_{X,p}$ upon plastic particle aging and **(B)** change in the dimensionless transient flux term $J_0^+ / \left(c^*_{X,w}\sigma\right)$ versus change in $D_{X,p}$ upon plastic particle aging. Data are here provided for X = Cd(II),[40,44] tetracycline ("tet"),[41,47,48] and oxytetracycline ("oxytet").[46,47]

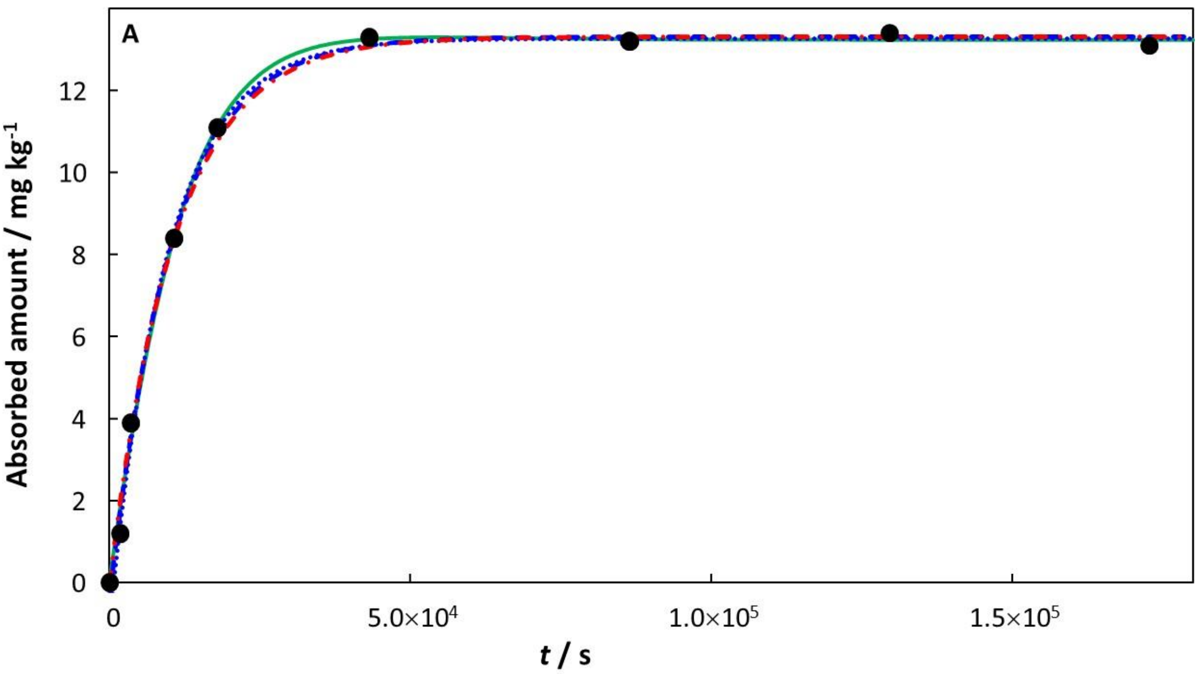
A
Absorbed amount / mg kg-1
t / s
0
2
4
6
8
10
12
5.0×10^4
1.0×10^5
1.5×10^5

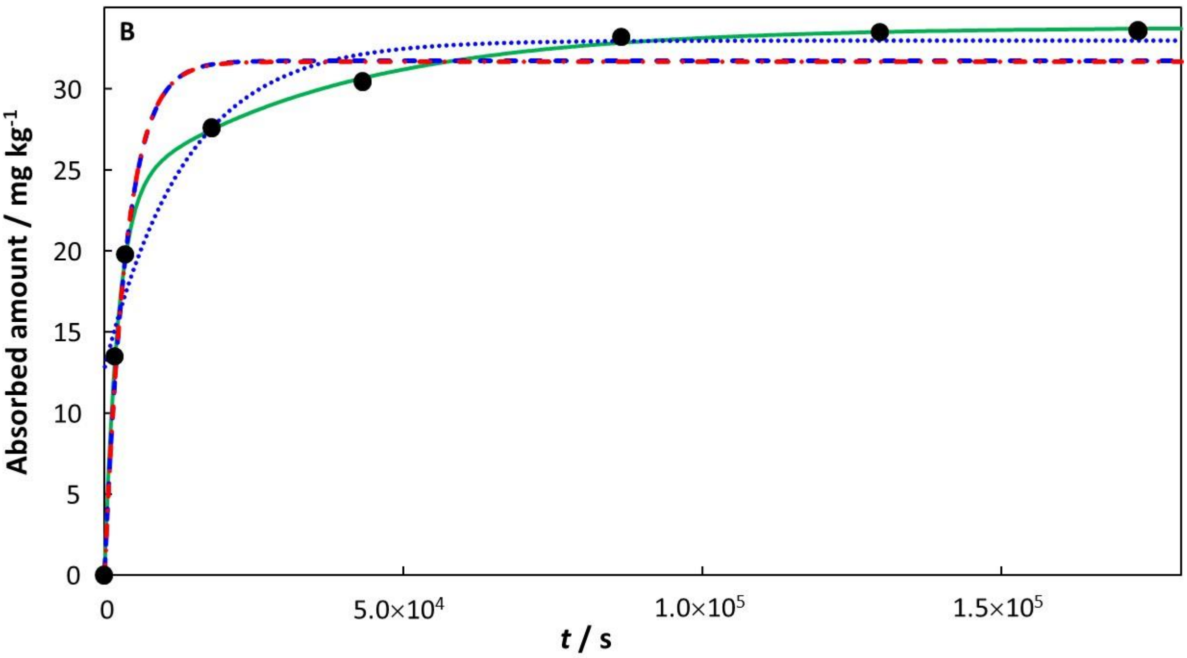
B
Absorbed amount / mg kg-1
t / s
0
5
10
15
20
25
30
5.0×10^4
1.0×10^5
1.5×10^5

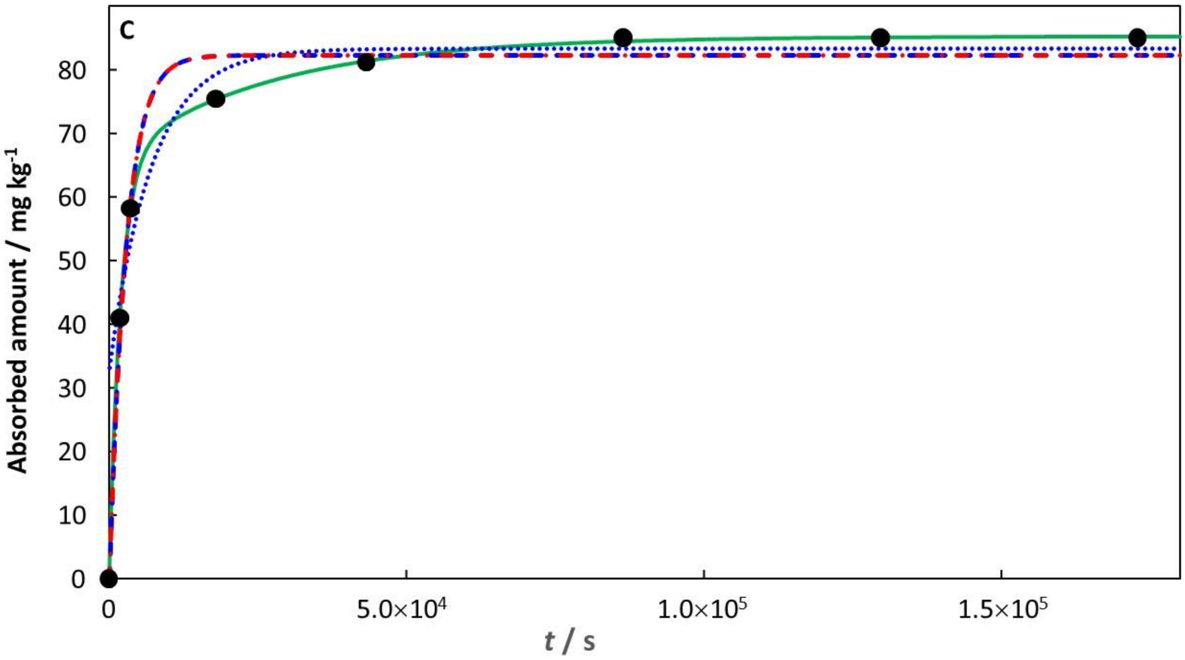

Figure S4. Absorption of Cd(II) by **(A)** pristine PS, **(B)** H_2O_2 aged PS, and **(C)** Fenton aged PS. Experimental data (black solid circles) from Ref. [40] for $c^*_{X,w}$ = 4.4×10^{-3} mol m^{-3} (0.5 ppm). Computed curves correspond to the involved integral fit, eq 18 (including the initial 0-absorption point: green solid curve), the mono-exponential, eq 21 (including the initial 0-absorption point: blue dashed line; excluding the initial 0-absorption point: blue dotted line) and the mono-exponential, eq 20 (including the initial 0-absorption point: red dot-dashed line). See main text for details of the fitting procedures.

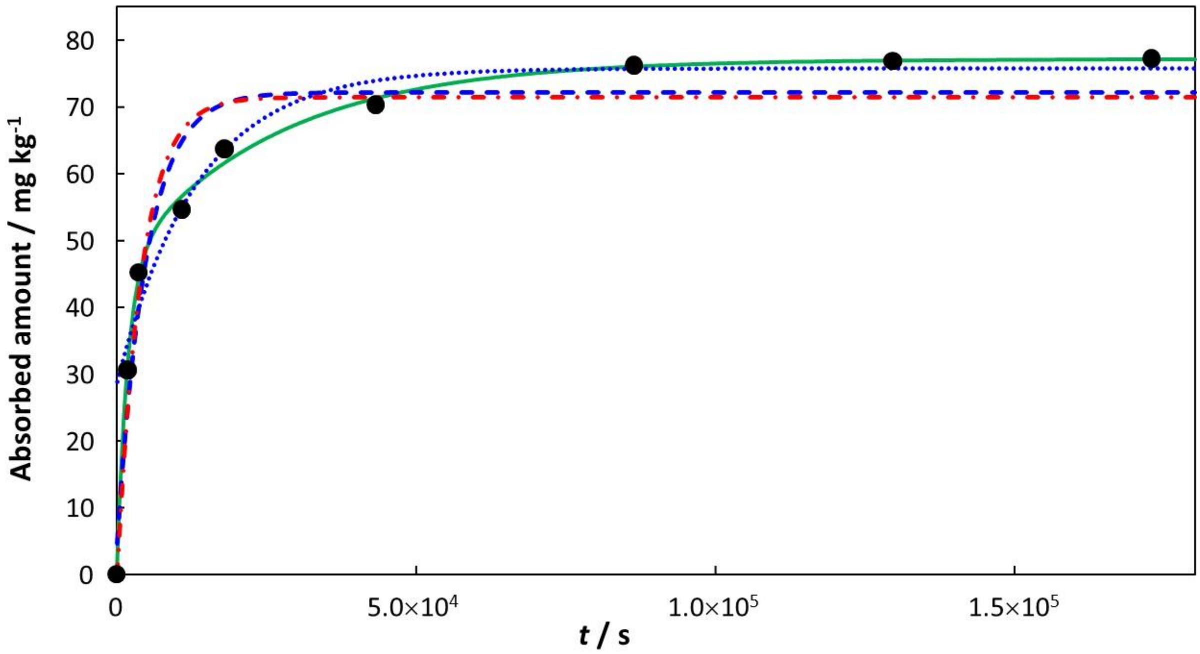

Figure S5. Absorption of Cd(II) by H_2O_2 aged PS. Experimental data (black solid circles) from Ref. [40] for $c^*_{X,w}$ = 1.8×10^{-2} mol m^{-3} (2 ppm). Corresponding curves for pristine PS and Fenton aged PS are given in the main text, Figure 2. Computed curves correspond to the involved integral fit, eq 18 (including the initial 0-absorption point: green solid curve), the mono-exponential, eq 21 (including the initial 0-absorption point: blue dashed line; excluding the initial 0-absorption point: blue dotted line) and the mono-exponential, eq 20 (including the initial 0-absorption point: red dot-dashed line). See main text for details of the fitting procedures.

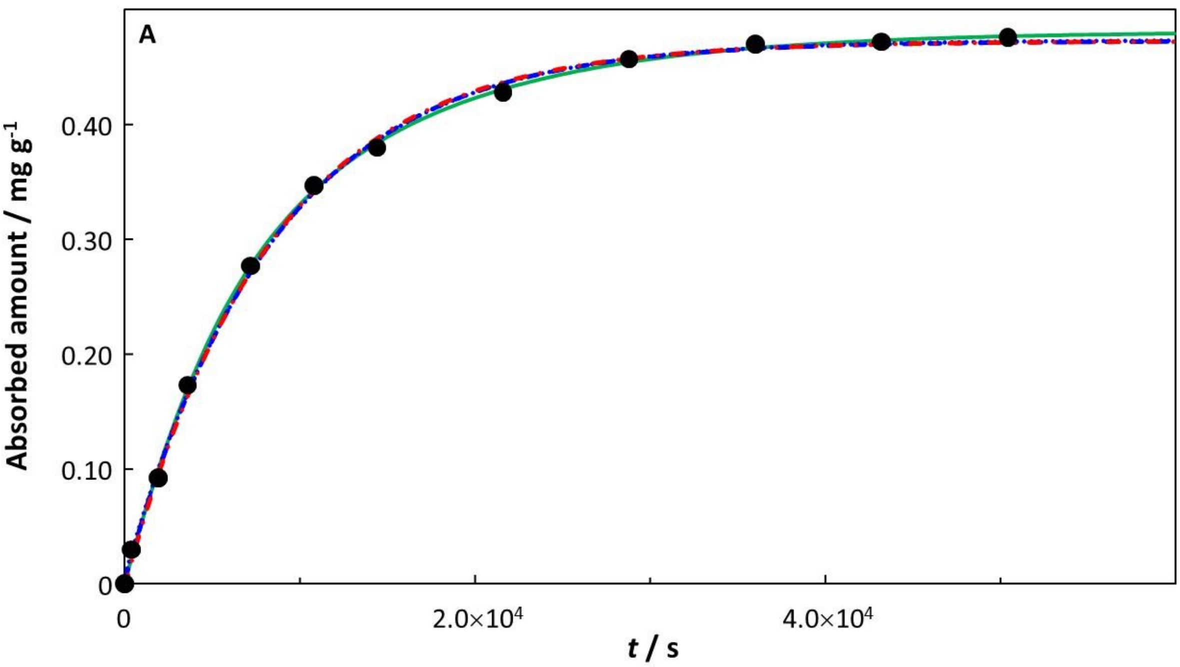
A
Absorbed amount / mg g-1
0.40
0.30
0.20
0.10
0
0
2.0×10^4
4.0×10^4
t / s

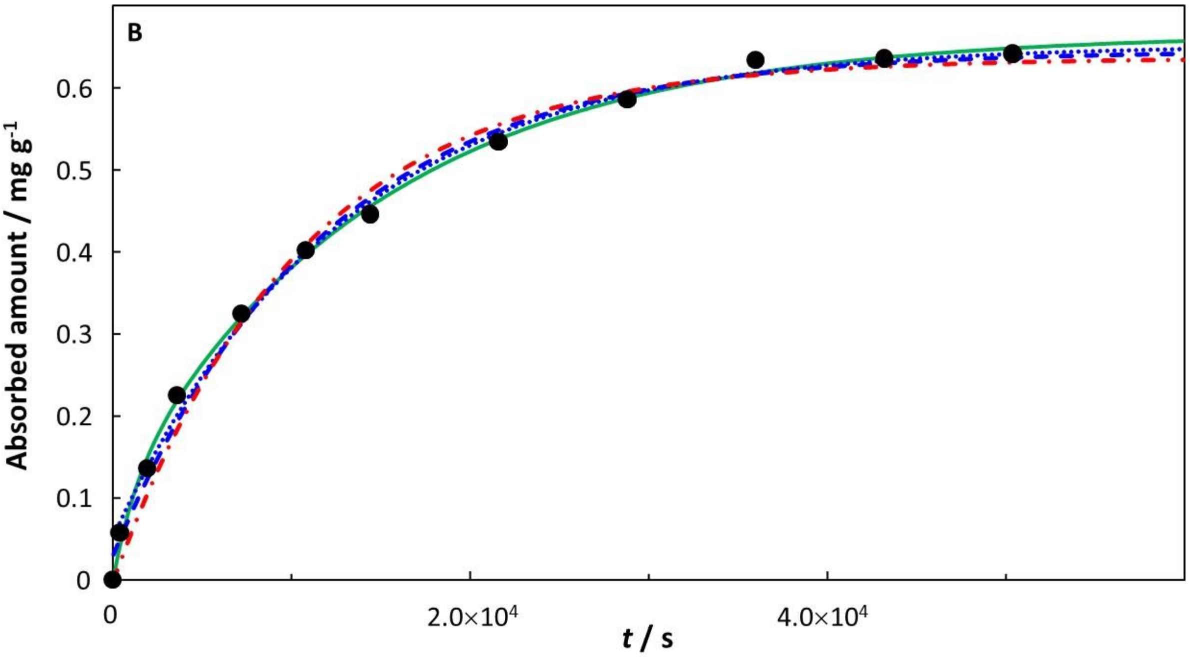
B
Absorbed amount / mg g-1
0.6
0.5
0.4
0.3
0.2
0.1
0
0
2.0×10^4
4.0×10^4
t / s

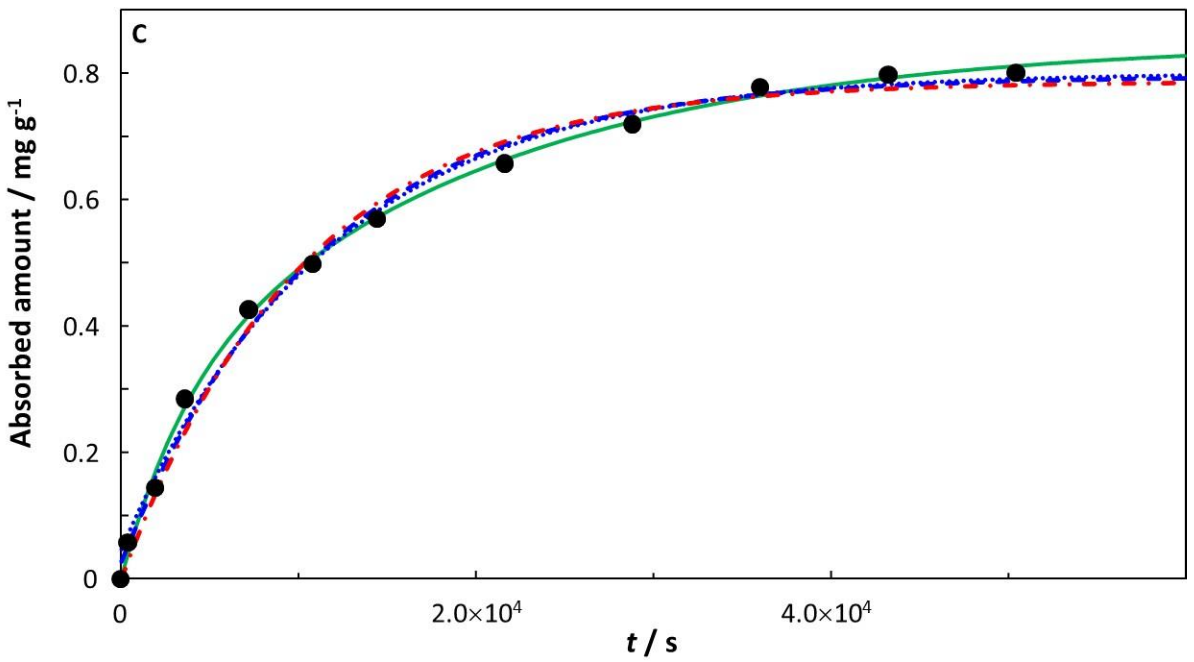

Figure S6. Absorption of tetracycline by **(A)** pristine PP, **(B)** UV aged PP, and **(C)** $K_2S_2O_8$ aged PP. Experimental data (black solid circles) from Ref. [41] for $c^*_{X,w}$ = 2.25×10^{-2} mol m^{-3} (10 mg dm^{-3}). Computed curves correspond to the involved integral fit, eq 18 (including the initial 0-absorption point: green solid curve), the mono-exponential, eq 21 (including the initial 0-absorption point: blue dashed line; excluding the initial 0-absorption point: blue dotted line) and the mono-exponential, eq 20 (including the initial 0-absorption point: red dot-dashed line). See main text for details of the fitting procedures.

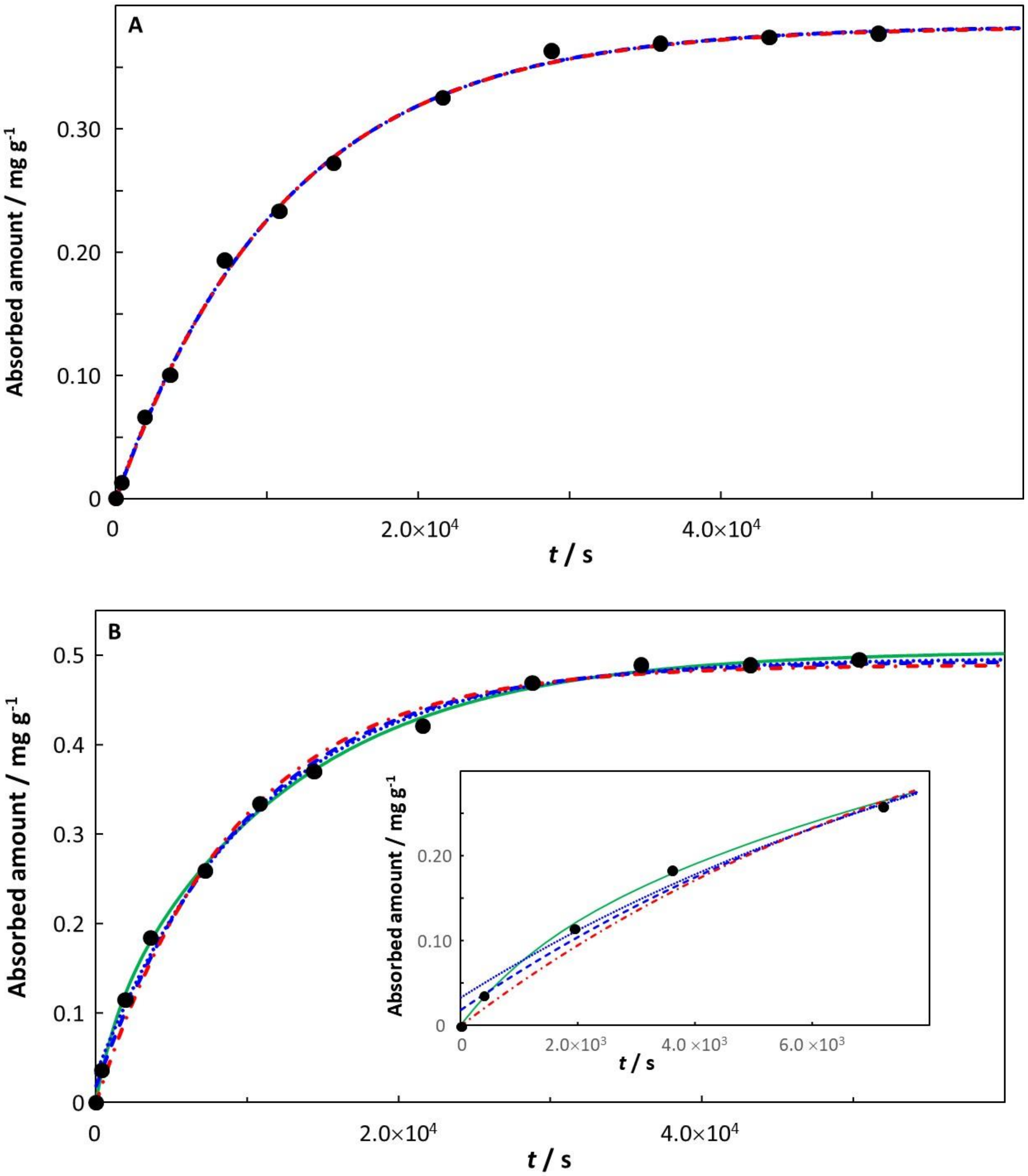
A
Absorbed amount / mg g-1
0.30
0.20
0.10
0
0
2.0×10^4
4.0×10^4
t / s
B
Absorbed amount / mg g-1
0.5
0.4
0.3
0.2
0.1
0
0
2.0×10^4
4.0×10^4
t / s
0.20
0.10
0
2.0×10^3
4.0×10^3
6.0×10^3

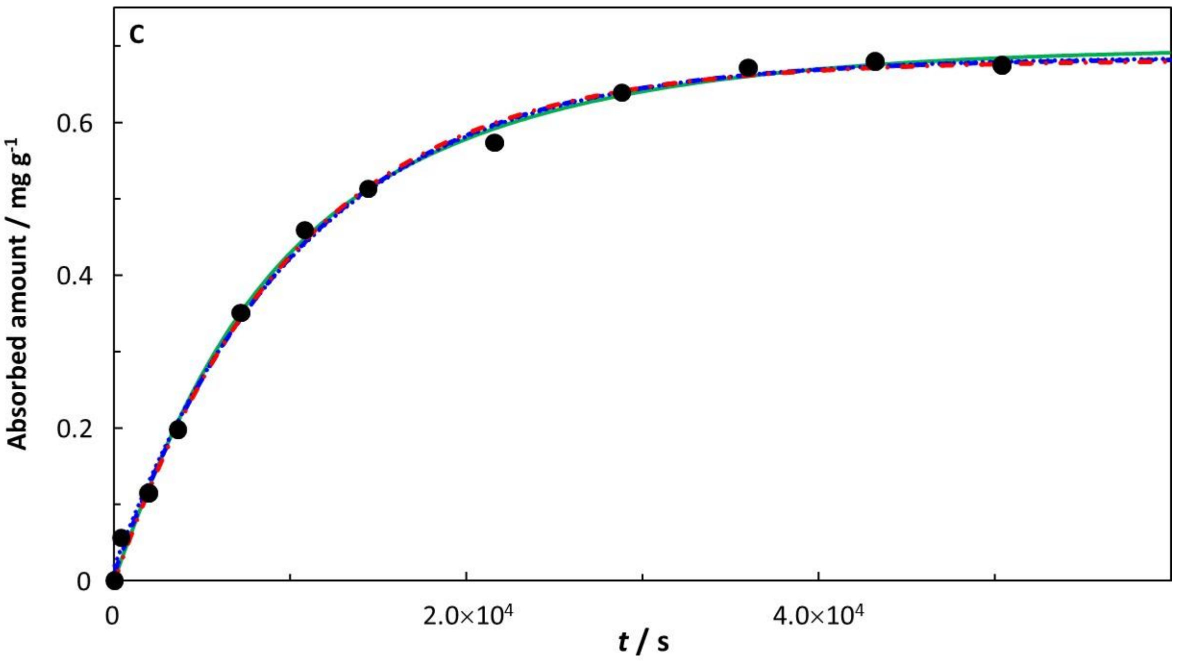

Figure S7. Absorption of tetracycline by **(A)** pristine PE, **(B)** UV aged PE with inset showing data at short times, and **(C)** $K_2S_2O_8$ aged PE. Experimental data (black solid circles) from Ref. [41] for $c^{*}_{\mathrm{X,w}}$ = 2.25×10^{-2} mol m^{-3} (10 mg dm^{-3}). Computed curves correspond to the involved integral fit, eq 18 (including the initial 0-absorption point: green solid curve), the mono-exponential, eq 21 (including the initial 0-absorption point: blue dashed line; excluding the initial 0-absorption point: blue dotted line) and the mono-exponential, eq 20 (including the initial 0-absorption point: red dot-dashed line). See main text for details of the fitting procedures.

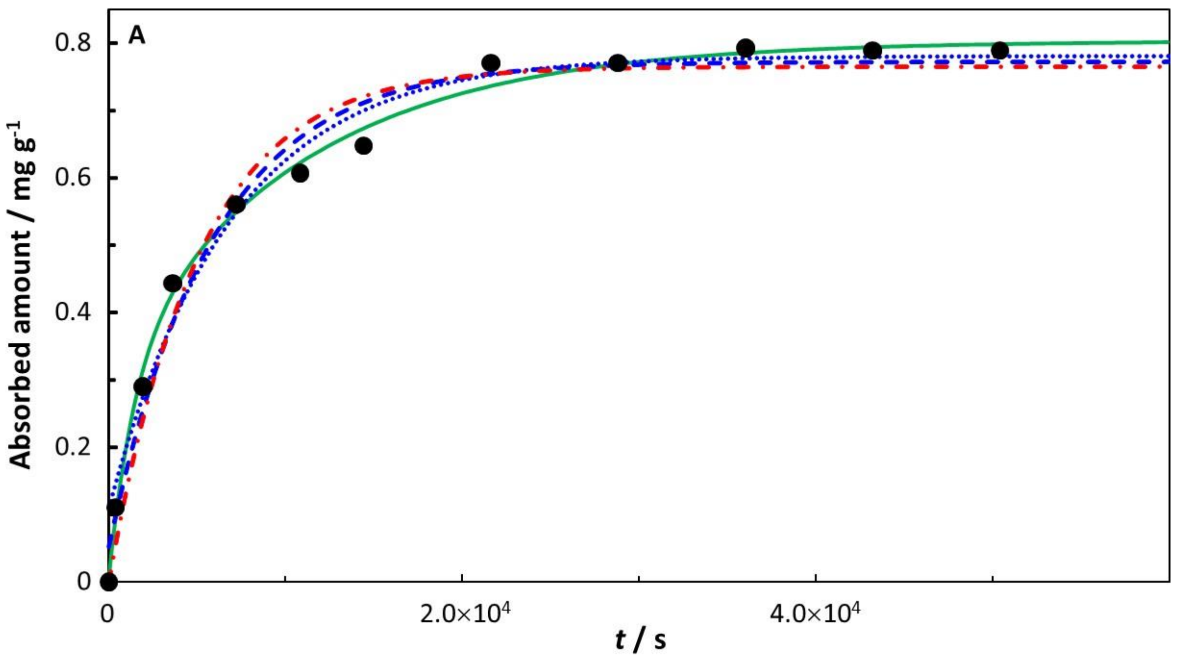
A
0.8
0.6
0.4
0.2
0
Absorbed amount / mg g^{-1}
0
2.0×10^4
4.0×10^4
t / s

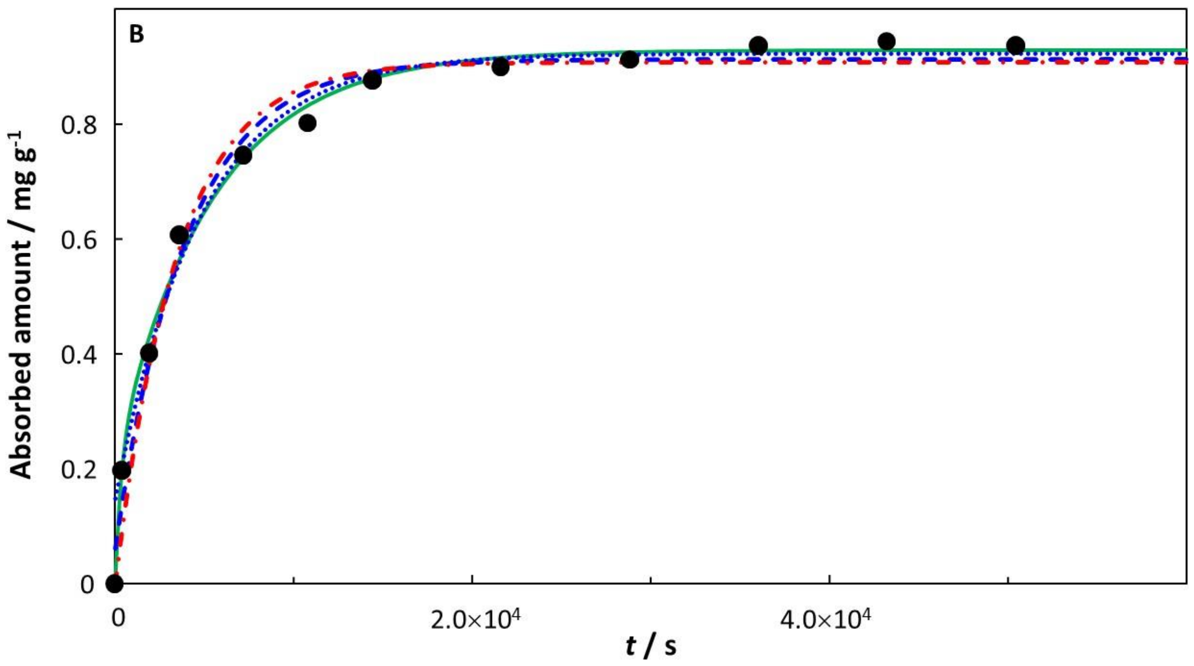
B
0.8
0.6
0.4
0.2
0
Absorbed amount / mg g^{-1}
0
2.0×10^4
4.0×10^4
t / s

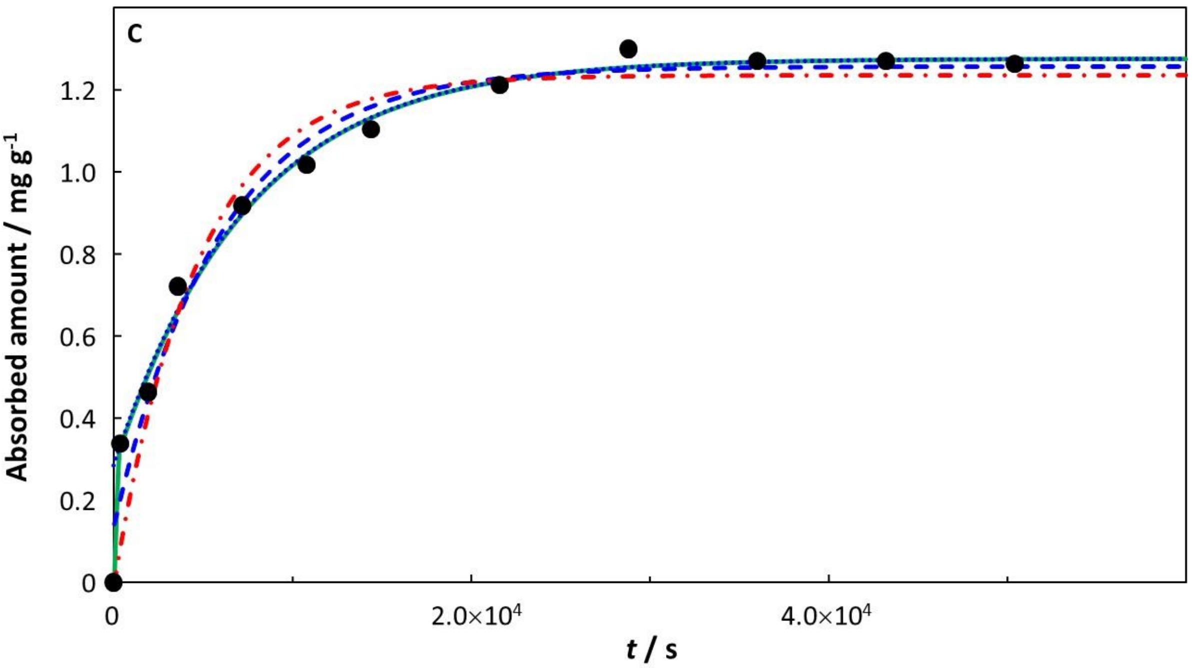

Figure S8. Absorption of tetracycline by **(A)** pristine PBAT, **(B)** UV aged PBAT, and **(C)** $K_2S_2O_8$ aged PBAT. Experimental data (black solid circles) from Ref. [41] for $c^{*}_{X,w}$ = 2.25×10^{-2} mol m^{-3} (10 mg dm^{-3}). Computed curves correspond to the involved integral fit, eq 18 (including the initial 0-absorption point: green solid curve), the mono-exponential, eq 21 (including the initial 0-absorption point: blue dashed line; excluding the initial 0-absorption point: blue dotted line) and the mono-exponential, eq 20 (including the initial 0-absorption point: red dot-dashed line). See main text for details of the fitting procedures.

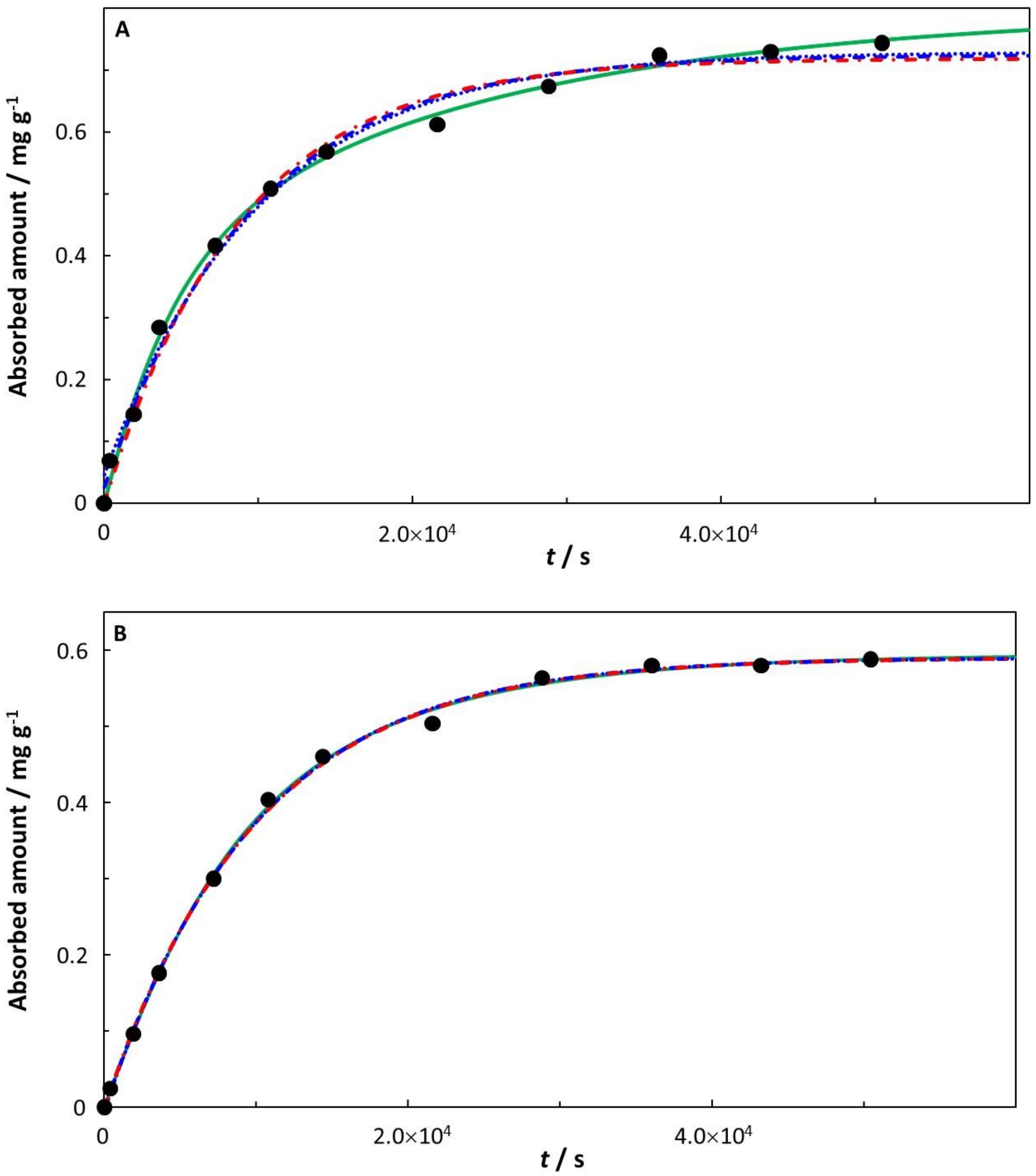
A
B
Absorbed amount / mg g-1
t / s
0
0.2
0.4
0.6
2.0×10^4
4.0×10^4

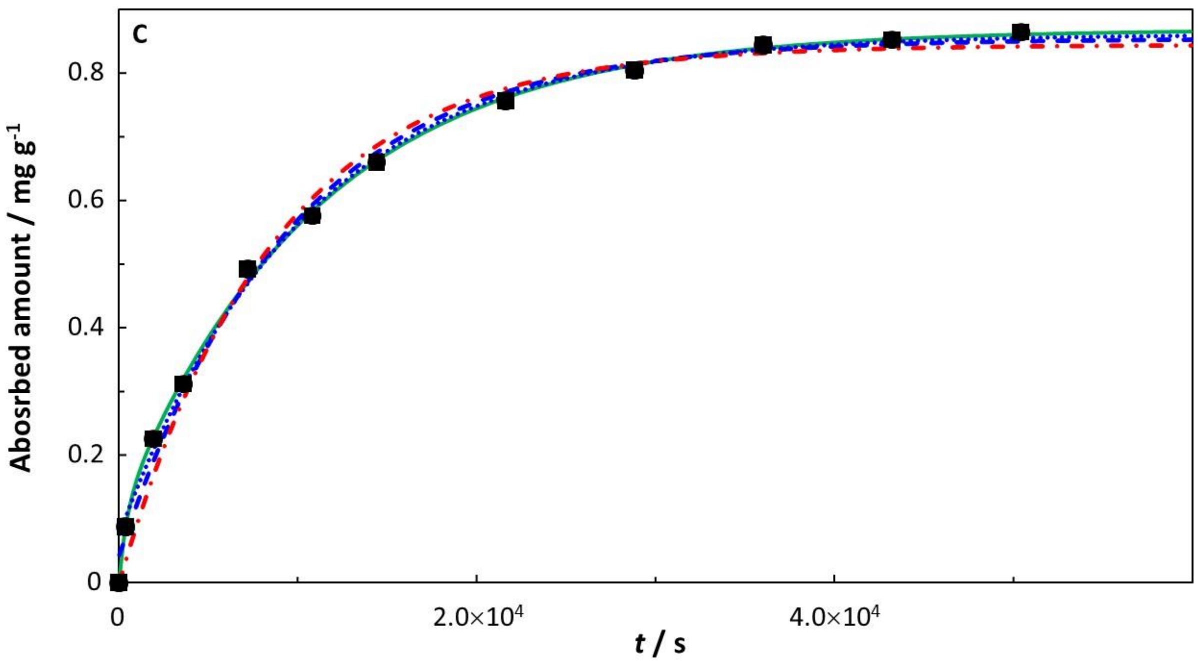

Figure S9. Absorption of tetracycline by **(A)** pristine PS, **(B)** UV aged PS, and **(C)** $K_2S_2O_8$ aged PS. Experimental data (black solid circles) from Ref. [41] for $c^*_{X,w}$ = 2.25×10^{-2} mol m^{-3} (10 mg dm^{-3}). Computed curves correspond to the involved integral fit, eq 18 (including the initial 0-absorption point: green solid curve), the mono-exponential, eq 21 (including the initial 0-absorption point: blue dashed line; excluding the initial 0-absorption point: blue dotted line) and the mono-exponential, eq 20 (including the initial 0-absorption point: red dot-dashed line). See main text for details of the fitting procedures.

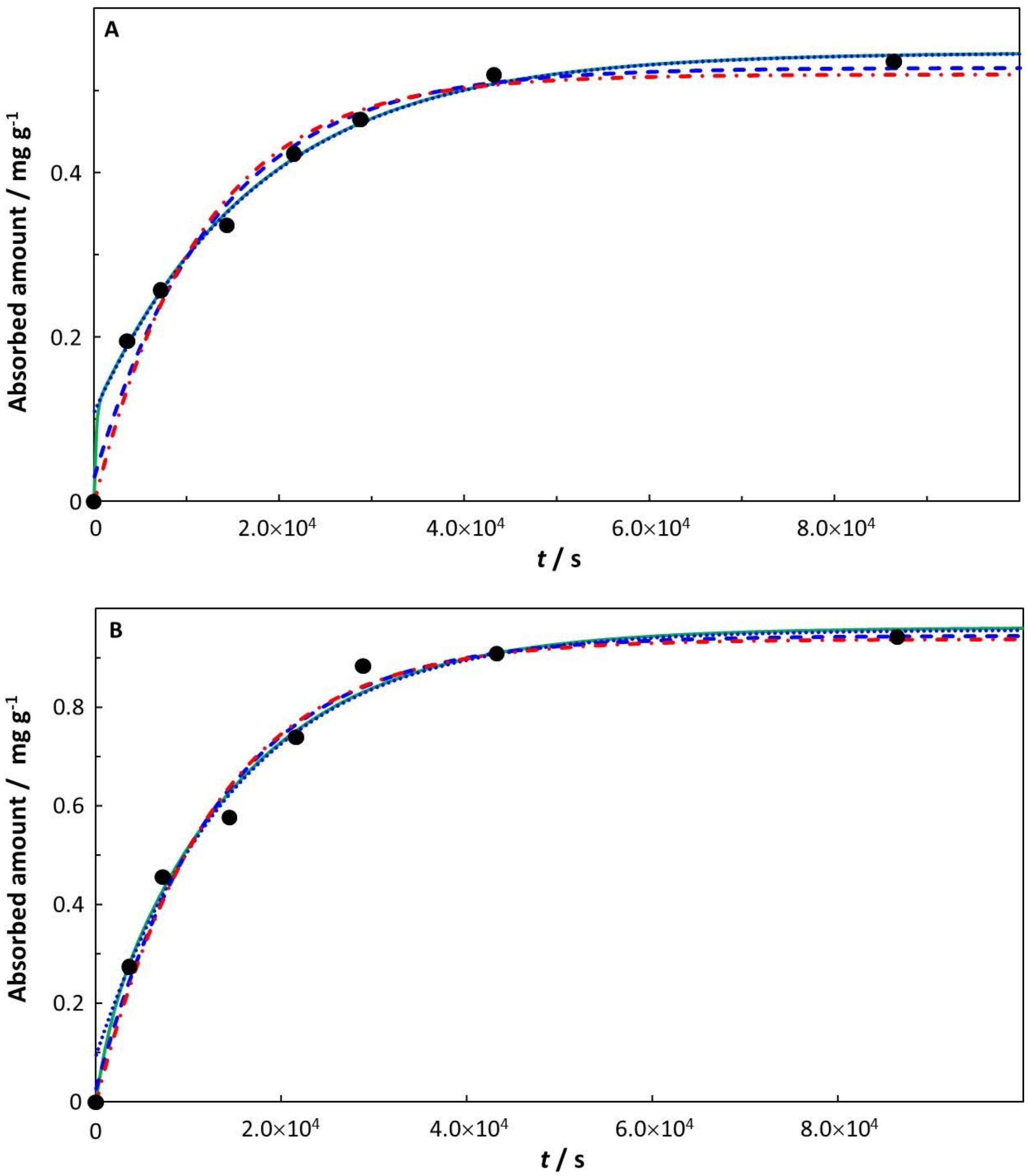

Figure S10. Absorption of atrazine by **(A)** pristine PE, and **(B)** aged PE. Experimental data (black solid circles) from Ref. [42] for $c^*_{\mathrm{X,w}}$ = 2.32×10^{-2} mol m^{-3} (5 mg dm^{-3}). Computed curves correspond to the involved integral fit, eq 18 (including the initial 0-absorption point: green solid curve), the mono-exponential, eq 21 (including the initial 0-absorption point: blue dashed line; excluding the initial 0-absorption point: blue dotted line) and the mono-exponential, eq 20 (including the initial 0-absorption point: red dot-dashed line). See main text for details of the fitting procedures.

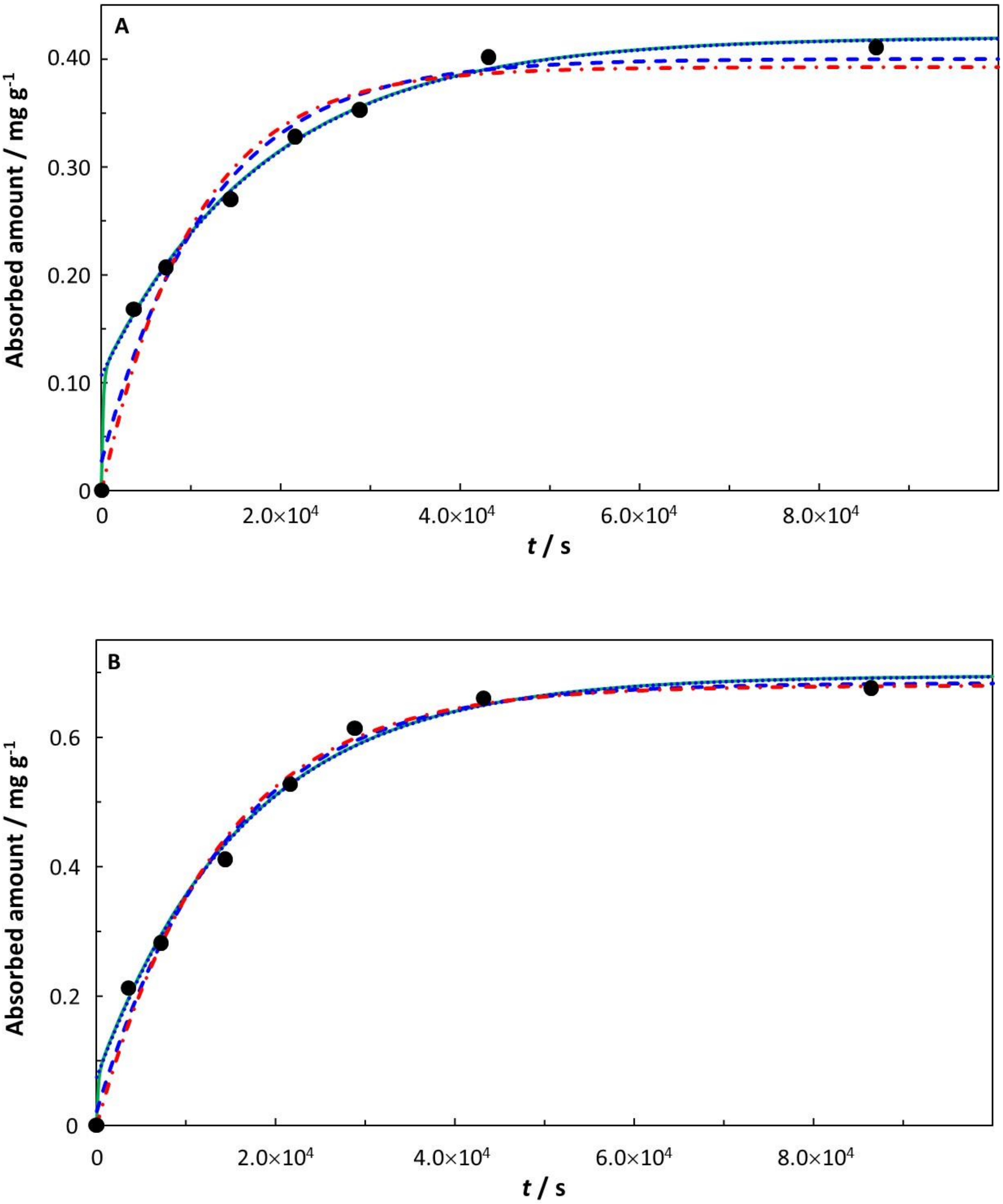

Figure S11. Absorption of atrazine by **(A)** pristine PP, and **(B)** aged PP. Experimental data (black solid circles) from Ref. [42] for $c^*_{\mathrm{X,w}}$ = 2.32×10^{-2} mol m^{-3} (5 mg dm^{-3}). Computed curves correspond to the involved integral fit, eq 18 (including the initial 0-absorption point: green solid curve), the mono-exponential, eq 21 (including the initial 0-absorption point: blue dashed line; excluding the initial 0-absorption point: blue dotted line) and the mono-exponential, eq 20 (including the initial 0-absorption point: red dot-dashed line). See main text for details of the fitting procedures.

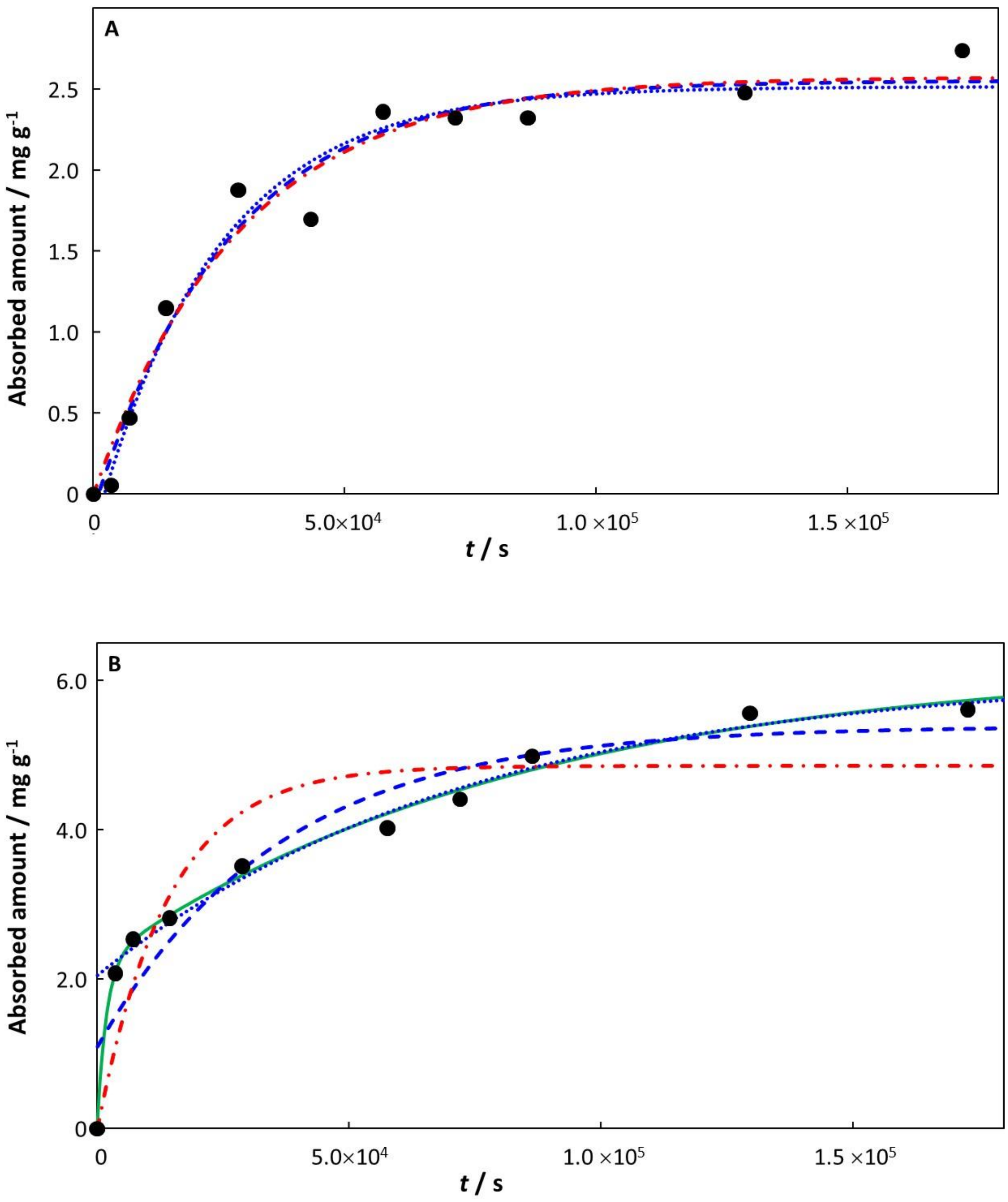

Figure S12. Absorption of ciprofloxacin by **(A)** pristine PS, and **(B)** aged PS. Experimental data (black solid circles) from Ref. [43] for $c^{*}_{\mathrm{X,w}}$ = 3.02×10^{-2} mol m^{-3} (10 mg dm^{-3}). Computed curves correspond to the involved integral fit, eq 18 (including the initial 0-absorption point: green solid curve), the mono-exponential, eq 21 (including the initial 0-absorption point: blue dashed line; excluding the initial 0-absorption point: blue dotted line) and the mono-exponential, eq 20 (including the initial 0-absorption point: red dot-dashed line). See main text for details of the fitting procedures.

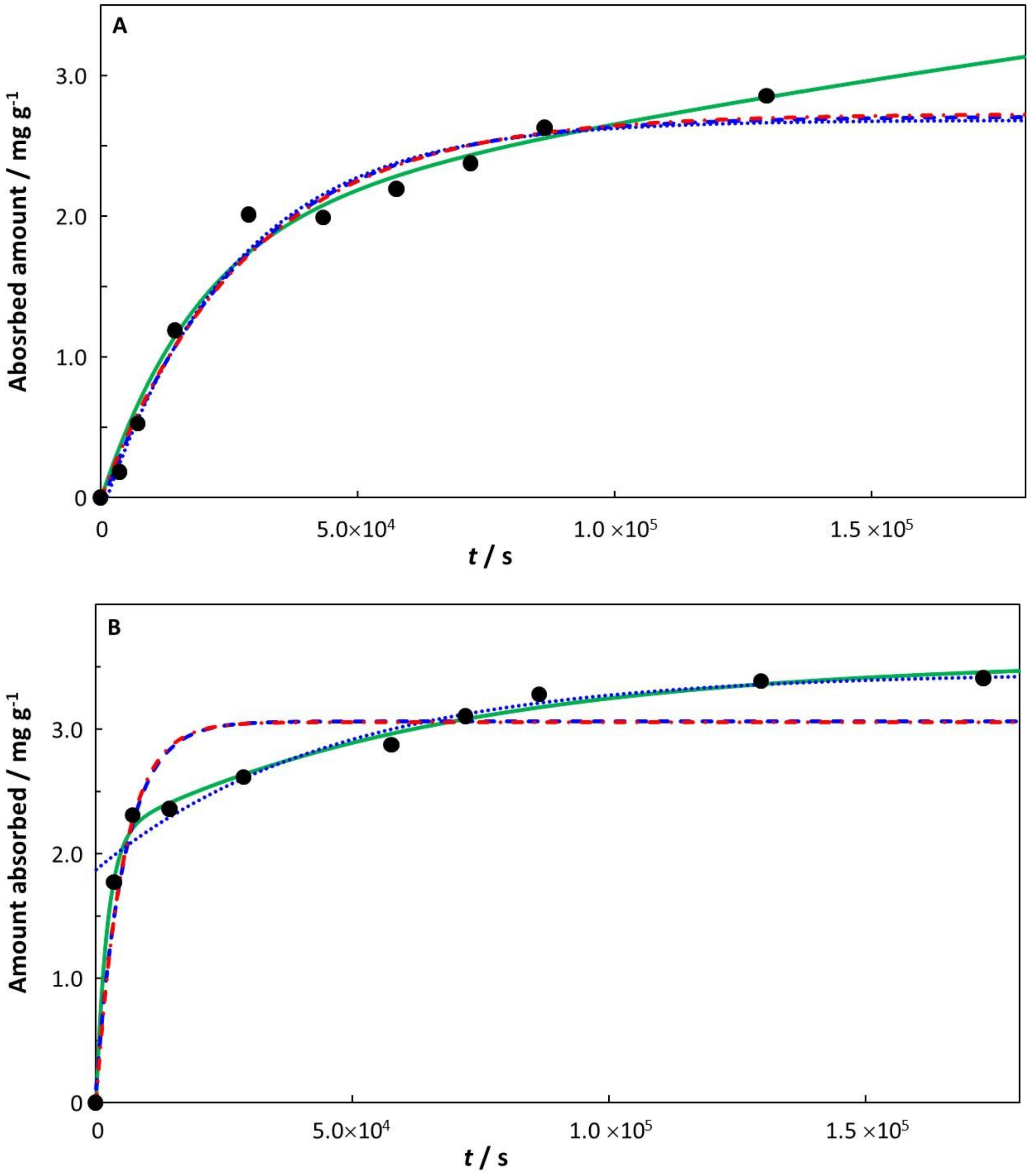

Figure S13. Absorption of ciprofloxacin by **(A)** pristine PVC, and **(B)** aged PVC. Experimental data (black solid circles) from Ref. [43] for $c^{*}_{\mathrm{X,w}}$ = 3.02×10^{-2} mol m^{-3} (10 mg dm^{-3}). Computed curves correspond to the involved integral fit, eq 18 (including the initial 0-absorption point: green solid curve), the mono-exponential, eq 21 (including the initial 0-absorption point: blue dashed line; excluding the initial 0-absorption point: blue dotted line) and the mono-exponential, eq 20 (including the initial 0-absorption point: red dot-dashed line). See main text for details of the fitting procedures.

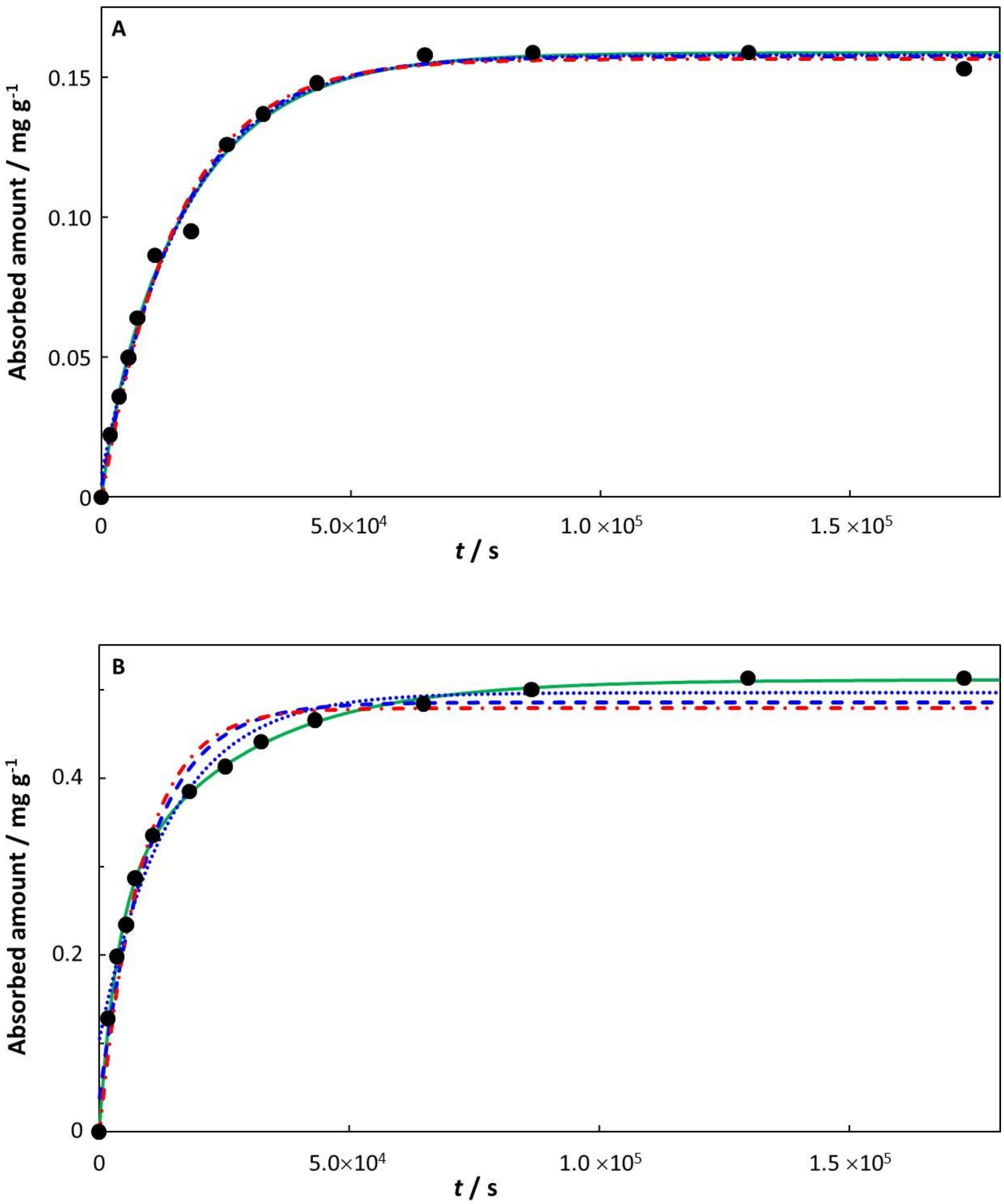

Figure S14. Absorption of Cd(II) by **(A)** pristine PVC, and **(B)** aged PVC. Experimental data (black solid circles) from Ref. [44] for $c^*_{\mathrm{X,w}}$ = 8.9×10^{-3} mol m^{-3} (1 mg dm^{-3}). Computed curves correspond to the involved integral fit, eq 18 (including the initial 0-absorption point: green solid curve), the mono-exponential, eq 21 (including the initial 0-absorption point: blue dashed line; excluding the initial 0-absorption point: blue dotted line) and the mono-exponential, eq 20 (including the initial 0-absorption point: red dot-dashed line). See main text for details of the fitting procedures.

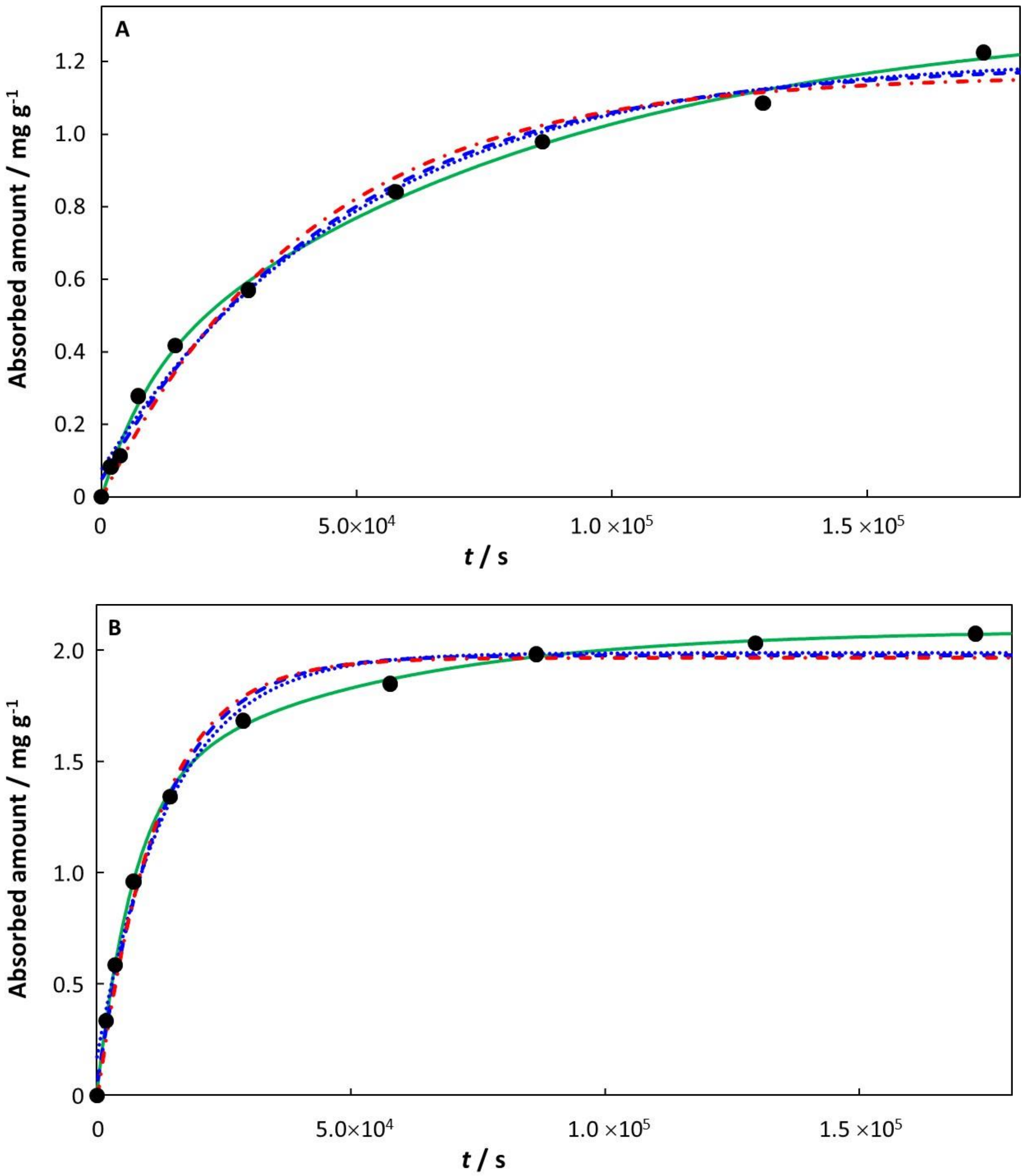

Figure S15. Absorption of carbamazepine by **(A)** pristine PVC, and **(B)** aged PVC. Experimental data (black solid circles) from Ref. [45] for $c^*_{\mathrm{X,w}}$ = 4.23×10^{-2} mol m^{-3} (10 mg dm^{-3}). Computed curves correspond to the involved integral fit, eq 18 (including the initial 0-absorption point: green solid curve), the mono-exponential, eq 21 (including the initial 0-absorption point: blue dashed line; excluding the initial 0-absorption point: blue dotted line) and the mono-exponential, eq 20 (including the initial 0-absorption point: red dot-dashed line). See main text for details of the fitting procedures.

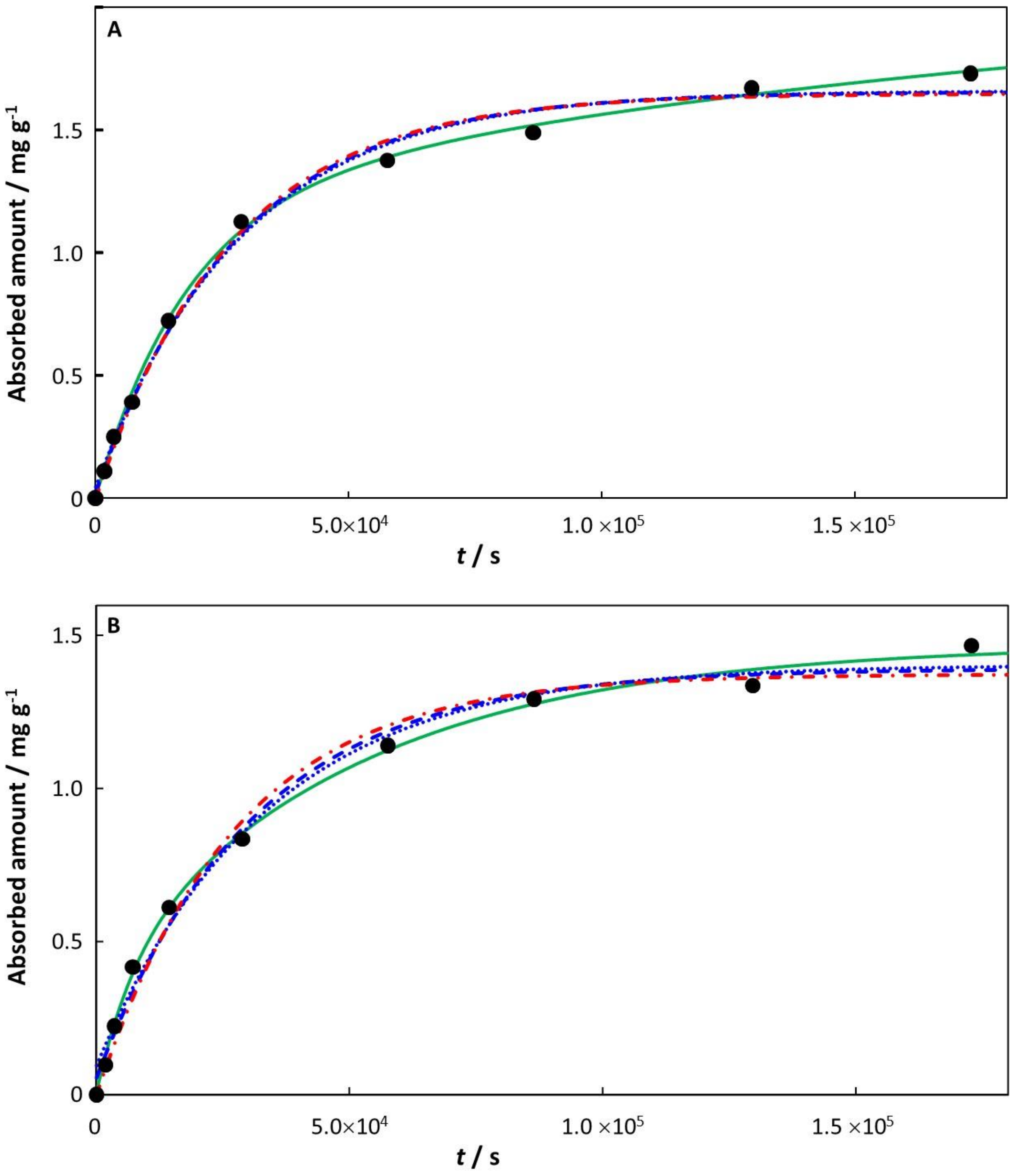

Figure S16. Absorption of carbamazepine by **(A)** pristine PE, and **(B)** pristine PET. Experimental data (black solid circles) from Ref. [45] for $c^*_{\mathrm{X,w}}$ = 4.23×10^{-2} mol m^{-3} (10 mg dm^{-3}). Computed curves correspond to the involved integral fit, eq 18 (including the initial 0-absorption point: green solid curve), the mono-exponential, eq 21 (including the initial 0-absorption point: blue dashed line; excluding the initial 0-absorption point: blue dotted line) and the mono-exponential, eq 20 (including the initial 0-absorption point: red dot-dashed line). See main text for details of the fitting procedures.

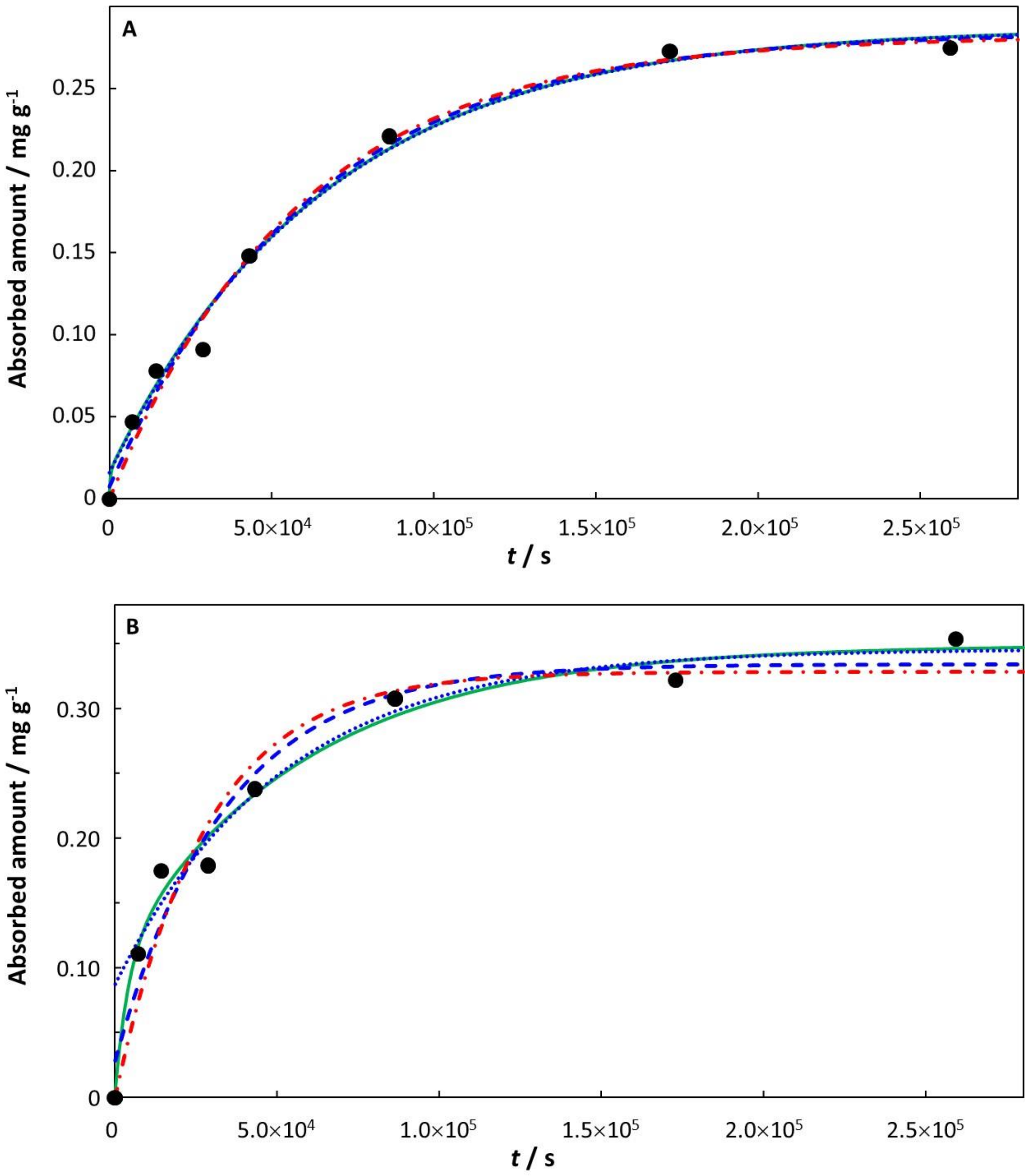

Figure S17. Absorption of Cu(II) by **(A)** pristine PU, and **(B)** aged PU. Experimental data (black solid circles) from Ref. [46] for $c^*_{\mathrm{X,w}}$ = 0.16 mol m^{-3} (10 mg dm^{-3}). Computed curves correspond to the involved integral fit, eq 18 (including the initial 0-absorption point: green solid curve), the mono-exponential, eq 21 (including the initial 0-absorption point: blue dashed line; excluding the initial 0-absorption point: blue dotted line) and the mono-exponential, eq 20 (including the initial 0-absorption point: red dot-dashed line). See main text for details of the fitting procedures.

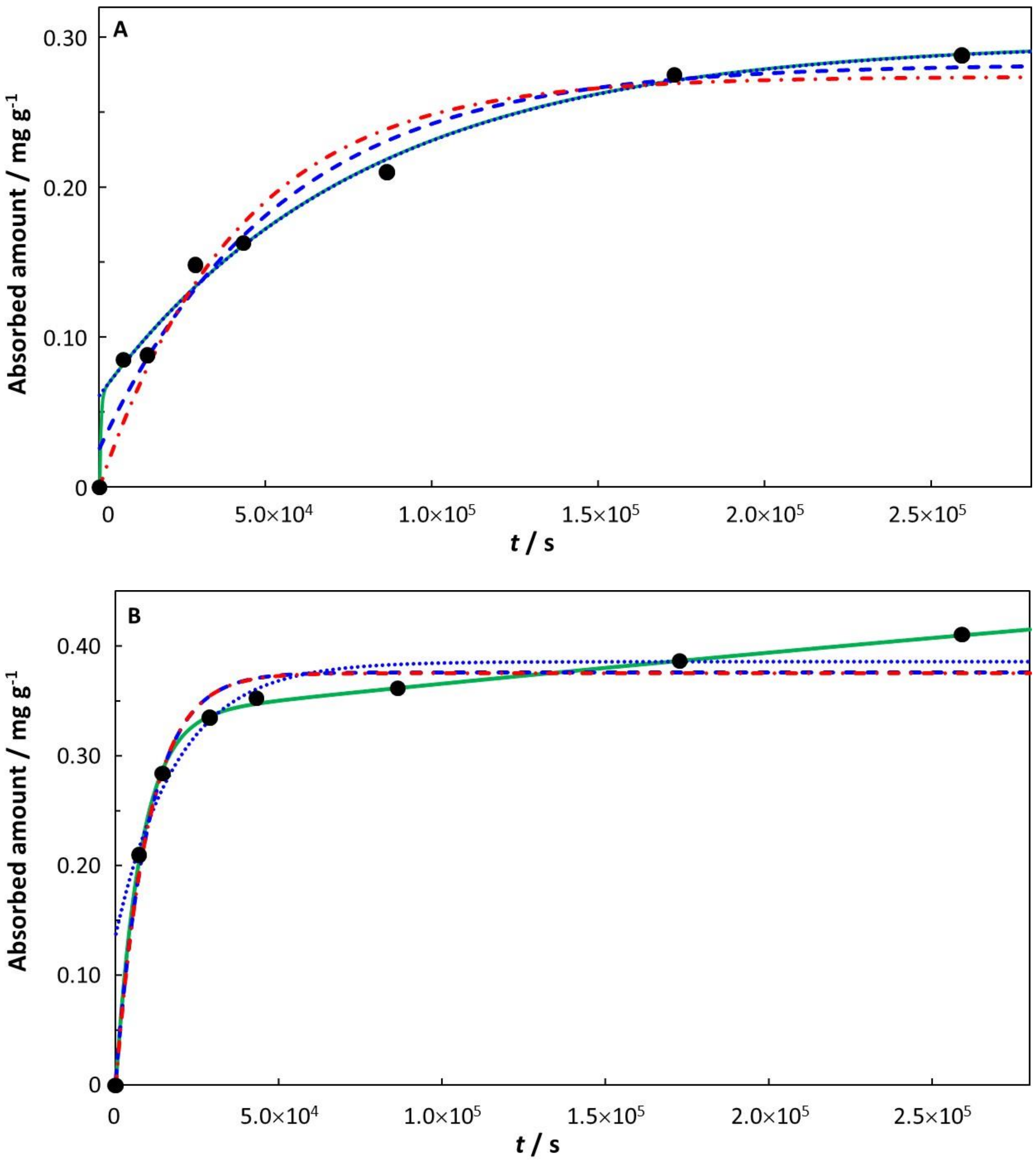

Figure S18. Absorption of oxytetracycline by **(A)** pristine PU, and **(B)** aged PU. Experimental data (black solid circles) from Ref. [46] for $c^*_{\mathrm{X,w}}$ = 2.17×10^{-2} mol m^{-3} (10 mg dm^{-3}). Computed curves correspond to the involved integral fit, eq 18 (including the initial 0-absorption point: green solid curve), the mono-exponential, eq 21 (including the initial 0-absorption point: blue dashed line; excluding the initial 0-absorption point: blue dotted line) and the mono-exponential, eq 20 (including the initial 0-absorption point: red dot-dashed line). See main text for details of the fitting procedures.

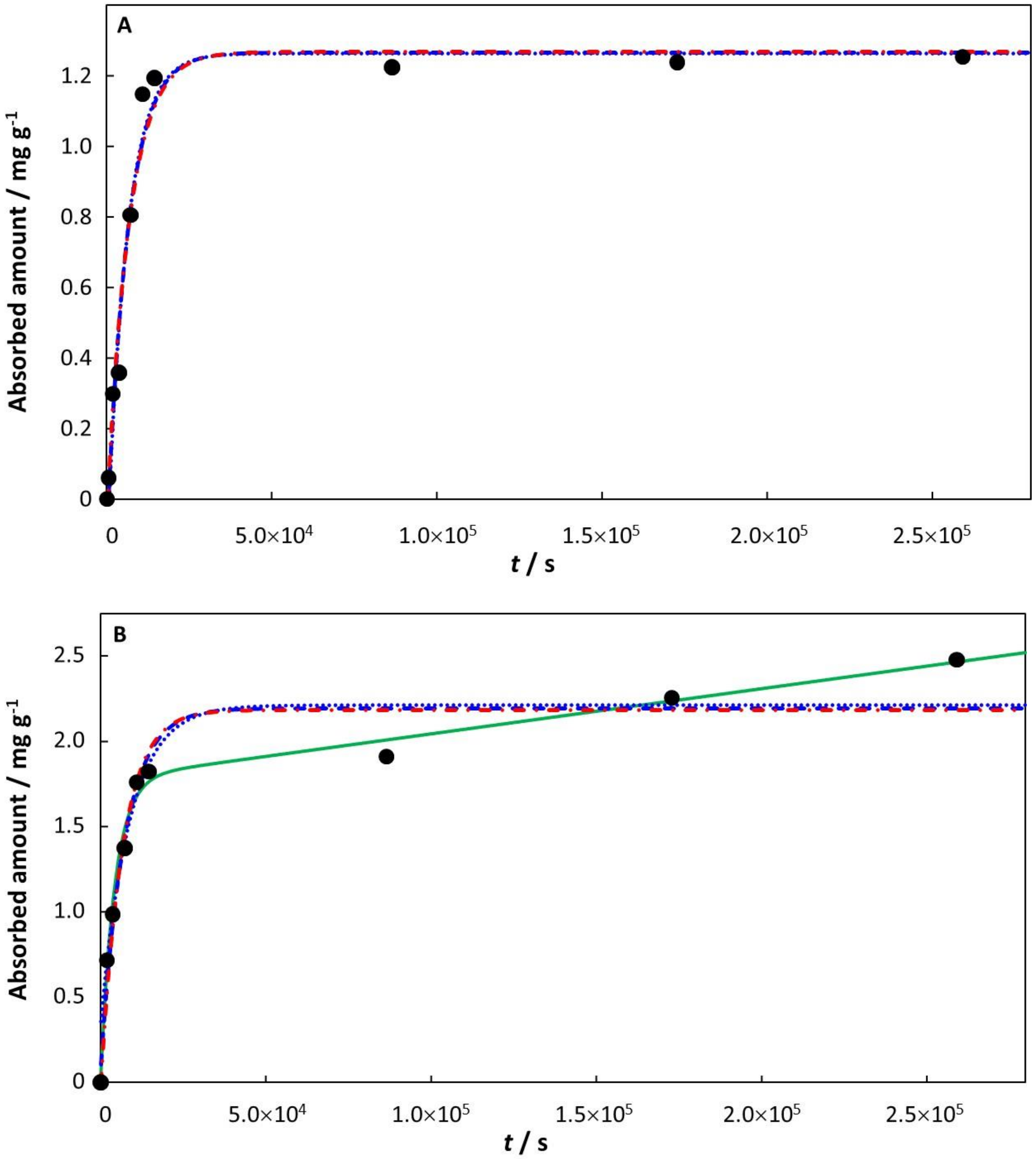

Figure S19. Absorption of oxytetracycline by **(A)** pristine PP, and **(B)** aged PP. Experimental data (black solid circles) from Ref. [47] for $c^*_{X,w}$ = 2.17×10^{-2} mol m^{-3} (10 mg dm^{-3}). Computed curves correspond to the involved integral fit, eq 18 (including the initial 0-absorption point: green solid curve), the mono-exponential, eq 21 (including the initial 0-absorption point: blue dashed line; excluding the initial 0-absorption point: blue dotted line) and the mono-exponential, eq 20 (including the initial 0-absorption point: red dot-dashed line). See main text for details of the fitting procedures.

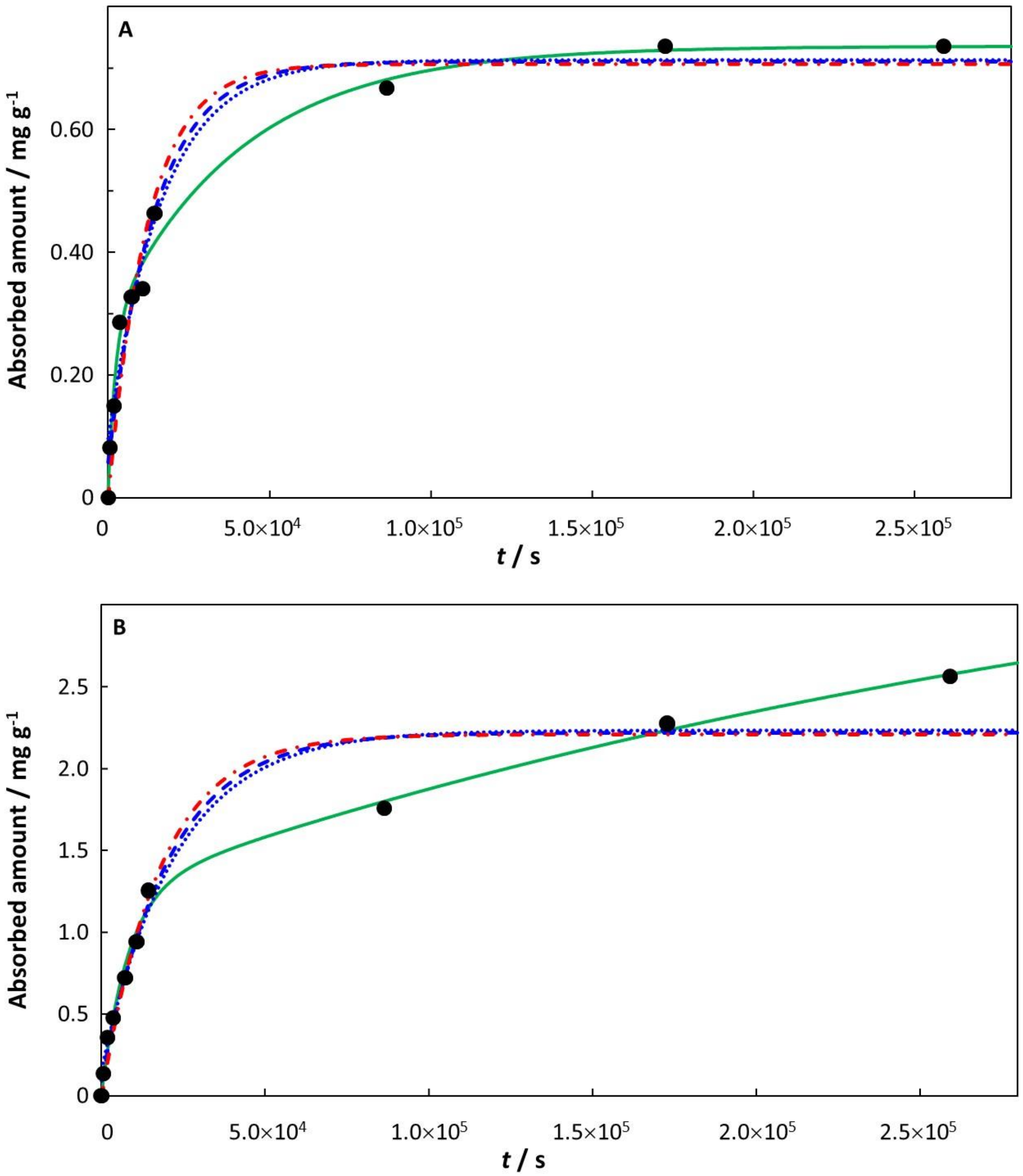

Figure S20. Absorption of chloramphenicol by **(A)** pristine PP, and **(B)** aged PP. Experimental data (black solid circles) from Ref. [47] for $c^{*}_{X,w}$ = 3.09×10^{-2} mol m^{-3} (10 mg dm^{-3}). Computed curves correspond to the involved integral fit, eq 18 (including the initial 0-absorption point: green solid curve), the mono-exponential, eq 21 (including the initial 0-absorption point: blue dashed line; excluding the initial 0-absorption point: blue dotted line) and the mono-exponential, eq 20 (including the initial 0-absorption point: red dot-dashed line). See main text for details of the fitting procedures.

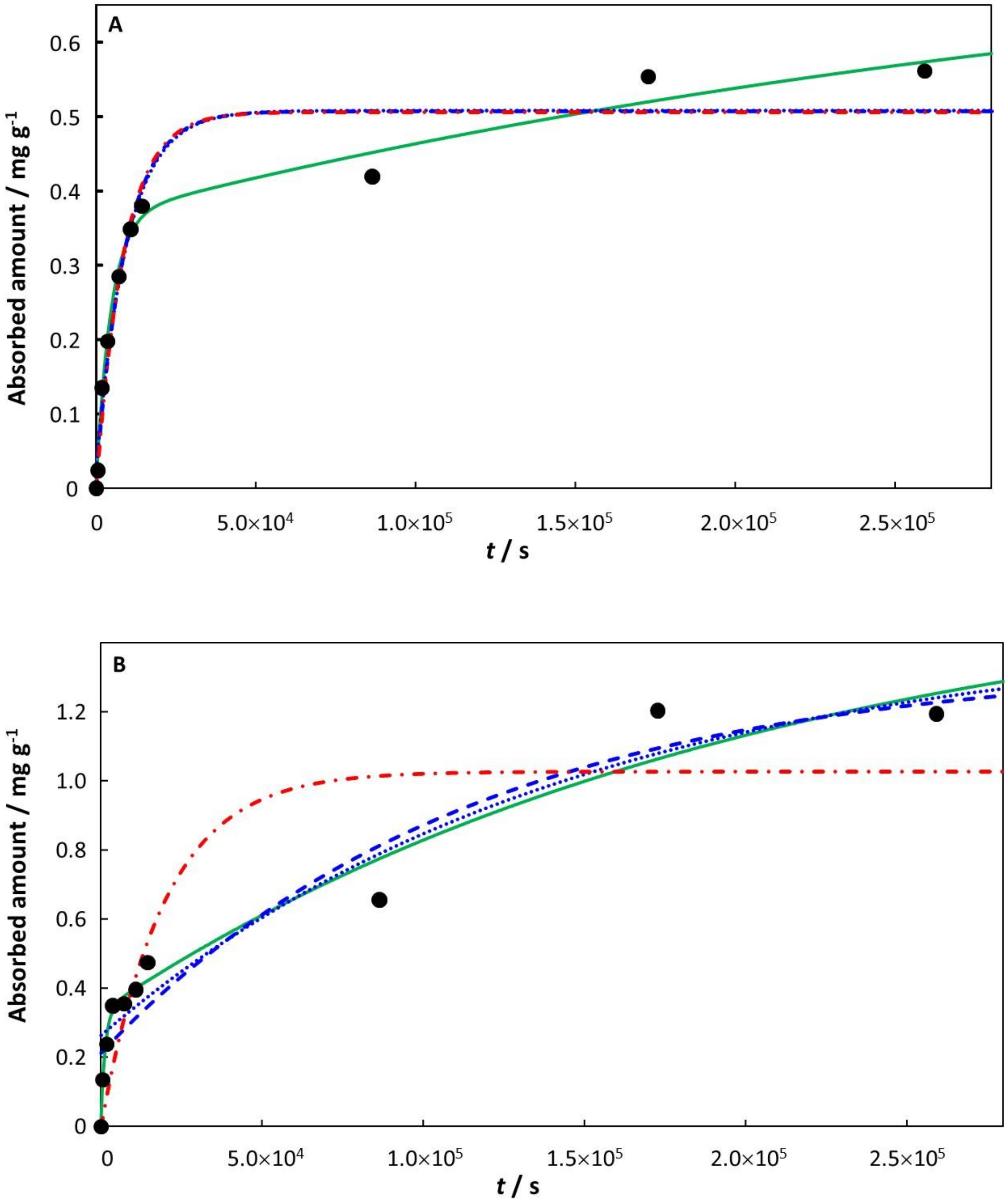

Figure S21. Absorption of enrofloxacin by **(A)** pristine PP, and **(B)** aged PP. Experimental data (black solid circles) from Ref. [47] for $c^{*}_{\mathrm{X,w}}$ = 2.78×10^{-2} mol m^{-3} (10 mg dm^{-3}). Computed curves correspond to the involved integral fit, eq 18 (including the initial 0-absorption point: green solid curve), the mono-exponential, eq 21 (including the initial 0-absorption point: blue dashed line; excluding the initial 0-absorption point: blue dotted line) and the mono-exponential, eq 20 (including the initial 0-absorption point: red dot-dashed line). See main text for details of the fitting procedures.

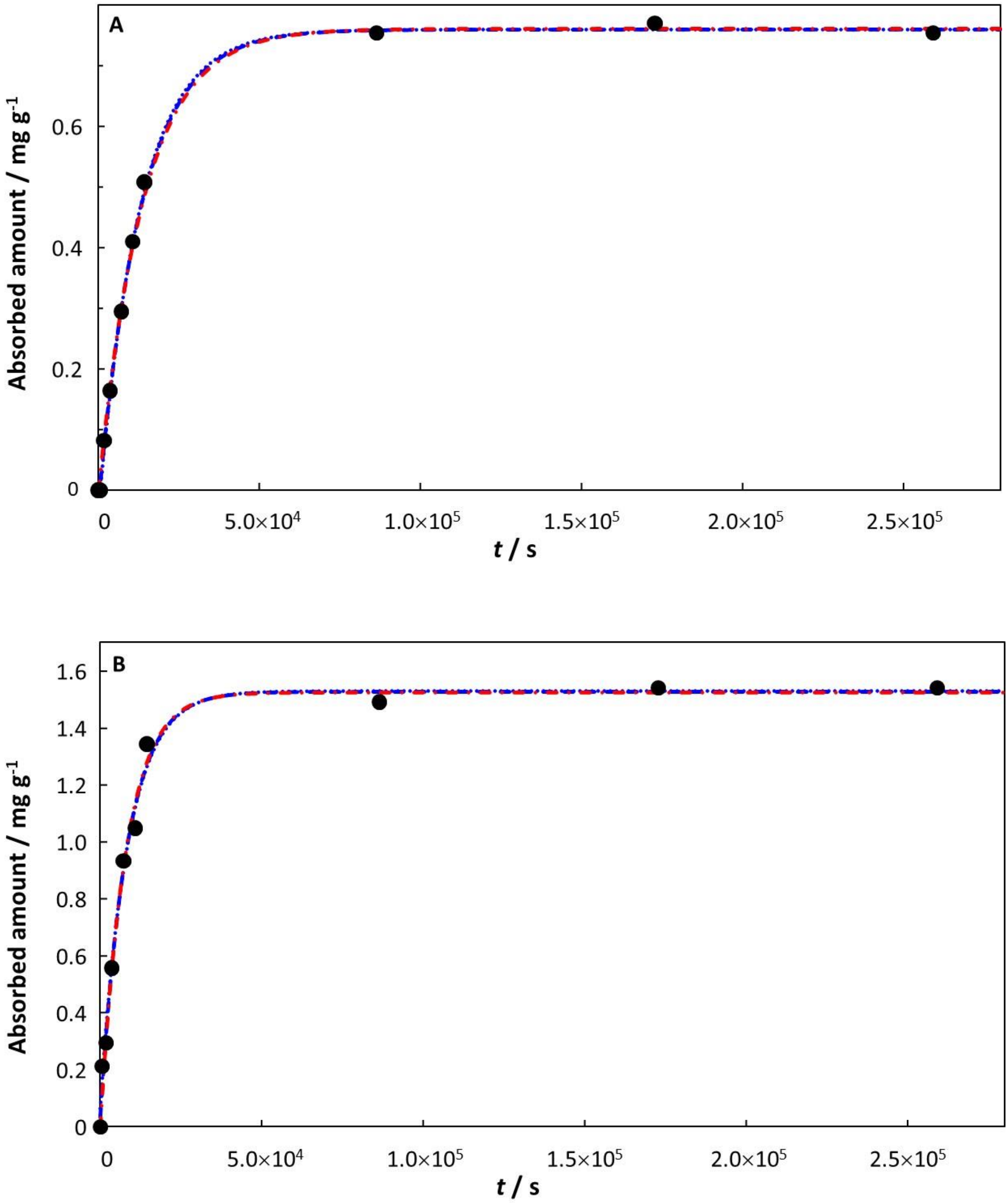

Figure S22. Absorption of ciprofloxacin by **(A)** pristine PP, and **(B)** aged PP. Experimental data (black solid circles) from Ref. [47] for $c^{*}_{X,w}$ = 3.02×10^{-2} mol m^{-3} (10 mg dm^{-3}). Computed curves correspond to the mono-exponential, eq 21 (including the initial 0-absorption point: blue dashed line; excluding the initial 0-absorption point: blue dotted line) and the mono-exponential, eq 20 (including the initial 0-absorption point: red dot-dashed line). See main text for details of the fitting procedures.

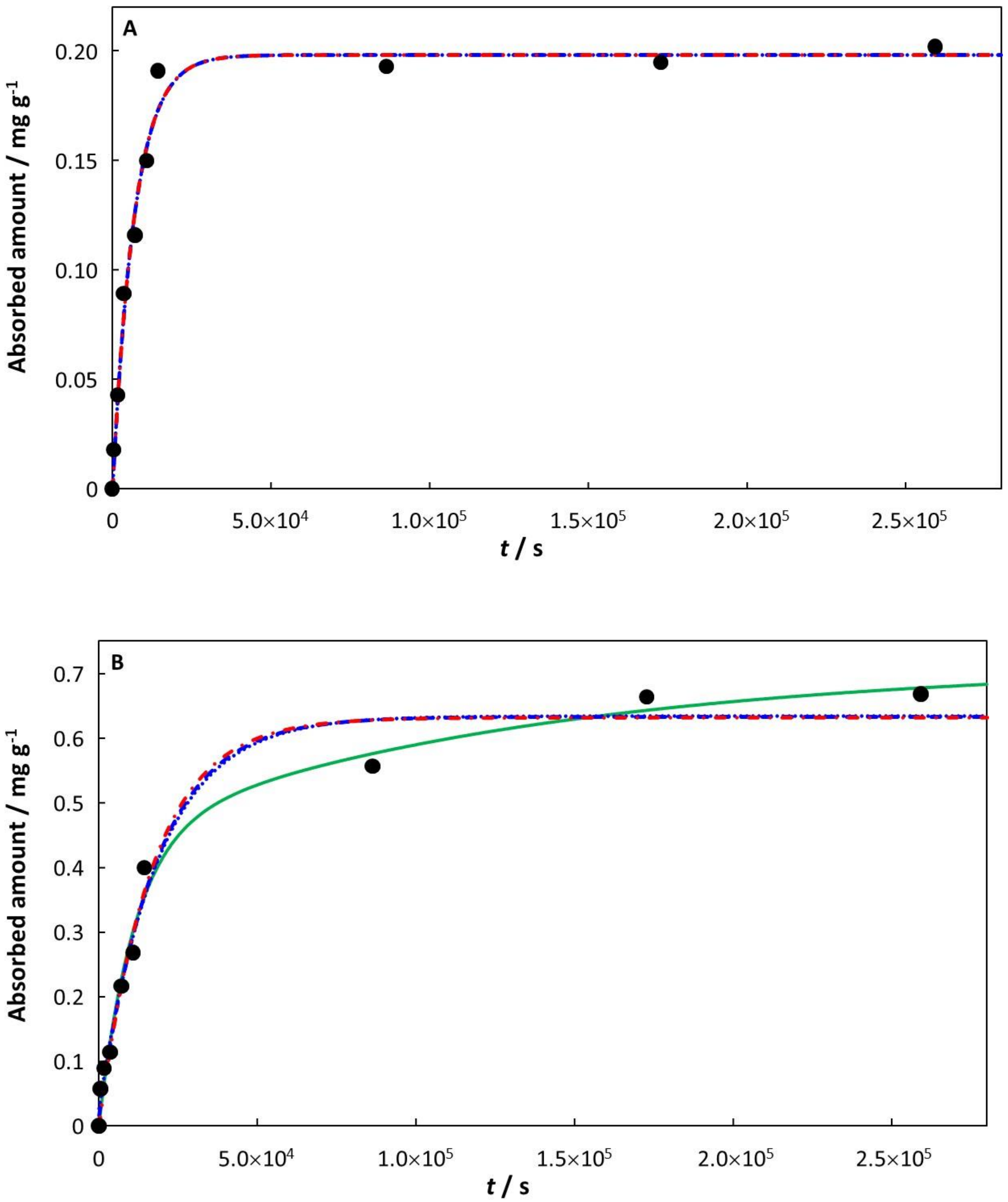

Figure S23. Absorption of ofloxacin by **(A)** pristine PP, and **(B)** aged PP. Experimental data (black solid circles) from Ref. [47] for $c^*_{\mathrm{X,w}}$ = 2.77×10^{-2} mol m^{-3} (10 mg dm^{-3}). Computed curves correspond to the involved integral fit, eq 18 (including the initial 0-absorption point: green solid curve), the mono-exponential, eq 21 (including the initial 0-absorption point: blue dashed line; excluding the initial 0-absorption point: blue dotted line) and the mono-exponential, eq 20 (including the initial 0-absorption point: red dot-dashed line). See main text for details of the fitting procedures.

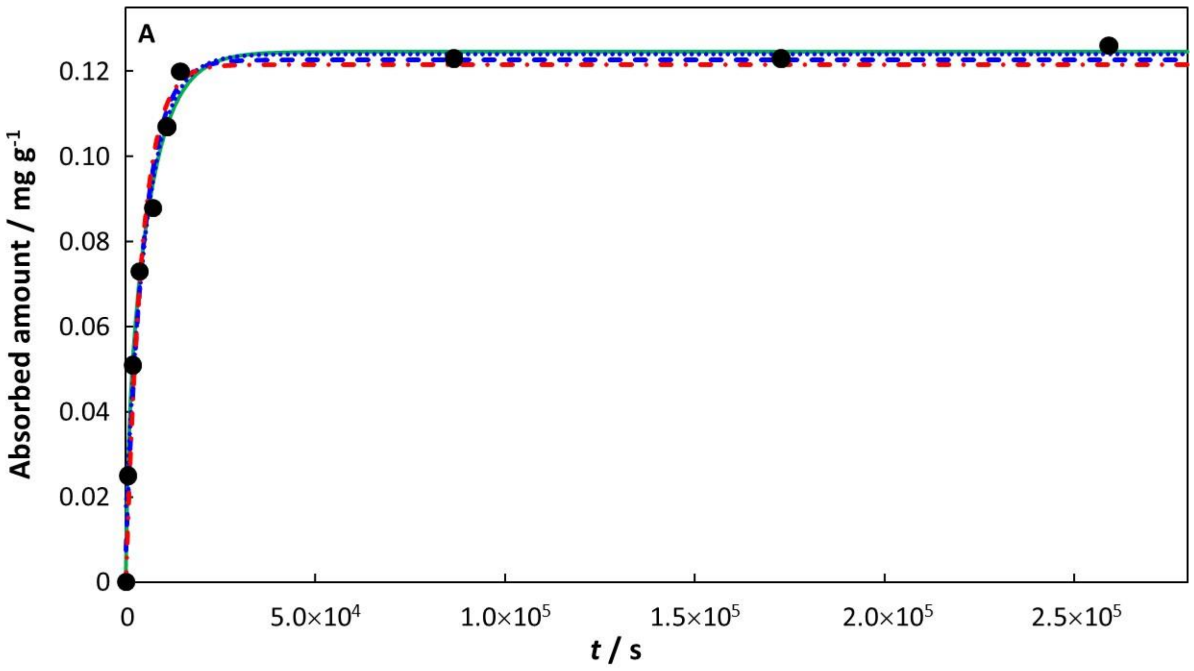

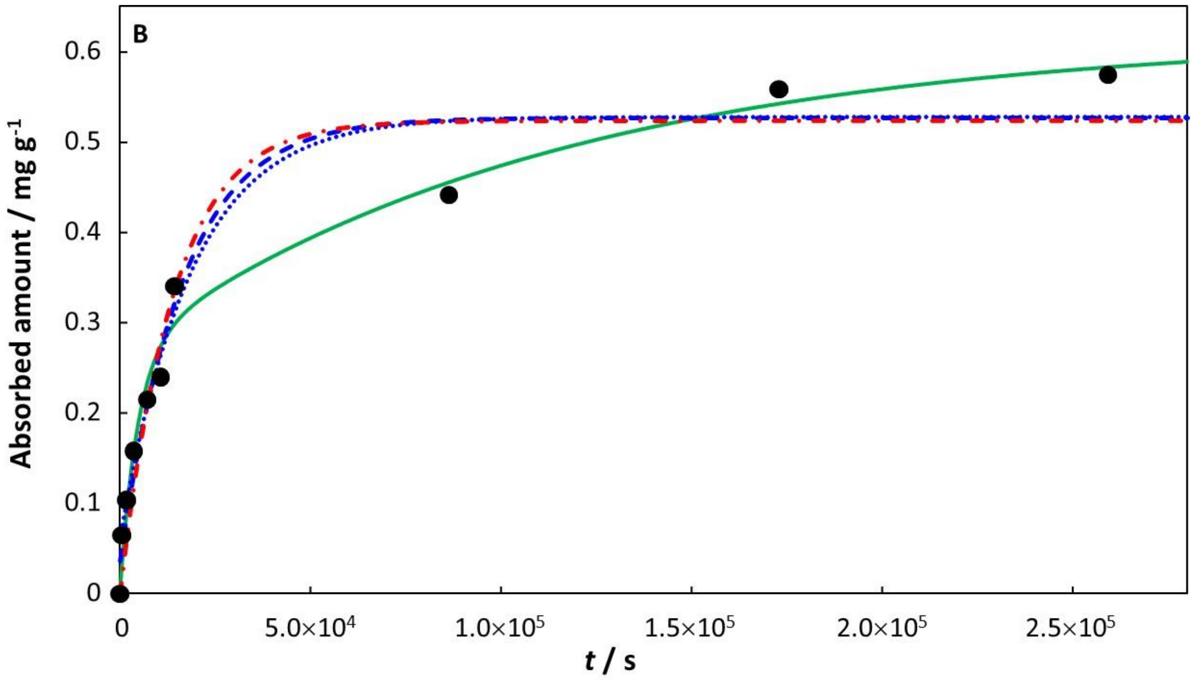

Figure S24. Absorption of norfloxacin by **(A)** pristine PP, and **(B)** aged PP. Experimental data (black solid circles) from Ref. [47] for $c^*_{X,w}$ = 3.13×10^{-2} mol m^{-3} (10 mg dm^{-3}). Computed curves correspond to the involved integral fit, eq 18 (including the initial 0-absorption point: green solid curve), the mono-exponential, eq 21 (including the initial 0-absorption point: blue dashed line; excluding the initial 0-absorption point: blue dotted line) and the mono-exponential, eq 20 (including the initial 0-absorption point: red dot-dashed line). See main text for details of the fitting procedures.

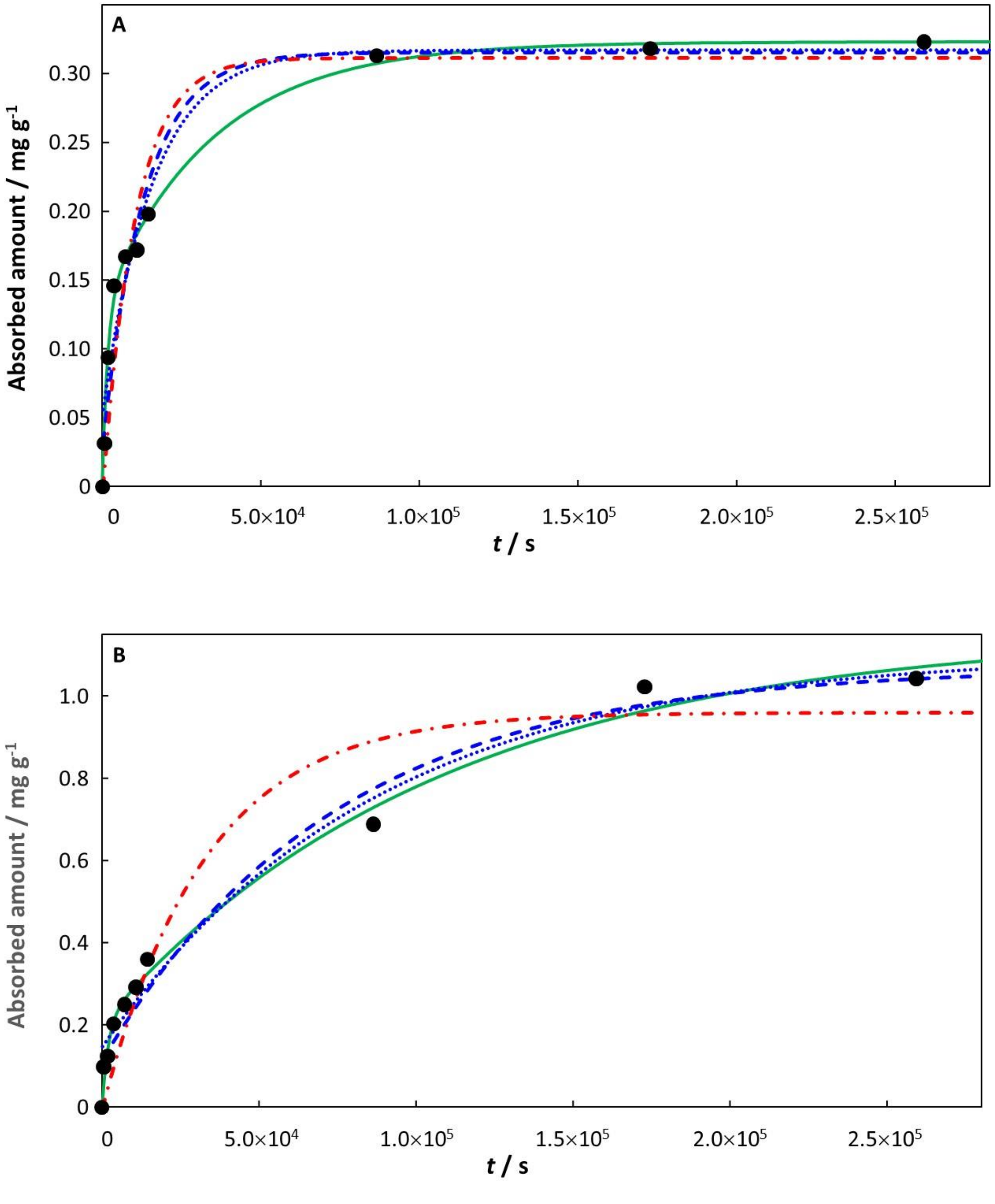

Figure S25. Absorption of sulfamerazine by **(A)** pristine PP, and **(B)** aged PP. Experimental data (black solid circles) from Ref. [47] for $c^{*}_{\mathrm{X,w}}$ = 3.78×10^{-2} mol m^{-3} (10 mg dm^{-3}). Computed curves correspond to the involved integral fit, eq 18 (including the initial 0-absorption point: green solid curve), the mono-exponential, eq 21 (including the initial 0-absorption point: blue dashed line; excluding the initial 0-absorption point: blue dotted line) and the mono-exponential, eq 20 (including the initial 0-absorption point: red dot-dashed line). See main text for details of the fitting procedures.

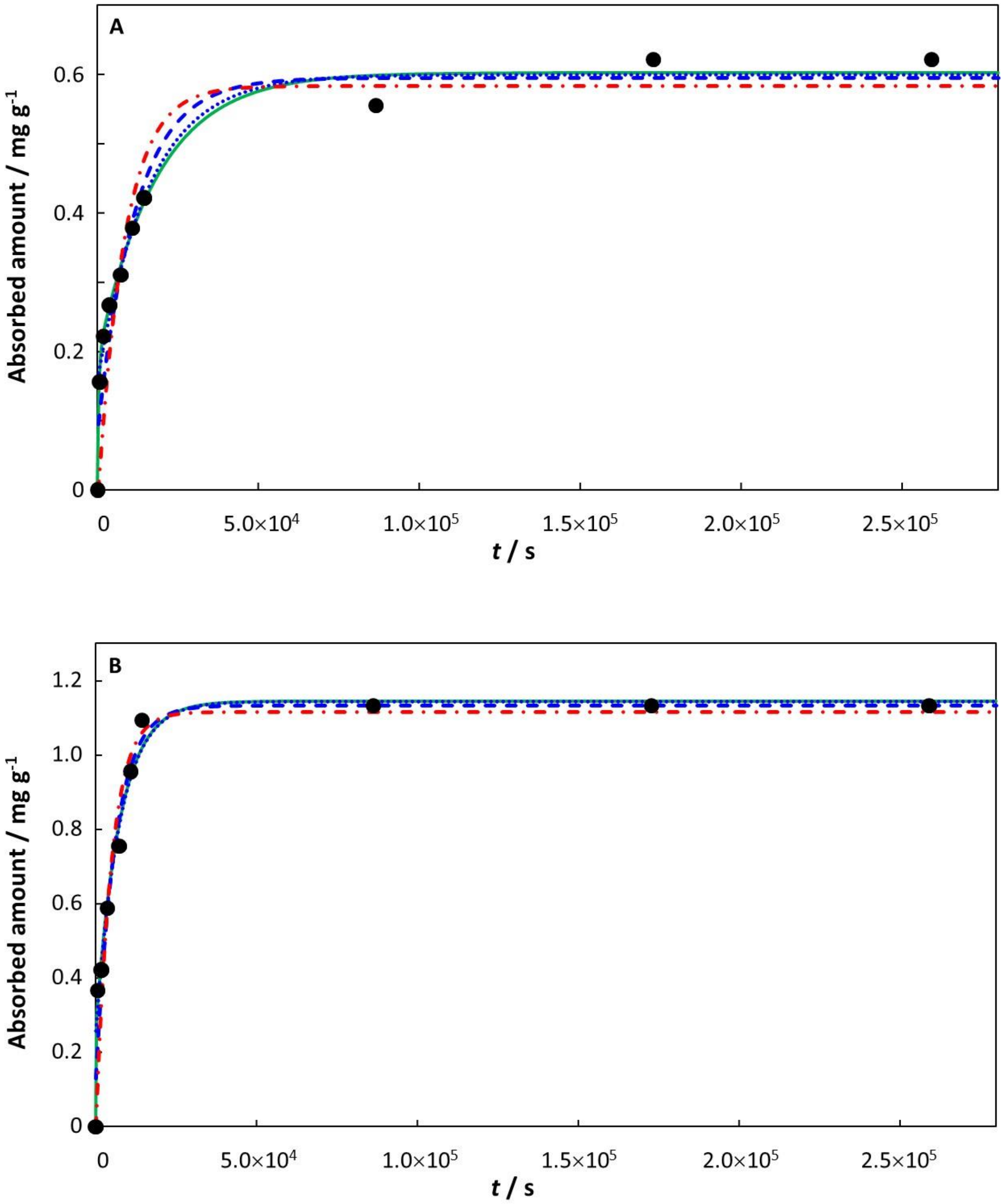

Figure S26. Absorption of sulfathiazole by **(A)** pristine PP, and **(B)** aged PP. Experimental data (black solid circles) from Ref. [47] for $c^*_{X,w}$ = 3.92×10^{-2} mol m^{-3} (10 mg dm^{-3}). Computed curves correspond to the involved integral fit, eq 18 (including the initial 0-absorption point: green solid curve), the mono-exponential, eq 21 (including the initial 0-absorption point: blue dashed line; excluding the initial 0-absorption point: blue dotted line) and the mono-exponential, eq 20 (including the initial 0-absorption point: red dot-dashed line). See main text for details of the fitting procedures.

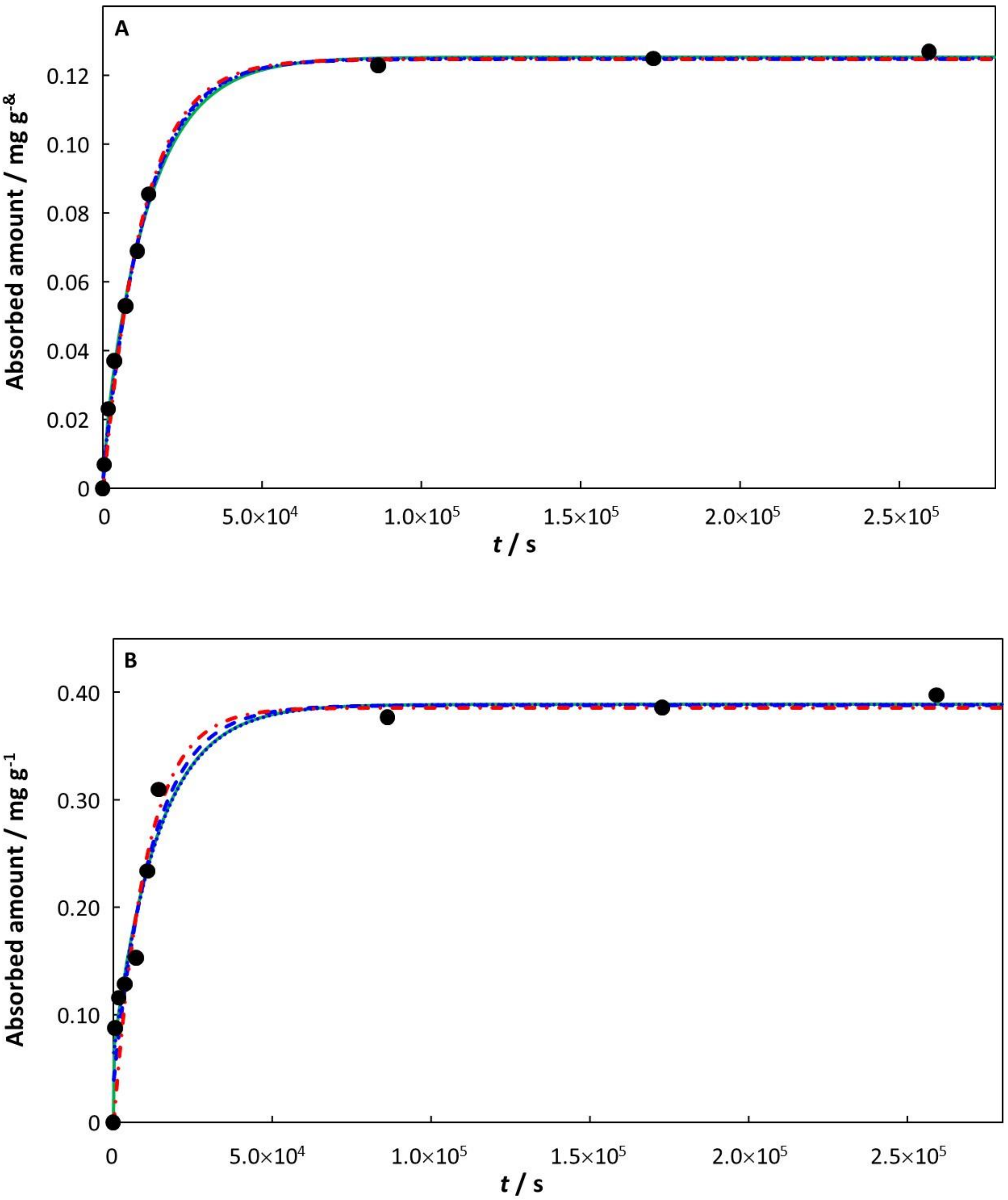

Figure S27. Absorption of tetracycline by **(A)** pristine PP, and **(B)** aged PP. Experimental data (black solid circles) from Ref. [47] for $c^*_{\mathrm{X,w}}$ = 2.25×10^{-2} mol m^{-3} (10 mg dm^{-3}). Computed curves correspond to the involved integral fit, eq 18 (including the initial 0-absorption point: green solid curve), the mono-exponential, eq 21 (including the initial 0-absorption point: blue dashed line; excluding the initial 0-absorption point: blue dotted line) and the mono-exponential, eq 20 (including the initial 0-absorption point: red dot-dashed line). See main text for details of the fitting procedures.

References

(S1) von Stackelberg, M. O.; Pilgram, M.; Toome, V. Bestimmung von Diffusionskoeffizienten einiger Ionen in wäßriger Lösung in Gegenwart von Fremdelektrolyten. I. *Z. Elektrochem.* **1953**, *57*, 342-350.

(S2) Di Cagno, M. P.; Clarelli, F.; Våbeno, J.; Lesley, C.; Rahman S. D.; Cauzzo, J.; Franceschinis, E.; Realdon, N.; Stein, P. C. Experimental determination of drug diffusion coefficients in unstirred aqueous environments by temporally resolved concentration measurements. *Molec. Pharm.* **2018**, *15*, 1488-1494.

(S3) Urík, J.; Paschke, A.; Vrana, B. Diffusion coefficients of polar organic compounds in agarose hydrogel and water and their use for estimating uptake in passive samplers. *Chemosphere* **2020**, *249*: 126183.

(S4) Rudnicki, K.; Poltorak, L.; Skrzypek, S.; Sudhölter, E. J. R. Ion transfer voltammetry for analytical screening of fluoroquinolone antibiotics at the water – 1,2-dichloroethane interface. *Anal. Chim. Acta* **2019**, *1085*, 75-84.

(40) Lang, M.; Yu, X.; Liu, J.; Xia, T.; Wang, T.; Jia, H.; Guo, X. Fenton aging significantly affects the heavy metal adsorption capacity of polystyrene microplastics. *Sci. Total Environ.* **2020**, *722*: 137762.

(41) Guo, C.; Wang, L.; Lang, D.; Qian, Q.; Wang, W.; Wu, R.; Wang, J. UV and chemical aging alter the adsorption behavior of microplastics for tetracycline. *Environ. Poll.* **2023**, *318*: 120859.

(42) Wang, Y.; Liu, C.; Wang, F.; Sun, Q. Behavior and mechanism of atrazine adsorption on pristine and aged microplastics in the aquatic environment: kinetic and thermodynamic studies. *Chemosphere* **2022**, *292*: 133425.

(43) Liu, G.; Zhu, Z.; Yang, Y.; Sun, Y.; Yu, F.; Ma, J. Sorption behavior and mechanism of hydrophilic organic chemicals to virgin and aged microplastics in freshwater and seawater. *Environ. Poll.* **2019**, *246*, 26-33.

(44) Gao, L.; Fu, D.; Zhao, J.; Wu, W.; Wang, Z.; Su, Y.; Peng, L. Microplastics aged in various environmental media exhibited strong sorption to heavy metals in seawater. *Mar. Poll. Bull.* **2021**, *169*: 112480.

(45) Zhang, Y.; Chen, Z.; Shi, Y.; Ma, Q.; Mao, H.; Li, Y.; Wang, H.; Zhang, Y. Revealing the sorption mechanisms of carbamazepine on pristine and aged microplastics with extended DLVO theory. *Sci. Total Environ.* **2023**, *874*: 162480.

(46) Xue, X.-D.; Fang, C.-R.; Zhuang, H.-F. Adsorption behaviors of the pristine and aged thermoplastic polyurethane microplastics in Cu(II)-OTC coexisting system. *J. Haz. Mater.* **2021**, *407*: 124835.

(47) Yao, J.; Wen, J.; Li, H.; Yang, Y. Surface functional groups determine adsorption of pharmaceuticals and personal care products on polypropylene microplastics. *J. Haz. Mater.* **2022**, *423*: 127131.

(48) Chen, C.; Pang, X.; Chen, Q.; Xu, M.; Xiao, Y.; Wu, J.; Zhang, Y.; Liu, Y.; Long, L.; Yang, G. Tetracycline adsorption trajectories on aged polystyrene in a simulated aquatic environment: a mechanistic investigation. *Sci. Total Environ.* **2022**, *851*: 158204.
